# Global population structure and adaptive evolution of aflatoxin‐producing fungi

**DOI:** 10.1002/ece3.3464

**Published:** 2017-09-30

**Authors:** Geromy G. Moore, Rodrigo A. Olarte, Bruce W. Horn, Jacalyn L. Elliott, Rakhi Singh, Carolyn J. O'Neal, Ignazio Carbone

**Affiliations:** ^1^ Southern Regional Research Center Agricultural Research Service U.S. Department of Agriculture New Orleans LA USA; ^2^ Department of Plant Biology University of Minnesota St. Paul MN USA; ^3^ Department of Agriculture Agricultural Research Service National Peanut Research Laboratory Dawson GA USA; ^4^ Department of Entomology and Plant Pathology Center for Integrated Fungal Research North Carolina State University Raleigh NC USA

**Keywords:** *Aspergillus*, balancing selection, maximum likelihood, multilocus sequence typing, principal component analysis

## Abstract

Aflatoxins produced by several species in *Aspergillus* section *Flavi* are a significant problem in agriculture and a continuous threat to human health. To provide insights into the biology and global population structure of species in section *Flavi*, a total of 1,304 isolates were sampled across six species (*A. flavus, A. parasiticus, A. nomius, A. caelatus, A. tamarii,* and *A. alliaceus*) from single fields in major peanut‐growing regions in Georgia (USA), Australia, Argentina, India, and Benin (Africa). We inferred maximum‐likelihood phylogenies for six loci, both combined and separately, including two aflatoxin cluster regions (*aflM/alfN* and *aflW/aflX*) and four noncluster regions (*amdS, trpC, mfs* and *MAT*), to examine population structure and history. We also employed principal component and STRUCTURE analysis to identify genetic clusters and their associations with six different categories (geography, species, precipitation, temperature, aflatoxin chemotype profile, and mating type). Overall, seven distinct genetic clusters were inferred, some of which were more strongly structured by G chemotype diversity than geography. Populations of *A. flavus* S in Benin were genetically distinct from all other section *Flavi* species for the loci examined, which suggests genetic isolation. Evidence of trans‐speciation within two noncluster regions, whereby *A. flavus* S_BG_ strains from Australia share haplotypes with either *A. flavus* or *A. parasiticus*, was observed. Finally, while clay soil and precipitation may influence species richness in *Aspergillus* section *Flavi*, other region‐specific environmental and genetic parameters must also be considered.

## 1 INTRODUCTION

*Aspergillus* section *Flavi* contains the most serious mycotoxin‐producing fungi in this genus, being named for one of its well publicized toxigenic species, *Aspergillus flavus* Link (Horn, 2003; Raper & Fennell, 1965). Being a prolific colonizer of many taxonomic groups (e.g., plants, animals, and humans), *A. flavus* is distributed worldwide and occupies different ecological niches (Cleveland et al., 2009; Klich, 2007). *A. flavus*, as a species, encompasses two morphotypes and various chemotypes. Some strains produce large sclerotia (L‐strain) while others produce numerous small sclerotia (S‐strain) (Cotty, 1989). *A. flavus* L‐strains can exhibit either a nonaflatoxigenic phenotype or they will produce only one type (B) of aflatoxins, and the S‐strain morphotype will produce only B aflatoxins (S_B_), or they can produce B and G aflatoxins (S_BG_) (Cotty & Cardwell, 1999). Production of G aflatoxins is rarely observed in *A. flavus* L‐strains (Ehrlich et al., 2004). Most of the other species in section *Flavi* are soil‐inhabiting saprobes such as *A. parasiticus*, another producer of mycotoxins (Abbas et al., 2009; Horn, 2003). Aflatoxins are the most studied of the mycotoxins in this genus (Bennett, 2010; Horn, 2003); in fact the term “mycotoxin” was coined to describe aflatoxins after the Turkey X disease outbreak in the 1960s (Bennett & Klich, 2003). In developing countries, the risk to humans by carcinogenic aflatoxins is very high, and chronic exposure to aflatoxin is estimated to impact 4.5 billion people (CDC 2016). Strict regulations are being set worldwide, which means the need for effective control of aflatoxin‐producing fungi is crucial (Klich, 2007). Recent discoveries of the sexual states in *A. flavus*,* A. nomius* and *A. parasiticus* suggest that barriers to genetic recombination, such as heterokaryon incompatibility, are not impassable (Horn, Gell, Singh, Sorensen, & Carbone, 2016; Horn, Moore, & Carbone, 2011; Horn, Ramirez‐Prado, and Carbone, 2009a; Horn, Ramirez‐Prado, and Carbone, 2009b; Horn, Moore et al., 2009; Micali & Smith, 2005; Ramirez‐Prado et al., 2008), and genetic exchange and recombination could potentially create new strains or species that are better adapted to particular niches (Milgroom, 1996). Geiser and coworkers reported two reproductively isolated clades (identified as Groups I and II) within *A. flavus* for an Australian population, and they suggested that recombination could be occurring among strains within the species (Geiser, Pitt, & Taylor, 1998). Geiser's Group I included *A. flavus* L and S_B_ strains, and Group II included *A. flavus* S_B_ and S_BG_ strains (Geiser et al., 1998). In the case of the two approved *A. flavus* biocontrol strains that are both nonaflatoxigenic, genetic recombination may restore toxigenicity (Geiser et al., 1998; Horn et al., 2016; Moore et al., 2009). In an experimentally recombining population of *A. flavus*, a single crossover recombination was shown to result in the gain of cluster regions that were able to restore the aflatoxigenic phenotype (Olarte et al., 2011); field experiments further showed that *A. flavus* sclerotia can be fertilized by native strains and yield recombinant progeny (Horn et al., 2016). Moreover, genetic exchange between species may result in hybrids that have novel chemotype profiles, are mycotoxin super‐producers, are better pathogens, and/or are more fit under adverse and changing environments; recent evidence shows hybridization is experimentally possible between *A. flavus* and *A. parasiticus* (Olarte et al., 2015).

Balancing selection acts to maintain genetic polymorphisms in a population and results in more genetic variation between alleles or haplotypes within a species than would be predicted by genetic drift alone (Hedrick, 2007; Schierup, Mikkelsen, & Hein, 2001). This signature of balancing selection has been inferred for the mating‐type loci of various fungi (May et al., 1999; Ramirez‐Prado et al., 2008), transcending species boundaries and resulting in trans‐speciation, a phenomenon where the loci under balancing selection are more similar among species than within a single species. Other evolutionary mechanisms that may result in a signature of trans‐speciation include hybridization and introgression, or incomplete lineage sorting, at specific loci (Sun, Corcoran et al., 2012). Trans‐speciation has been observed for chemotype‐specific alleles in *A. parasiticus* (Carbone et al., 2007) and *A. flavus* (Moore et al., 2009). In the aflatoxin gene cluster, selection may act to maintain the nonaflatoxigenic phenotype in *A. flavus*, and G_1_ or B_1_ dominance in *A. parasiticus* (Carbone et al., 2007; Moore et al., 2009). Because balancing selection could be acting on chemotype or morphological differences (Moore et al., 2009; Nei, 2007), species delimitation using cluster regions alone is tenuous, and a more holistic approach is warranted (Samson & Varga, 2009). For phylogenetic inference of species trees, multiple single‐copy neutral loci such as *amdS* and *trpC* should also be targeted (Michielse, Ram, & van den Hondel, 2004; Yelton, Hamer, & Timberlake, 1984).

Climate change can result in fragmentations and bottlenecks, eliminating or reducing the population sizes of endemic species, and selecting for more fit taxa (Ali & Roossinck, 2008; Opdam & Wascher, 2004). Climate has been reported to influence the aflatoxin producing ability of *A. flavus*, alter the numbers of aflatoxigenic fungi in the environment, and change fungal population structure (Cotty & Jaime‐Garcia, 2007). Soil type (e.g., clay, sand) and quality (i.e., edaphic factors such as pH and mineral content) can impact the presence and distribution of *Aspergillus* in a field (Ahmad & Singh, 1994; Wassila, Houda, & Mohamed, 2015). For example, it has been reported that soils high in clay content may favor the prevalence of the *A. flavus* S morphotype (Jaime‐Garcia & Cotty, 2006). Given the diverse habitats and differences in mean annual temperature and precipitation in peanut‐growing regions worldwide, inferences of genetic isolation should be examined with the possibility of climatic and environmental conditions driving genetic differentiation and adaptation (Moore et al., 2013). In this study, we examine the evolutionary history of populations of *Aspergillus* section *Flavi* from geographically isolated regions using a bottom‐up microevolutionary approach. We build on previously obtained and published data (Moore et al., 2013), to test whether there are significant associations of haplotypes and clades with chemotype production and with different abiotic and edaphic factors (e.g., soil type, precipitation, temperature). We found that while diversity in aflatoxin chemotype profile is a good predictor of species genetic diversity, our sampled fields that have clay soils, as well as high precipitation, exhibited less species diversity. Further exploration of the role of climate and other environmental parameters in adaptive evolution in *Aspergillus* section *Flavi* will allow us to better understand, and possibly improve, our biocontrol strategies of these agriculturally important species in different parts of the world.

## 2 MATERIALS AND METHODS

### 2.1 Sampling, DNA isolation, and target loci

Species in *Aspergillus* section *Flavi* were sampled from geographically isolated peanut fields spanning five continents (Tables 1 and S1). We focused our attention on major peanut‐growing regions that were considered hotspots for aflatoxin contamination in 2005. As our goal was to maximize our sampling of genetic diversity in *Aspergillus* section *Flavi*, we sampled species diversity at southern latitudes, which is typically higher than in northern latitudes (Horn, 2007). For this reason, the European continent is not represented in our sampling. *Aspergillus oryzae* isolates from Japan were included in this global study of section *Flavi,* although they were not sampled in peanut fields. A total of 1,304 isolates were examined for this study. DNA was extracted from freeze‐dried mycelia, and target loci for phylogenetic inference were amplified and sequenced, as described previously (Moore et al., 2009). DNA sequence variation was assayed in six genomic regions: *aflM/aflN* and *aflW/aflX* intergenic regions within the aflatoxin gene cluster, and a major facilitator superfamily (*mfs*) gene adjacent to the cluster on chromosome 3; acetamidase (*amdS*) and mating‐type (*MAT*) genes on chromosome 6; and the tryptophan synthase (*trpC*) gene on chromosome 4. The chromosomal locations associated with these loci have been reported for *A. flavus* (Moore et al., 2013; Ramirez‐Prado et al., 2008; Smith, Woloshuk, Robertson, & Payne, 2007) but have not been confirmed for all species examined in this study. For each locus, the sequence of at least one representative isolate from each putative species was subjected to BLASTn searches against the NCBI nonredundant (nr) database to confirm species identity. Table 2 lists the numbers of individuals in each species examined per locus. Mating‐type distributions and chemotype profiles for the global populations of *A. flavus* and *A. parasiticus* included in this study have been previously examined as well as field data relating to soil type, temperature and precipitation (Moore et al., 2013).

**Table 1:** Species and total individual counts for each geographic region

	Argentina	Australia	Benin	India	Japan	USA
*A. alliaceus*	0	9	0	0	0	0
*A. caelatus*	80	0	0	0	0	31
*A. flavus* L	80	80	80	80	0	104
*A. flavus* S	4	80	44	0	0	26
*A. nomius*	0	0	0	0	0	33
*A. oryzae*	0	0	0	0	51	1
*A. parasiticus*	80	80	1	0	0	182
*A. sojae*	0	0	0	0	1	0
*A. tamarii*	0	6	80	56	0	35
Total	244	255	205	136	52	412

**Table 2:** Species and total isolate and haplotype counts for each genomic region

	*aflM/aflN*	*aflW/aflX*	*amdS*	*trpC*	*mfs*	*MAT1‐1*	*MAT1‐2*
*A. alliaceus*	4 (1)	5 (3)	1 (1)	1 (1)	1 (1)	2 (2)	2 (2)
*A. caelatus*	n/a	42 (12)	40 (8)	55 (9)	46 (8)	56 (5)	19 (3)
*A. flavus* L	346 (29)	357 (17)	352 (15)	352 (13)	351 (11)	192 (2)	178 (4)
*A. flavus* S	90 (28)	90 (15)	89 (13)	89 (9)	89 (11)	39 (4)	48 (5)
*A. nomius*	1 (1)	19 (2)	7 (4)	9 (4)	0	15 (9)	24 (15)
*A. oryzae*	34 (5)	49 (12)	34 (2)	34 (3)	34 (2)	32 (3)	7 (1)
*A. parasiticus*	243 (24)	245 (10)	245 (9)	255 (4)	245 (16)	208 (5)	68 (1)
*A. sojae*	1 (1)	1 (1)	1 (1)	1 (1)	1 (1)	0	0
*A. tamarii*	n/a	35 (6)	84 (11)	66 (3)	1 (1)	36 (6)	68 (6)
Total	719 (89)	844 (78)	854 (64)	862 (47)	768 (51)	580 (36)	408 (37)

The number of haplotypes is shown in parentheses.

### 2.2 Phylogenetic inference, principal component, and structure analysis

The GenBank accessions for sequences from each locus are listed by species in Tables [Link], [Link], [Link], [Link], [Link], [Link], [Link], [Link]. Mutliple DNA sequence alignment was performed using Sequencher^®^ version 5.0 software (Gene Codes Corporation, Ann Arobor, MI, USA), and each alignment was refined by eye. The alignments were then exported in NEXUS format for use in SNAP Workbench (Monacell & Carbone, 2014; Price & Carbone, 2005). Multiple sequence alignments for each locus were collapsed into haplotypes using SNAP Map (Aylor, Price, & Carbone, 2006). Collapsing was performed with the options of recoding indels and excluding infinite sites violations. Indels were recoded as single unique events and weighted equally in phylogenetic analyses. Recoded indels that were not binary violated infinites sites and were excluded. Because our variation spans the population–species interface, collapsing in this fashion allowed us to take full advantage of indels arising within species and exclude only those indels that violate position homology between species. All but one of the alignments were rooted with *A. nomius* type strain NRRL 13137 as an outgroup species, an appropriate outgroup according to Peterson et al. (2008). Since NRRL 13137 was found to contain only the *MAT1‐1* idiomorph based on genomic sequencing (Moore, Mack, & Beltz, 2015), a *MAT1‐2 A. nomius* isolate (IC1493) was used for the respective phylogeny. We performed heuristic parsimony searches in PAUP* 4.0 (Swofford, 1998) to infer phylogenies for all section *Flavi* species sampled, and if parsimony searches inferred more than one equally parsimonious tree, bootstrap values were calculated for branch length support. Bootstrap consensus trees were based on 1,000 replicates. Statistical support for specific clades in the tree was based on branches with bootstrap percentages greater than 70 (Hillis & Bull, 1993). For the *amdS* and *trpC* loci, a haplotype comparison was made through separate alignments (data not shown) between our sampled global species and Geiser's Groups I and II (Geiser et al., 1998). Briefly, Geiser and coworkers examined 11 genomic loci for an Australian *A. flavus* population and reported the existence of two reproductively isolated clades or groups that could not be delineated by geography or morphology.

Additional single‐locus studies, for the sampled global species in section *Flavi*, involved inference of maximum‐likelihood (ML) phylogenies using the program Randomized Axelerated Maximum Likelihood or RAxML (Stamatakis, 2014), and principal component analysis (PCA). These analyses were performed with the exclusion of indels and infinite sites violations from a DNA sequence alignment. PCA uses a variance–covariance matrix to reduce a sample into groupings of uncorrelated variables called principal components (Abdi & Williams, 2010). The components are known as eigenvectors and refer to different levels of variation. The first eigenvector considers the most variation possible with subsequent eigenvectors having less variation. Principal components were normalized to sum to 1, and the number of significant axes of variation (eigenvectors) was determined using the Tracy–Widom statistic (Tracy & Widom, 1994). The number of *k* clusters was estimated using the Gap Statistic (Tibshirani, Walther, & Hastie, 2001), which is an unbiased estimate of the number of distinct clusters in the population sample. Significant principal components and PCA clusters were displayed graphically using the SCATTERPLOT3D package in R (Ligges & Mächler, 2003). In a previous study, we showed evidence of balancing selecting in the *aflW/aflX* cluster region, which delimited two evolutionary distinct lineages: IB and IC. Lineage IB included only nonaflatoxigenic isolates while IC included both toxigenic and atoxigenic strains (Moore et al., 2009). To explore this partitioning of variation into lineages IB and IC on a global scale, and across multiple species in section *Flavi*, single‐locus phylogenies were inferred for the *aflW/aflX* region, as well as noncluster loci (*amdS*,* trpC* and *MAT*).

Multilocus analyses were performed for the *A. flavus* and *A. parasiticus* populations. The *A. flavus* isolates included both large sclerotium (L) and small sclerotium (S) morphotypes (Cotty, 1989). The six genomic loci were first concatenated using SNAP Combine (Aylor et al., 2006), and collapsed with SNAP Map having recoded indels and excluded infinite sites violations. Each geographic location was examined separately with focus on *A. flavus* and *A. parasiticus* isolates and their associated chemotype profiles. Phylogenies were displayed in circle tree format using FigTree v1.3.1 (http://tree.bio.ed.ac.uk/software/figtree/) or T‐BAS v2.0 (Carbone, unpublished), an extension of the T‐BAS v1.0 toolkit (Carbone et al., 2016). Clade groupings were labeled along the tree perimeter based on species or morphotype (e.g., *A. flavus* L, *A. flavus* S, *A. parasiticus*). The PCA scatter plots considered up to three eigenvectors (axes of variation). Additional PCA scatter plots were inferred for the global combined multilocus dataset to investigate possible associations of temperature, precipitation, or soil type with inferred clusters.

The optimal number of *k* clusters was also determined using STRUCTURE version 2.3.1 (Falush, Stephens, & Pritchard, 2003; Pritchard, Stephens, & Donnelly, 2000), which assigns individuals to clusters assuming a model of admixture and correlated allele frequencies. To achieve good mixing and convergence, we used an MCMC sampling strategy of 20,000 iterations after a burn‐in period of 20,000. Three simulations were performed for *k* ranging from 1 to 10 to assess convergence of log‐likelihood values using the “full search” method in CLUMPP v1.1.2 (Jakobsson & Rosenberg, 2007). Two methods, LnP(D) and delta *K* (Evanno, Regnault, & Goudet, 2005) implemented in Structure Harvester v0.6.93 (Dent & vonHoldt, 2012), were used to estimate the optimal number of *k* clusters. The results were visualized in T‐BAS v2.0 as outer rings of a multilocus global ML phylogeny. This holistic analysis combined results from PCA, STRUCTURE, and phylogenetic inference. The ML analysis was performed using RAxML version 8, which is accessible through the CIPRES RESTful application (CRA) programmer interface (Miller et al., 2015). Specifically, the multilocus sequence alignment file was subjected to a ML search strategy under the GTRGAMMA model of rate heterogeneity, and branch support values were based on rapid bootstrapping using 500 replicates; *A. nomius* (NRRL 13137) was specified as the outgroup. The best ML tree and associated locality and chemotype metadata were examined using T‐BAS v2.0 to look for evidence of population structuring based on geography and among closely related species (*A. nomius*,* A. oryzae* and *A. sojae*). We performed statistical analyses to determine how well individual cluster membership can predict chemotype using multiple linear regressions in R (R Development Core Team 2014) on the matrix of individual membership coefficients and different toxin chemotype concentrations, normalized between 0 and 1. Population structure was investigated by hierarchical analysis of molecular variance (AMOVA) and quantifying total variance within and between localities by *F*_ST_ using Arlequin v.3.5.1.2 (Excoffier & Lischer, 2010). Significance of *F*_ST_ estimates was determined using 1,000 permutations.

## 3 RESULTS

**Figure 1:**
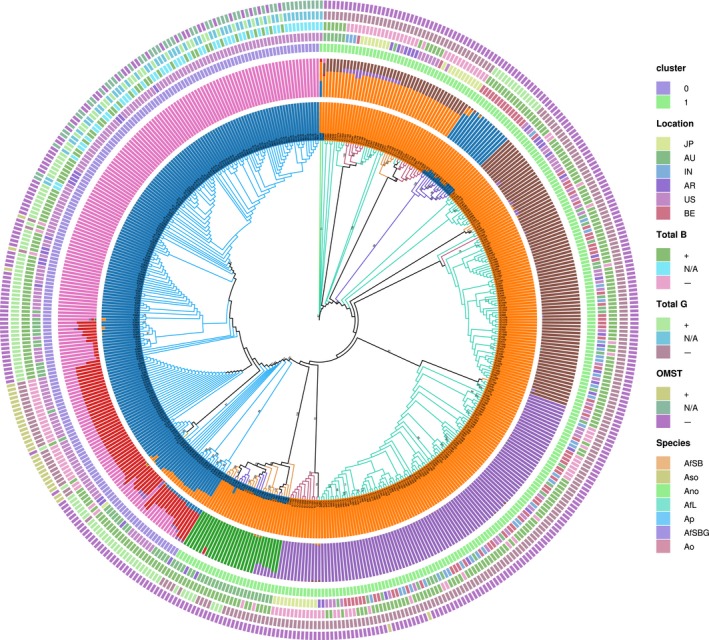
Graphical representation of the results from Structure analysis using the Evanno and Structure (LnP) methods, maximum‐likelihood phylogenetic analysis and principal component analysis (PCA). The circle tree represents the global phylogeny for six combined genomic regions (*aflM/aflN*,* aflW/aflX*,* amdS*,* trpC*,* mfs*, and *MAT*) for *A. flavus* (AfL, AfSB, AfSBG), *A. nomius* (Ano), *A. oryzae* (Ao), *A. parasiticus* (Ap), and *A. sojae* (Aso) populations. The innermost rings show the inferred clusters, using the Evanno method (*k* = 2; first ring) followed by the LnP method (*k* = 7; second ring), and the gap statistic (*k* = 2; third ring). The six geographical localities (JP, AU, IN, AR, US, and BE) are shown in the fourth ring. The three outermost rings show whether isolates are producing (+) or not producing (−) B aflatoxin (fifth ring), G aflatoxin (sixth ring) and OMST (seventh ring); N/A is for data not available

**Figure 2:**
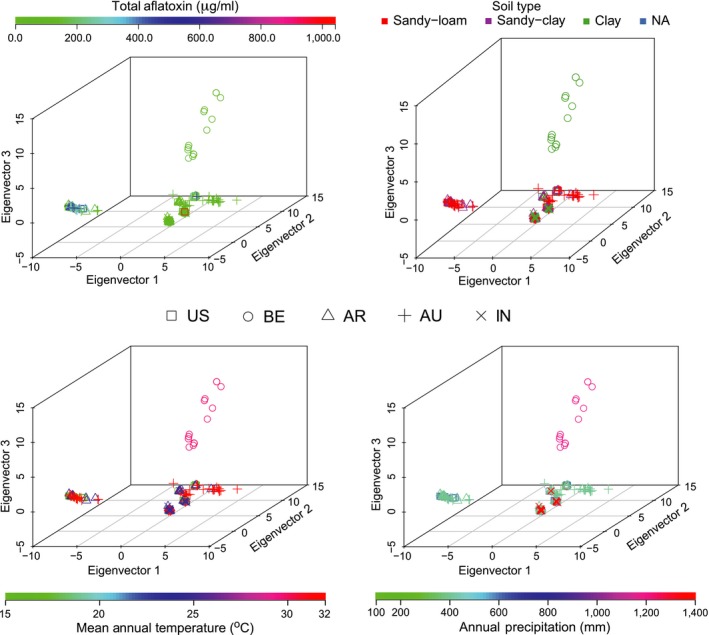
Principal component analysis (PCA) scatter plots for the global combined populations of *A. flavus* and *A. parasiticus* that represent associations based on four attributes: total aflatoxins, soil type, annual temperature, and annual precipitation. Each scatter plot's color scheme is unique to the particular attribute examined, and the different shapes relate to the geographic origin of each isolate

On a global scale, all geographic localities sampled showed some degree of genetic cohesion indicative of shared evolutionary history. This was evident by the sharing of haplotypes among strains from distant localities across six combined genomic loci (Figure 1). There was little genetic differentiation based on geography (fourth ring; Figure 1), except for a single clade of S_BG_ from Benin (Figures 1 and 2). Lineage IB was represented in this analysis, but on a global scale it also included toxigenic *A. flavus* strains such as IC445 and IC457 from Argentina, as well as IC640, IC642, and IC672 from Australia. Also, several haplotypes and clades (Figure 1) were associated with specific chemotype profiles for *A. parasiticus* (e.g., B‐ or G‐dominance, OMST production). Structure analysis, using the Evanno method (first innermost ring; Figure 1) and the gap statistic (third ring), inferred two major genetic clusters that corresponded with *A. parasiticus* (blue‐colored branches) and *A. flavus sensu lato* (predominantly green‐colored branches); species boundaries were well defined, with only a small percentage (~5%) of isolates showing admixture (Table 3). Seven distinct clusters were inferred using the STRUCTURE LnP method (second innermost ring) and separated isolates belonging to different species/lineages and showing differences in mycotoxin production. The three outermost rings correspond to production of B aflatoxins (fifth ring), G aflatoxins (sixth ring), and OMST (seventh ring), respectively. Each individual was assigned a membership coefficient in the cluster, with coefficients summing to 1 across the seven clusters. Of the three toxin chemotype categories, G aflatoxin production was more strongly correlated (*r* ^2 ^= .7220; *p *< .001; residual standard error: 0.2471) with the seven clusters than was B aflatoxin (*r* ^2 ^= .2685; *p *<* *.001; residual standard error: 0.3801) or OMST (*r* ^2 ^= .3363; *p *<* *.001; residual standard error: 0.221). About 72% of the variance found in G aflatoxin production can be explained by a strain's membership into one of the seven clusters, whereas only 33% of the variation (*F*_ST _= 0.3335, *p *<* *.001) was explained by variation between localities. This was also supported in PCA scatter plots in Figure 2; with the exception of a subset of isolates sampled in Benin, there was tight clustering of isolates from different localities.

**Table 3:** Isolates showing inferred admixture between genetic clusters of *A. parasiticus* and *A. flavus*, based on Structure analysis using the Evanno method in Figure 1

Isolate	Species	Locality	Total B (μg/ml)	Total G (μg/ml)
IC73	*A. parasiticus*	USA	76	399.7
IC157^a^	*A. nomius*	USA	+	+
IC328	*A. parasiticus*	USA	9	93.2
IC329	*A. parasiticus*	USA	23.2	245
IC330	*A. parasiticus*	USA	20.4	230.2
IC331	*A. parasiticus*	USA	31	314
IC490	*A. parasiticus*	Argentina	23.4	45.5
IC517	*A. parasiticus*	Argentina	37.8	160
IC642^b^	*A. flavus* L	Australia	165.1	0
IC477	*A. flavus* S_BG_	Argentina	2.3	6.2
IC478	*A. flavus* S_BG_	Argentina	2	7.2
IC494	*A. parasiticus*	Argentina	68.4	221.2
IC526	*A. parasiticus*	Argentina	0.2	1.1
IC720	*A. flavus* S_BG_	Australia	4.6	11.3
IC723	*A. flavus* S_B_	Australia	77.1	0
IC731	*A. flavus* S_BG_	Australia	1	1.3
IC733	*A. flavus* S_BG_	Australia	1.3	2
IC735	*A. flavus* S_BG_	Australia	1	1.6
IC741	*A. flavus* S_B_	Australia	68.6	0
IC742	*A. flavus* S_BG_	Australia	3.2	5.9
IC743	*A. flavus* S_B_	Australia	23.9	0
IC744	*A. flavus* S_BG_	Australia	8	17.6
IC751	*A. flavus* S_B_	Australia	15	0
IC753	*A. flavus* S_B_	Australia	34.4	0
IC755	*A. flavus* S_B_	Australia	10	0
IC758	*A. flavus* S_B_	Australia	6.9	0
IC760	*A. flavus* S_B_	Australia	79	0
IC768	*A. flavus* S_B_	Australia	14	0
IC770	*A. flavus* S_B_	Australia	58.1	0
IC777	*A. flavus* S_B_	Australia	12.9	0
IC780	*A. flavus* S_B_	Australia	77.5	0
IC785	*A. flavus* S_B_	Australia	34.9	0
IC786	*A. flavus* S_B_	Australia	32	0
IC787	*A. flavus* S_B_	Australia	27.7	0
IC788	*A. flavus* S_BG_	Australia	0.4	0.3
IC790	*A. flavus* S_B_	Australia	16.7	0
IC791	*A. flavus* S_B_	Australia	13.9	0
IC792	*A. flavus* S_B_	Australia	73.7	0
IC793	*A. flavus* S_B_	Australia	0	0
IC798	*A. flavus* S_B_	Australia	14.3	0
IC806	*A. parasiticus*	Australia	53.5	278.1
IC811	*A. parasiticus*	Australia	21.2	65.5
IC813	*A. parasiticus*	Australia	19.1	52.2
IC832	*A. parasiticus*	Australia	383.2	8.6
IC836	*A. parasiticus*	Australia	22.1	60.5
IC837	*A. parasiticus*	Australia	45.8	127.7
IC839	*A. parasiticus*	Australia	107.4	223.9
IC851	*A. parasiticus*	Australia	135.1	215.9
IC860	*A. parasiticus*	Australia	15.3	44.3
IC863	*A. parasiticus*	Australia	49.7	127.7
IC867	*A. parasiticus*	Australia	115.5	92.2
IC875	*A. parasiticus*	Australia	230.7	202.7
IC876	*A. parasiticus*	Australia	17.4	53.5
IC906	*A. parasiticus*	USA	37	222.5
IC907	*A. parasiticus*	USA	65.7	420.5
IC911	*A. parasiticus*	USA	16.5	172.8
IC920	*A. parasiticus*	USA	84.7	361
IC1112	*A. flavus* S_BG_	Benin	35.4	312.6
IC1113	*A. flavus* S_BG_	Benin	11	13.7
IC1117	*A. flavus* S_BG_	Benin	13.2	26.1
IC1118	*A. flavus* S_BG_	Benin	14.4	18
IC1119	*A. flavus* S_BG_	Benin	13.4	18.8
IC1133	*A. flavus* S_BG_	Benin	11.8	14.6
IC1134	*A. flavus* S_BG_	Benin	12.6	15.8
IC1140	*A. flavus* S_BG_	Benin	4.8	18
IC1141	*A. flavus* S_BG_	Benin	12.9	16.8
IC1142	*A. flavus* S_BG_	Benin	47.1	117.7
IC1144	*A. flavus* S_BG_	Benin	15.1	30.8
IC1145	*A. flavus* S_BG_	Benin	15.8	12.5
IC1147	*A. flavus* S_BG_	Benin	14.1	9.2
IC1149	*A. flavus* S_BG_	Benin	18.9	40.6
IC1150	*A. flavus* S_BG_	Benin	21.6	48.1
IC1215^a^	*A. sojae*	Japan	–	–
IC1258	*A. flavus* L	India	10.7	0

a No aflatoxin quantification data acquired. Strain is designated aflatoxin positive (+) or negative (−).

b Isolate produces G_1 _< 0.5 μg/ml.

A single most parsimonious tree for each locus is illustrated in Figs [Link], [Link], [Link], [Link]. Haplotype designations for each phylogeny are found in supplemental Tables [Link], [Link], [Link], [Link], [Link], [Link], [Link]. Phylogenies inferred for noncluster loci *amdS* and *trpC* show well‐supported clades that are associated with species or morphotype. Shared haplotypes between species from different geographic regions suggest recent descent from a common ancestor prior to geographic isolation, or alternatively, extensive migration between localities. For *amdS* (Fig. S1; S10), there is strong bootstrap support (>75%) for many nodes; however, clades are not monophyletic and not associated with a single geographic region. For example, haplotype H12 includes a mix of *A. alliaceus*,* A. caelatus*,* A. flavus* L and S, *A. oryzae* and *A. tamarii* isolates from different localities. Although there was no evidence of geographic structure, some haplotypes based on species or morphotype, such as *A. flavus* S haplotypes H48‐H53, were associated only with Argentina and Australia and included a mix of AFB or AFB+G producers. With the exception of an Australian *A. flavus* S_BG_ isolate (H5) and an Australian *A. flavus* L isolate (H42), global *A. parasiticus* is monophyletic.

Isolates associated with Geiser's Groups I and II are dispersed throughout the *amdS* phylogeny in haplotypes H12, H20, and H25, and haplotypes H9, H49, and H50, respectively. Haplotype H12 includes multiple species, while H20 and H25 include only *A. flavus* L strains. Haplotype H20 includes lineage IC isolates such as the AF36 biocontrol strain and NRRL 3357, while H12 encompasses isolates from both IC and IB lineages. Multiple geographic origins are represented in each haplotype. Although all of our Geiser's Group II isolates were of the S morphotype, their chemotype profiles were a mixture of AFB and AFB+G. All but one of the isolates, IC477, represented in Group II haplotypes are from Australia. Isolate IC477 (H49) is an AFB+G strain from Argentina, whereas haplotype H9 is solely comprised of S_B_ stains.

The *trpC* phylogeny (Fig.S1; Table S11) supports the monophyly of species in *Aspergillus* section *Flavi* (Peterson et al., 2008). Four main clades were observed: *A. flavus*,* A. parasiticus*,* A. caelatus* with *A. tamarii*, and *A. nomius*. Though there is evidence of shared haplotypes within the *A. flavus* and *A. parasiticus* clades (haplotypes H9 and H30, respectively); overall, species groupings are upheld. The *trpC* Geiser Group I haplotypes are spread throughout the *A. flavus* clade and includes isolates of *A. flavus* L and S, *A. oryzae* and *A. caelatus*. Isolates associated with Geiser's Group II were found in only a single haplotype (H15), and are comprised of *A. flavus* S‐strains from Australia and Argentina, with AFB and AFB+G chemotypes.

Although highly divergent when compared to each other, the *MAT1‐1* and *MAT1‐2* idiomorphs are highly conserved on the DNA and amino acid levels when examined separately, both within and between species (Ramirez‐Prado et al., 2008). This was not the case for the *MAT* loci in *A. nomius* (Fig. S2; Tables S12 and S13), which showed elevated haplotype diversity; there were five haplotypes inferred for 15 *MAT1‐1* isolates and fourteen haplotypes for 25 *MAT1‐2* isolates. Moreover, *A. nomius* harbors both *MAT* idiomorphs within a single genome for seven of the 32 (22%) sampled isolates. Nine of the 22 haplotypes (41%) for the *MAT1‐1* phylogeny, and 15 of the 30 haplotypes (50%) for the *MAT1‐2* phylogeny include *A. nomius* isolates. Both mating‐type phylogenies show evidence of trans‐speciation, and there is little evidence of clades that are structured geographically. An Australian *A. alliaceus* isolate (IC892) appears most divergent from other sampled species for both *MAT1‐1* (H4) and *MAT1‐2* (H17). This isolate may be homothallic since it has both *MAT* loci, whereas Australian *A. allicaeus* isolates IC888 (H7; *MAT1‐1*) and IC894 (H21; *MAT1‐2*) appear to have only one *MAT* locus present in their genomes and are likely heterothallic.

The *mfs* phylogeny (Fig. S2; Table S14) shows three major clades with strong bootstrap support: *A. parasiticus* (top), *A. caelatus* (middle), and *A. flavus* (bottom). This is concordant with the species tree inferred from the *trpC* locus. Although clades show extensive geographic mixing, haplotypes H43‐H46 representing a subset of Benin *A. flavus* S_BG_ strains are well differentiated from the rest of the global sample. The only two S_B_ isolates sampled in Benin share haplotypes (H15 and H31) with *A. flavus* L strains. A second well‐supported group of *A. flavus* S isolates includes haplotypes H23, H24, and H26, which are a mixture of S_B_ and S_BG_ from more than one locality. Haplotype H2 includes an Argentinian *A. caelatus* isolate among global *A. parasiticus* individuals. This same *A. caelatus* isolate (IC568) grouped with a haplotype that includes mostly *A. flavus* for *amdS* (H12 in Fig. S1; Table S10). There was some evidence of chemotype/morphotype admixture with an Australian S_BG_ isolate sharing haplotype H15 with global L and S_B_ strains. Other *mfs* haplotypes such as H2, H17, and H36 may harbor chemotype‐specific differences that are maintained via trans‐speciation.

Phylogenetic inference for the cluster locus, *aflM/aflN* (Fig. S3; Table  S15), indicates that species are not as monophyletic as observed in some of the noncluster loci. Haplotype groupings do exist that show geographic and species differentiation such as *A. flavus* S_BG_ from Benin (H55, H58‐H61). Although not well supported, this clade of S_BG_ from Benin shares a recent common ancestor with another group of predominantly S_B_ isolates from different localities (H2‐H8) and includes isolates that associate with Geiser's Group II. Isolate IC793 (H6) is nonaflatoxigenic which is considered rare for *A. flavus* S‐strains (Horn & Dorner, 1999). Other haplotypes contain isolates that associate with Geiser's Group I (H10 and H39) and Group II (H78). There is also evidence of trans‐speciation in *aflM/aflN*, which has been reported previously (Carbone et al., 2007). For example, in haplotype H39, an *A. parasiticus* isolate from Argentina groups with global *A. flavus* isolates. Interestingly, haplotype H47 includes the AF36 biocontrol strain, and despite being sampled from different geographic regions (Arizona, USA; Texas, USA; Karnataka, India), all four of the nonaflatoxigenic strains in this haplotype have the same nonsense mutation in *aflC* (data not shown), which suggests dispersal that transcends geographic boundaries as reported in other studies (Grubisha & Cotty, 2015; Ortega‐Beltran, Grubisha, Callicott, & Cotty, 2016). Moreover, many nonaflatoxigenic *A. flavus* L isolates, from various localities, group with *A. oryzae*. More than 90% of these nonaflatoxigenic isolates belong to Lineage IB (Moore et al., 2009). Nestled among these haplotypes was a group of B‐ and G‐dominant *A. parasiticus* isolates (H31‐H36) from various localities.

The *aflW/aflX* locus (Fig. S4; Table S16) appears more concordant with the noncluster loci in that there is evidence of grouping based on species and/or geography. There are clades that are predominantly *A. flavus* L, *A. parasiticus*,* A. flavus* S, and a backbone of *A. oryzae* that separates most of the sample from a second *A. flavus* clade, which includes Lineage IB isolates. Many of the haplotypes for this region of the aflatoxin cluster show evidence of trans‐speciation (Table S16) such that some isolates of nonaflatoxigenic *A. caelatus*,* A. tamarii*,* A. oryzae* and *A. alliaceus* are distributed across haplotypes shared with *A. flavus* (H20, H29, H34, H36, H40, H41, H48, H50) or *A. parasiticus* (H5, H8). Eleven of the 55 haplotypes harbor trans‐specific polymorphisms. Haplotypes encompassing isolates that associate with Geiser's Group I are H40, H50 and H53, while haplotypes H19‐H22 include isolates that associate with Group II. The single‐locus RAxML analysis of the *aflW/aflX* region (Fig. S5) shows that clades are predominantly lineage‐specific rather than species‐ or geography‐specific. This is illustrated through the mixed branch colors in the tree. The branches that include Lineage IB isolates are labeled along the tree's perimeter and include species other than *A. flavus*. For example, *A. caelatus*,* A. nomius* and *A. oryzae* are each split into two separate lineages. PCA for the *aflW/aflX* region (Fig. S5) for the global sample suggests seven distinct clusters that do not align with species or geography designations. The Lineage IB cluster is encircled and is not entirely comprised of nonaflatoxigenic isolates. For the *amdS* and *trpC* loci (Fig. S5), the RAxML phylogenies are concordant with the species tree. These species are not as clearly delimited in the PCA scatter plots of *amdS* and *trpC* for which *k*‐values indicate the presence of six and seven distinct clusters, respectively. There was limited resolution of species boundaries in the *MAT1‐1* mating‐type gene in both the maximum‐likelihood phylogeny and PCA scatter plots (Fig. S6). The *k*‐value of four includes one cluster of *A. alliaceus* from Australia, a disperse cluster containing *A. nomius* and *A. tamarii* from the USA and Benin, a cluster containing *A. caelatus* from the USA and Argentina, and a fourth cluster containing a mixture of species from various geographic localities. Similarly for the *MAT1‐2* mating gene (Fig. S7), the maximum‐likelihood tree and PCA scatter plots show limited resolution of species. The PCA results only reveal two unique clusters, grouping a genetically distinct strain of *A. alliaceus* from Australia with an outlier strain of *A. tamarii* from India; the remaining isolates comprise the second cluster.

Multilocus maximum‐likelihood tree and PCA scatter plot comparisons of *A. flavus* and *A. parasiticus* populations within each geographic region can be seen in Figs [Link], [Link], [Link], [Link], [Link]. For the US population (Fig. S8), some clustering is observed based on species/morphotype and chemotype profile. In each PCA scatter plot, a *k*‐value of seven indicates there is an additional cluster being masked by one of the six observed clusters. Some of these clusters are species‐specific, but not necessarily field‐specific (top scatter plot) or chemotype‐specific (bottom scatter plot). Figure S9 illustrates the maximum‐likelihood tree and PCA scatter plot results for the Argentina populations. Species/morphotype delimitation is more easily discernible than chemotype grouping in the maximum‐likelihood phylogeny as well as in the PCA scatter plots that have a *k*‐value of three. The first principal component (eigenvector 1) indicated that the *A. flavus* L and S_B_ isolates share a cluster, with S_BG_ and *A. parasiticus* isolates comprising the second and third clusters, respectively. Based on the second principal component (eigenvector 2), three possible clusters are discernible, except the *A*. *flavus* L cluster is subdivided and a portion of them are loosely clustered with the S_B_, S_BG_ and *A. parasiticus* strains. In Fig. S10, there is evidence of strong species/morphotype structure within the Australian maximum‐likelihood phylogeny, although there is also evidence of admixture of different chemotypes in each clade. The PCA for the Australian populations of *A. flavus* and *A. parasiticus* in this figure suggests a *k*‐value of two and indicates a degree of similarity among *A. flavus* morphotypes (L‐ and S‐strains) that loosely groups the two morphotypes while separating them from *A. parasiticus*. The second principal component (eigenvector 2) for this group of isolates indicates subdivision in *A. flavus* L, in which some isolates group more closely with S_B_, S_BG_ and *A. parasiticus*. The Benin ML tree mainly shows clades associated with chemotype profile (Fig. S11). This is supported by PCA and a *k*‐value of six despite all of the isolates belonging to *A. flavus*. Most apparent is the subdivision of *A. flavus* S_BG_ isolates into multiple groupings. Two of the clusters are each a mixture of *A. flavus* L and S_B_ isolates; the other four clusters are comprised of one or more *A. flavus* S_BG_ isolates. The ML tree for the India *A. flavus* population (Fig. S12) is only represented by a single morphotype (L‐strains), and clades are associated with chemotype profiles being either aflatoxin positive or negative. PCA scatter plots indicate many distinct groupings of isolates, but there is not enough variation for reliable estimation of *k* value.

## 4 DISCUSSION

This study focused on species in *Aspergillus* section *Flavi* that were present in peanut fields at the time of sampling (2004–2006). Although other species have since been described (e.g., *A. minisclerotigenes*,* A. arachidicola*,* A. mottae*; see Pildain et al., 2008; Soares, Rodrigues, Peterson, & Venâncio, 2012), these were not sampled from the peanut fields examined in this study, nor did comprehensive BLASTn searches of NCBI databases reveal close matches to these other species. Our inferences were therefore limited to species that were sampled in peanut fields. The results from this expanded global sample of species in *Aspergillus* section *Flavi* confirms and strengthens inferences of population processes in the aflatoxin gene cluster of these agriculturally important species. First, we see widespread evidence of balancing selection acting on G_1_‐ and B_1_‐dominant *A. parasiticus* chemotypes in both the *aflM/aflN* and *aflW/aflX* regions. Previously this was detected only in the *aflM/aflN (hypE)* locus for the Georgia *A. parasiticus* population (Carbone et al., 2007). Similarly, there is global evidence for Lineage IB (predominantly nonaflatoxigenic) in *A. flavus* that was first inferred from this same Georgia field (Horn & Greene, 1995; Moore et al., 2009). It appears that these patterns of trans‐speciation for specific chemotype profiles transcend not only species but also geographic boundaries. Seven distinct genetic clusters were more strongly structured by G chemotype diversity than geography. Although there can be other factors that underlie these seven clusters, chemotype‐specific evolutionary lineages were observed in previous studies (Carbone et al., 2007; Moore et al., 2009). Whether these chemotype‐specific lineages have increased pathogenicity or enhanced fitness for some other trait or ecological condition is unknown and warrants further study.

Climate has been reported to influence toxin production and population size of *A. flavus* S in the USA (Bock, Mackey, & Cotty, 2004; Cotty & Jaime‐Garcia, 2007). Although reports of *A. flavus* S‐strains in the USA producing G aflatoxins (S_BG_) are rare, they are common elsewhere (Cotty & Cardwell, 1999). For example, the Benin population we examined included S_BG_ isolates; however, no *A. parasiticus* isolates were sampled in our Benin field. With regard to populations of aflatoxin‐producing fungi, species that produce both B and G aflatoxins such as *A. parasiticus* and *A. nomius* are seldom found in certain localities (Cotty & Cardwell, 1999). Given that recombination in fungi can occur during times of high stress (Meng et al., 2015), one possibility is that *A. parasiticus* found its way out of an inhospitable soil environment, through hybridization with more adaptable A*. flavus*. However, attempting to prove this hypothesis would require more extensive sampling and analyses. The observation that *A. flavus* S_BG_ isolates from Benin, which may or may not be related to the unnamed taxon S_BG_ isolated from Nigerian groundnut in the 1960s (Probst, Callicott, & Cotty, 2012), are genetically distinct from other *A. flavus* populations sampled could indicate that this subpopulation is under strong selection and possibly evolving into its own distinct lineage. PCA analysis suggests that there could be further splitting of the Benin *A. flavus* S_BG_ isolates into multiple groups (Figure 2), each associated with a specific chemotype profile; this is unknown and warrants further investigation. Egel, Cotty, and Elias (1994) reported that *A. flavus* S_B_ are closely related to the *A. flavus* L‐type strain based on molecular variation, suggesting a possible common ancestor between S_B_ and *A. flavus* L. The high sequence diversity observed within the *MAT* locus among *A. nomius* isolates sampled in the USA could relate to multiple *A. nomius* lineages as reported by Ehrlich et al. (2007) among isolates from Thailand.

Collectively, molecular sequence data in cluster and noncluster loci indicate a potential hybrid origin for some strains. For example, analysis of the *amdS* locus showed Australian S_BG_ isolate IC721 sharing a haplotype with *A. flavus* L and S_B_ isolates, while another Australian S_BG_ isolate, IC744, shared a haplotype with *A. parasiticus* isolates based on the *mfs* locus. As the other loci examined were distinct in these S_BG_ strains it is plausible that the *mfs* locus was inherited from one or the other parent species during an interspecific recombination event. Alternatively, the similarity between *A. flavus* and *A. parasiticus* could be the result of balancing selection, which has been reported in the aflatoxin gene cluster (Carbone et al., 2007). Although *mfs* is adjacent to the aflatoxin cluster, it does not show the level of variation observed in cluster genes (Moore et al., 2009). The apparent similarity between these two species at the *mfs* locus may be the result of balancing selection acting on cluster genes that sweeps variation out of adjacent linked regions (Charlesworth, 2006).

Considering the chemotype profiles of the sampled Australian S_BG_ strains, 12 of the 26 examined strains also produce measurable quantities of *O*‐methylsterigmatocystin (OMST). This toxic secondary metabolite is an intermediate in aflatoxin synthesis, that is, often secreted by nonaflatoxigenic *A. parasiticus* strains (Bhatnagar et al., 1987) and further hints at possible interspecific hybridization among the *A. flavus* and *A. parasiticus* populations in Australia. The accumulation of OMST and its precursor sterigmatocystin (ST) has been reported in *Aspergillus* and allied fungi (Rank et al., 2011). While these and other secondary metabolites have been useful for species delimitation (Frisvad, Andersen, & Thrane, 2008) the mechanisms generating new chemotypes are less clear. Olarte et al. (2015) showed that *A. flavus* and *A. parasiticus*, when crossed in the laboratory, yielded offspring that exhibited chemotypes representative of both parent species, such as aflatoxin (*A. flavus* parent) and OMST production (*A. parasiticus* parent). The PCA scatter plots for Argentina (Fig. S9) and Australia (Fig. S10) show some proximity of S_BG_ isolates to a subset of the *A. flavus* L, S_B_ and *A. parasiticus* populations along the second principal component (eigenvector 2), which could be the result of interspecific recombination, a hypothesis which warrants further investigation. The discrepancy in the number of sampled S_BG_ strains between these two localities relates to differences in population sizes as seen in their mating‐type distributions. The Argentina populations are disproportionately clonal and dominated by mating‐type *MAT1‐1,* while the Australian populations are predominantly sexual and have an approximately 1:1 distribution of *MAT1‐1* and *MAT1‐2* (Moore et al., 2013).

Our results indicate the existence of genetic exchange and recombination between distinct morphotypes (*A. flavus* L, *A. flavus* S_B_, *A. flavus* S_BG_), evolutionary lineages (IB and IC) and species (e.g., *A. flavus* and *A. parasiticus*). For example, it has been suggested that the AF36 biocontrol strain is a recombinant that resulted from the hybridization of parents representing both *A. flavus* L and S morphotypes (Chang et al., 2012). We observed multiple isolates, from different localities, that share the same nonsense mutation found in the *aflC* locus of AF36; however, we have not determined whether they are recombinants in their aflatoxin clusters. Multilocus genotyping revealed toxigenic strains that group with Lineage IB strains. These strains could be recombinant offspring between Lineages IB and IC that have genomic signatures of IB, and have inherited aflatoxigenicity. Further sampling and examining more genomic regions are necessary to understand the origins of toxigenic stains in Lineage IB. Some of the aflatoxigenic isolates that share the signature of Lineage IB in their *aflW* locus produce high concentrations of aflatoxin. This “super‐producer” toxin phenotype might result when the nonaflatoxigenic phenotype is repaired via crossover recombination or independent assortment. One of these high‐producing strains, IC642 from Australia, is an *A. flavus* L‐strain that shares a haplotype with *A. parasiticus* isolates. This isolate, as well as other aflatoxigenic strains from Australia and Argentina, may have resulted from interspecific hybridization between *A. flavus* and *A. parasiticus*. The possibility of intra‐ and inter‐specific genetic exchange as driving forces for adaptive evolution and speciation in *Aspergillus* section *Flavi* merits further investigation.

## 5 CONCLUSIONS

There is widespread evidence of balancing selection acting on the presence or absence of aflatoxin‐specific chemotypes, which can be useful in delimiting species and population boundaries in *Aspergillus* section *Flavi*. For example, *A. flavus* S_BG_ strains in Benin are genetically and chemotypically distinct from *A. flavus* S_B_ strains in the US. A signature of trans‐speciation was observed in *A. flavus* L‐strains with the maintenance of nontoxigenic and toxigenic strains that belong to Lineages IB and IC, respectively. The predominance of *A. oryzae* in lineage IB supports the distinctiveness of these two evolutionary lineages. Previously we provided evidence of balancing selection acting on G_1_‐dominant strains in *A. parasiticus* (Carbone et al., 2007). We now show that balancing selection on G production is a major driver of species diversification in section *Flavi*. While recombination could explain differences in chemotype profiles, clonality and environmental conditions could also be important in maintaining diversity. A better understanding of this genotype by chemotype by environment interactions has implications not only in species delimitation but also in biological control using nonaflatoxigenic strains of *A. flavus*.

## CONFLICT OF INTEREST

None declared.

## AUTHOR CONTRIBUTIONS

GGM performed laboratory bench work, PCR and sequencing of genomic loci, clean‐up and preparation of genomic sequences and performed population analyses, drafted the manuscript, and created tables and figures for publication. RAO assisted with population analyses, assisted with manuscript drafting, and addressing reviewer concerns. BWH acquired, verified the species identities of, and shared fungal isolates, and served as a proof‐reader for the manuscript. JLE assisted with the laboratory bench work, as well as PCR and sequencing of genomic loci. RS assisted with the laboratory bench work, PCR and sequencing of genomic loci. CJO assisted with the laboratory bench work, PCR and sequencing of genomic loci. IC helped develop the population analysis software, assisted with population analyses, helped draft the manuscript and address reviewer concerns.

## DATA ACCESSIBILITY

DNA Sequences: GanBank accessions DQ390825‐DQ391160; FJ871513‐FJ878506; KX853136‐KX853999; HM353299‐HM355372; HM745598‐HM745901; HQ000095‐HM002855.
