# Supplementary figures and images for: Global population structure and adaptive evolution of aflatoxin‐producing fungi
Source: Ecol Evol. 2017 Sep 30;7(21):9179–91. doi: 10.1002/ece3.3464 (PMC5677503; doi:10.1002/ece3.3464)

***amdS***  
135 MP Trees  
CI = 0.6519

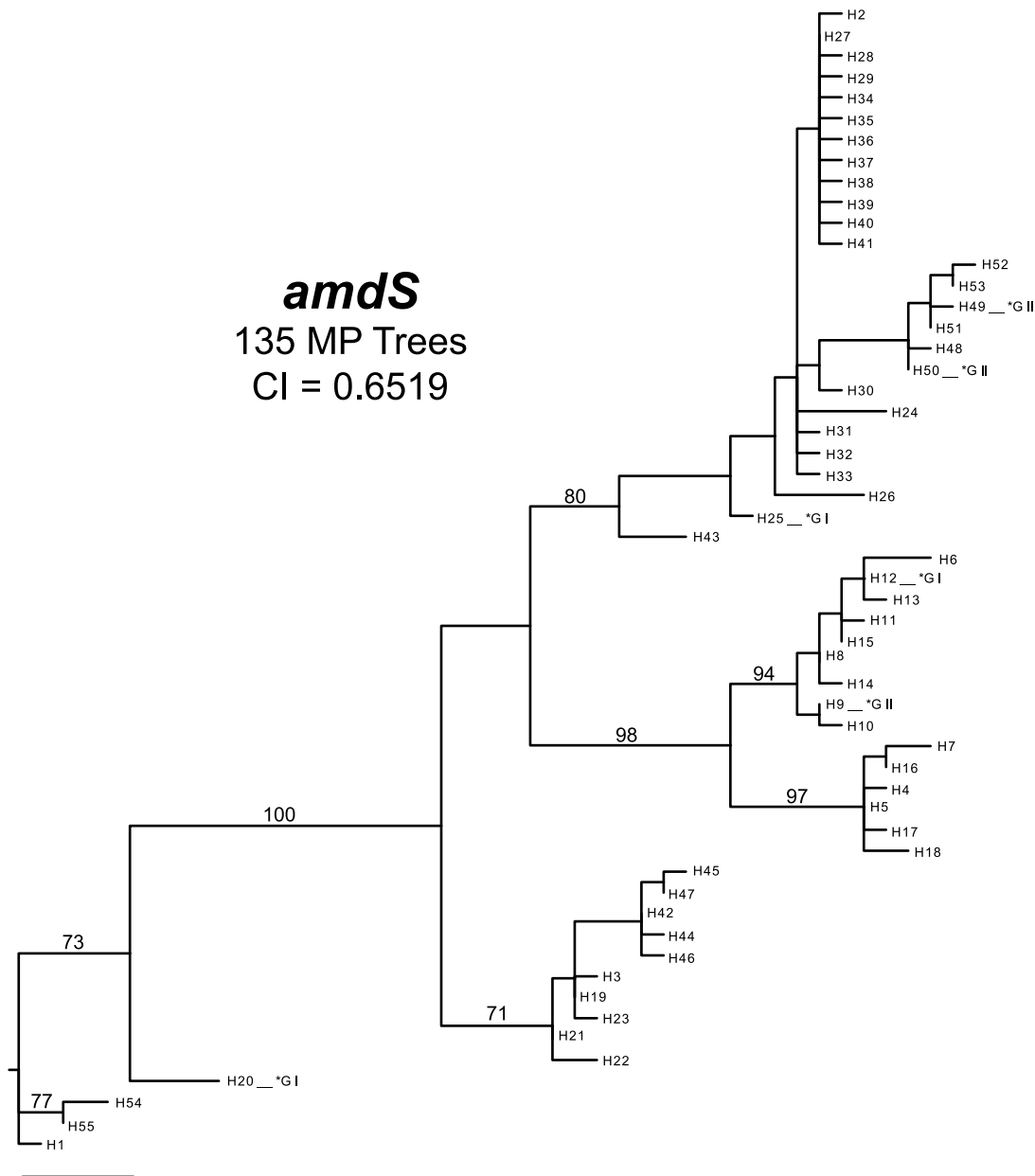

***trpC***  
20352 MP Trees  
CI = 0.8450

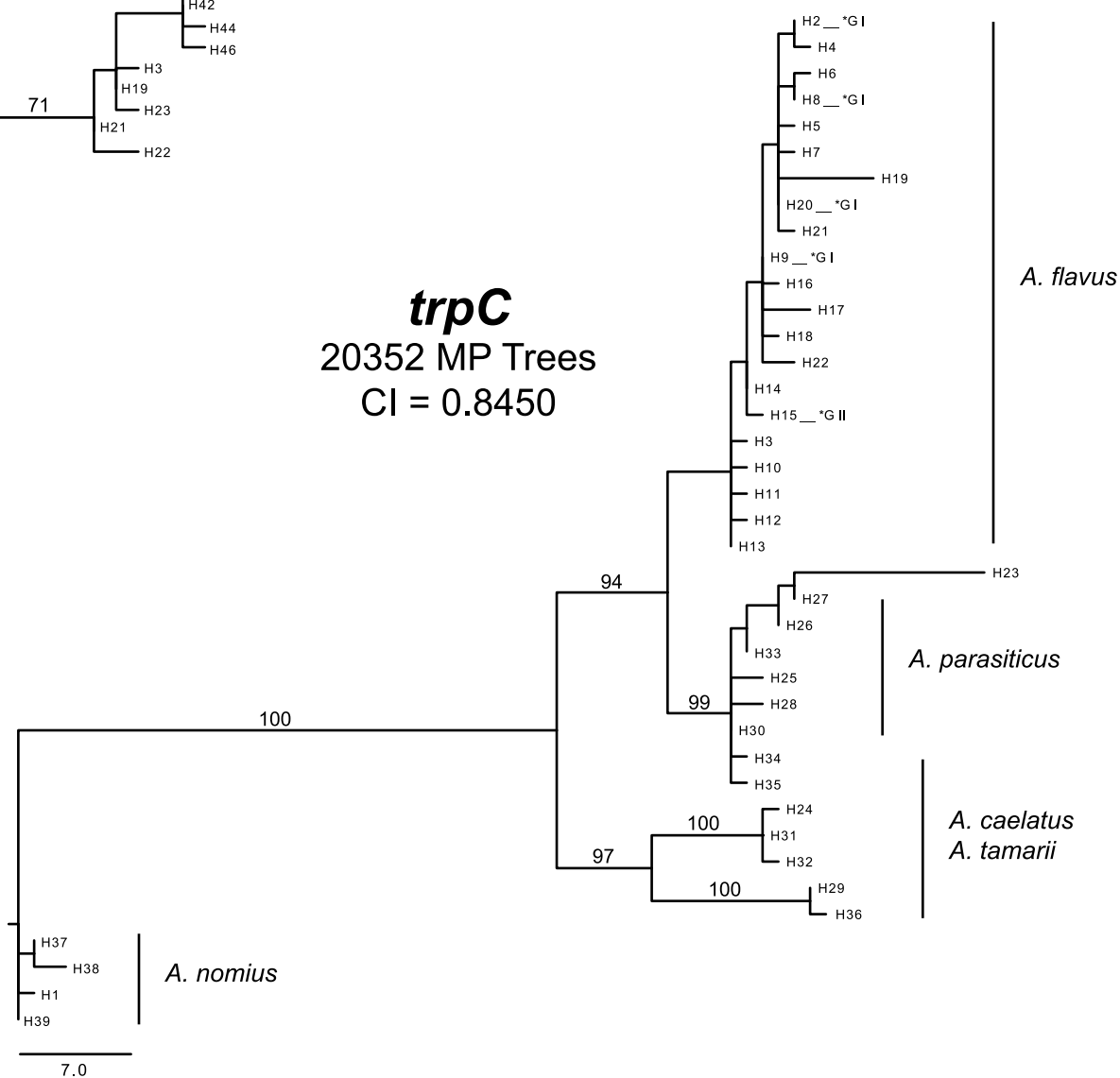

Supplement: Supplementary file 1 [file ECE3-7-9179-s001.pdf]

***MAT1-1***  
21 MP Trees  
CI = 0.8182

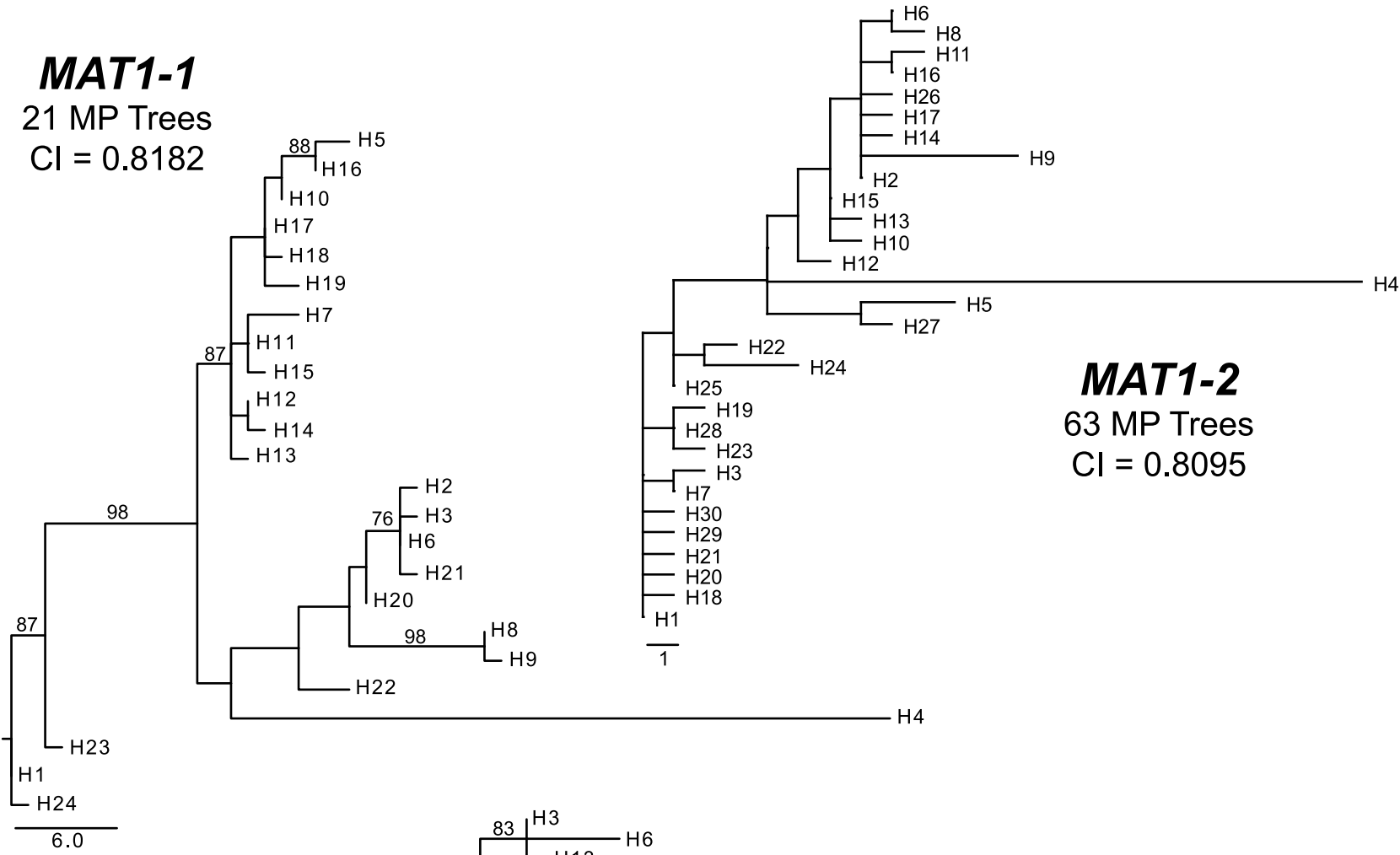

***MAT1-2***  
63 MP Trees  
CI = 0.8095

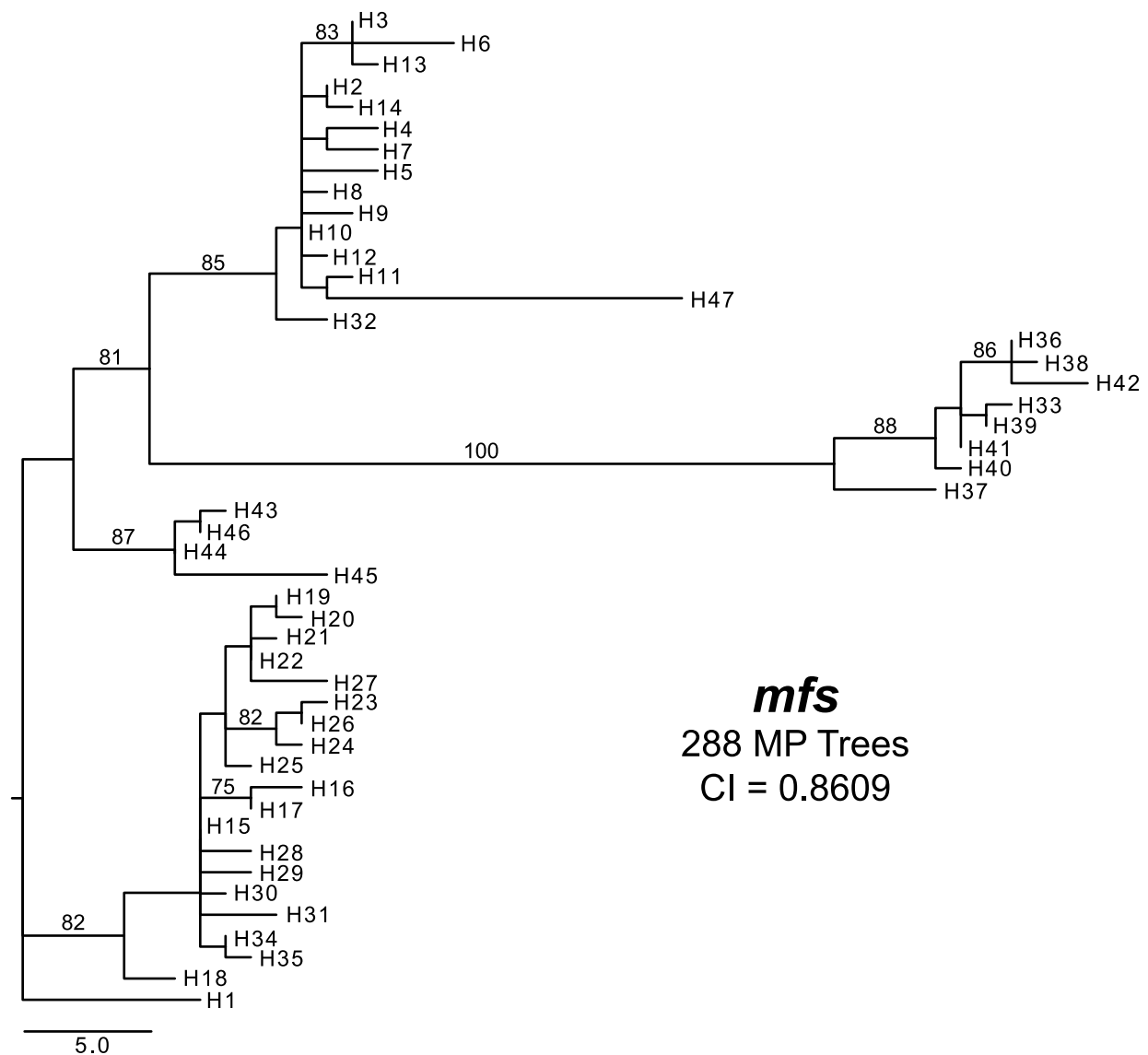

***mfs***  
288 MP Trees  
CI = 0.8609

Supplement: Supplementary file 2 [file ECE3-7-9179-s002.pdf]

***afIM/afIN***

1438 MP Trees

CI = 0.6440

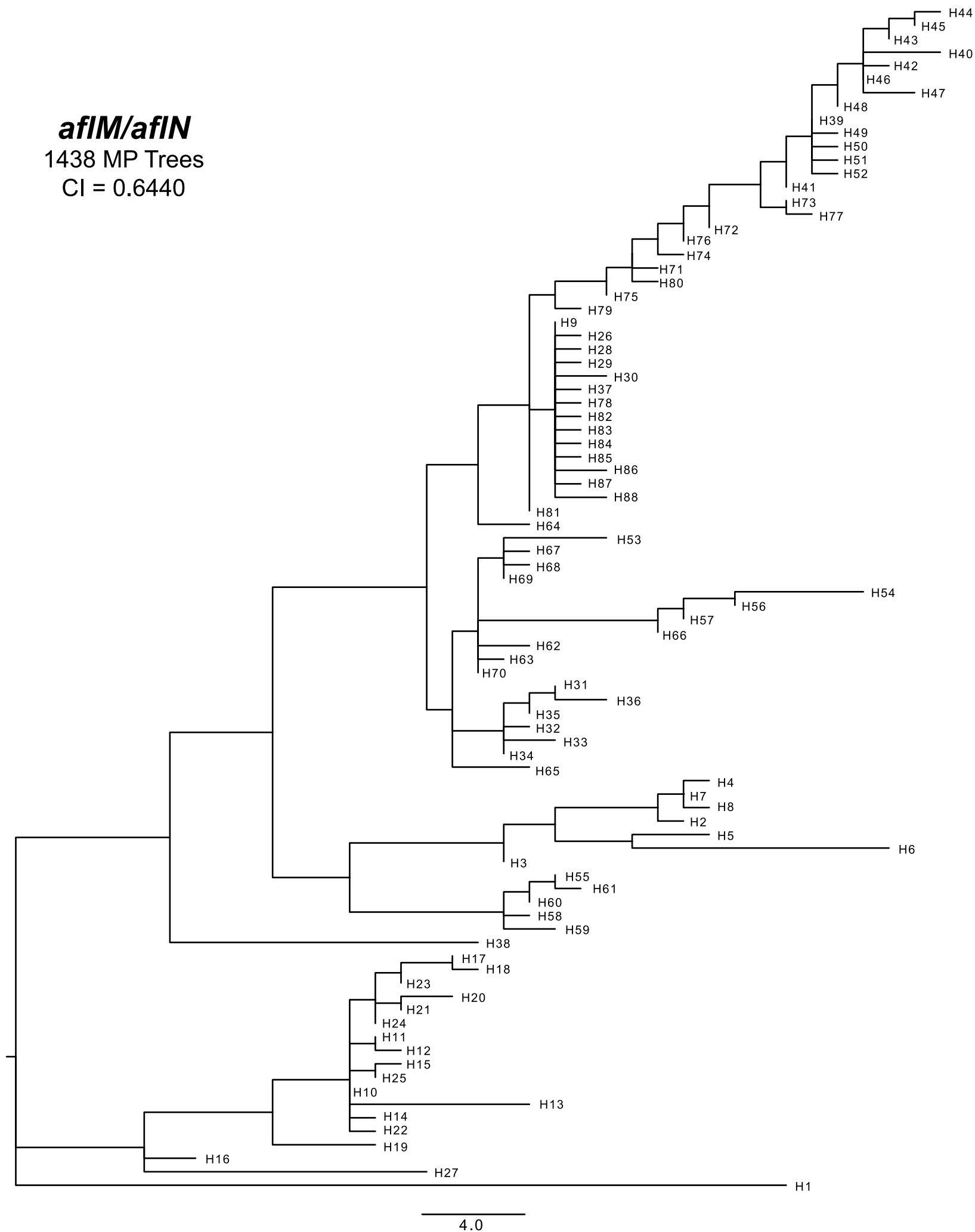

Supplement: Supplementary file 3 [file ECE3-7-9179-s003.pdf]

***aflW/aflX***

1062 MP Trees

CI = 0.6608

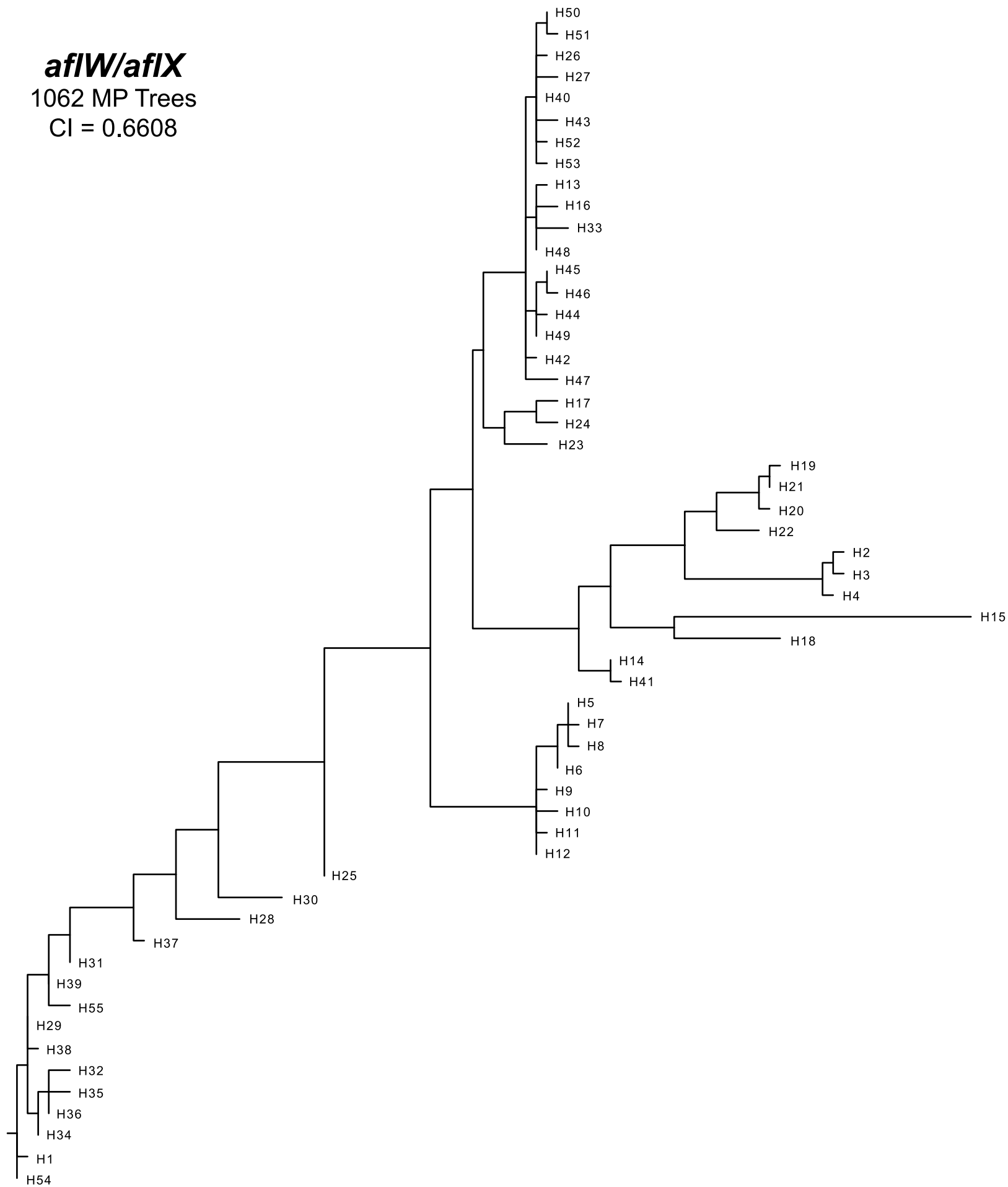

10.0

Supplement: Supplementary file 4 [file ECE3-7-9179-s004.pdf]

# Maximum Likelihood Phylogeny

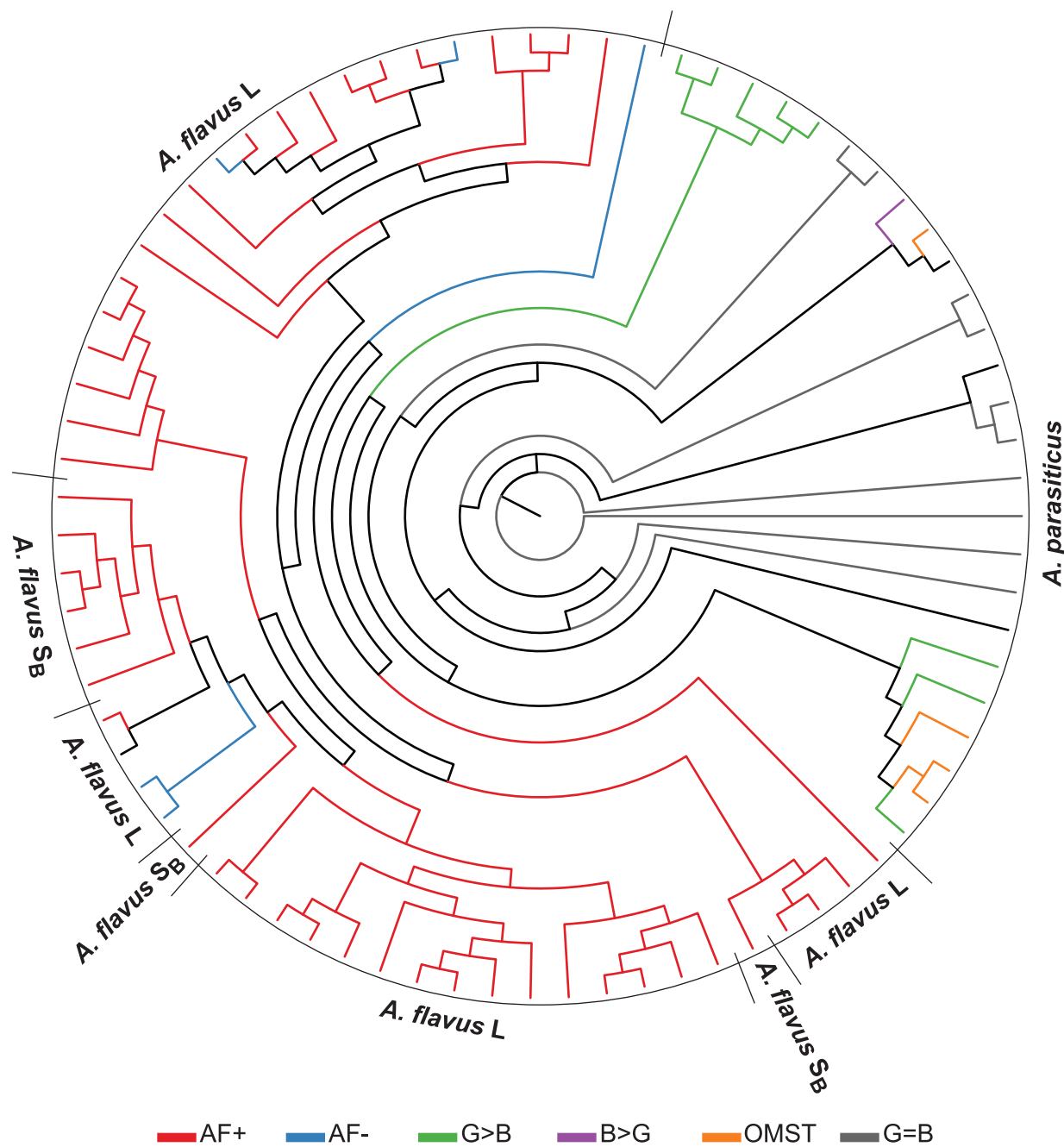

# Principal Component Analysis

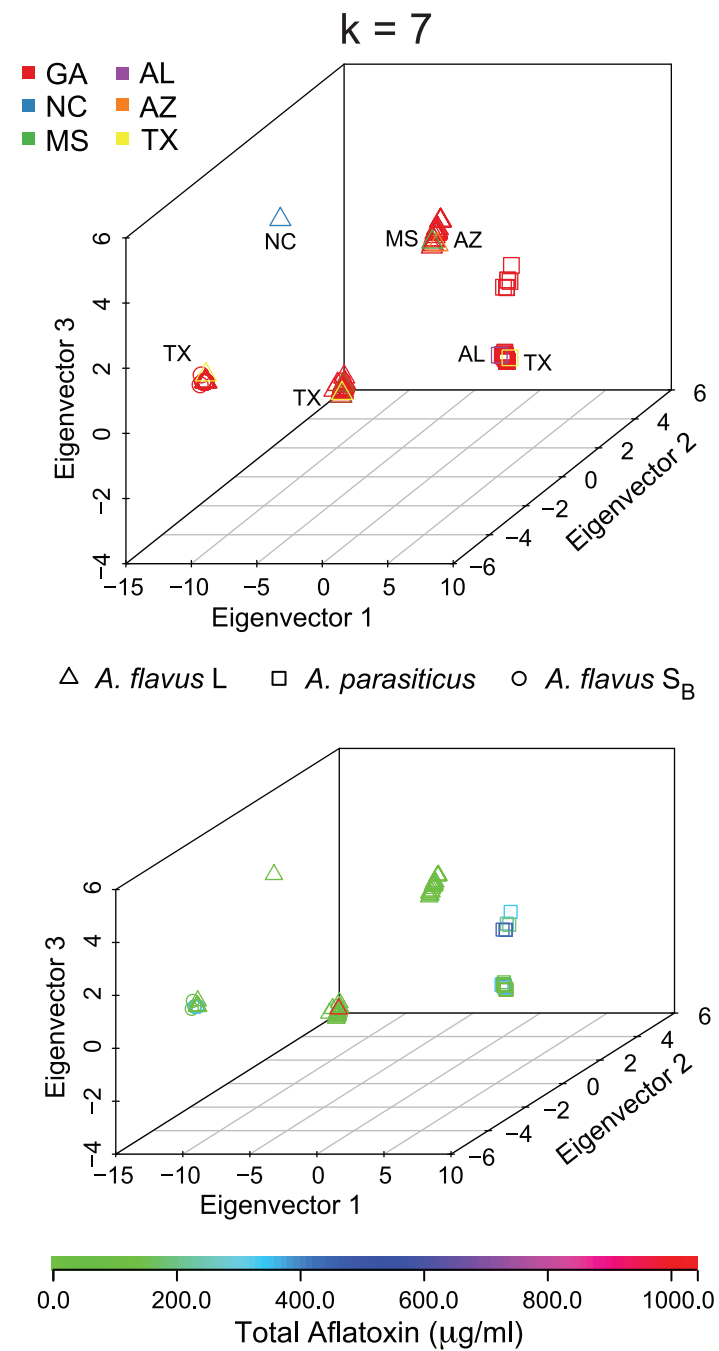

Supplement: Supplementary file 8 [file ECE3-7-9179-s008.pdf]

# Maximum Likelihood Phylogeny

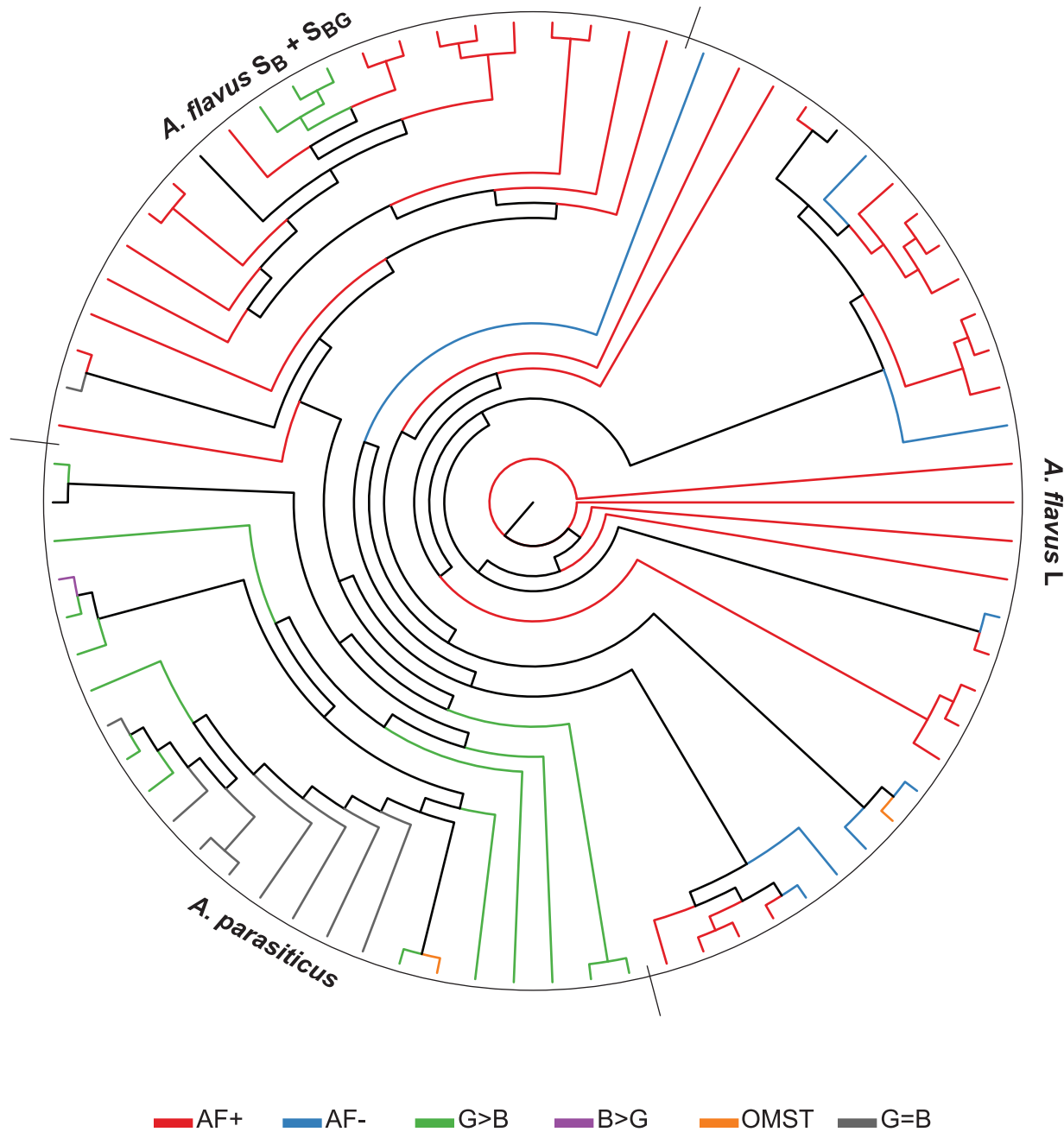

# Principal Component Analysis

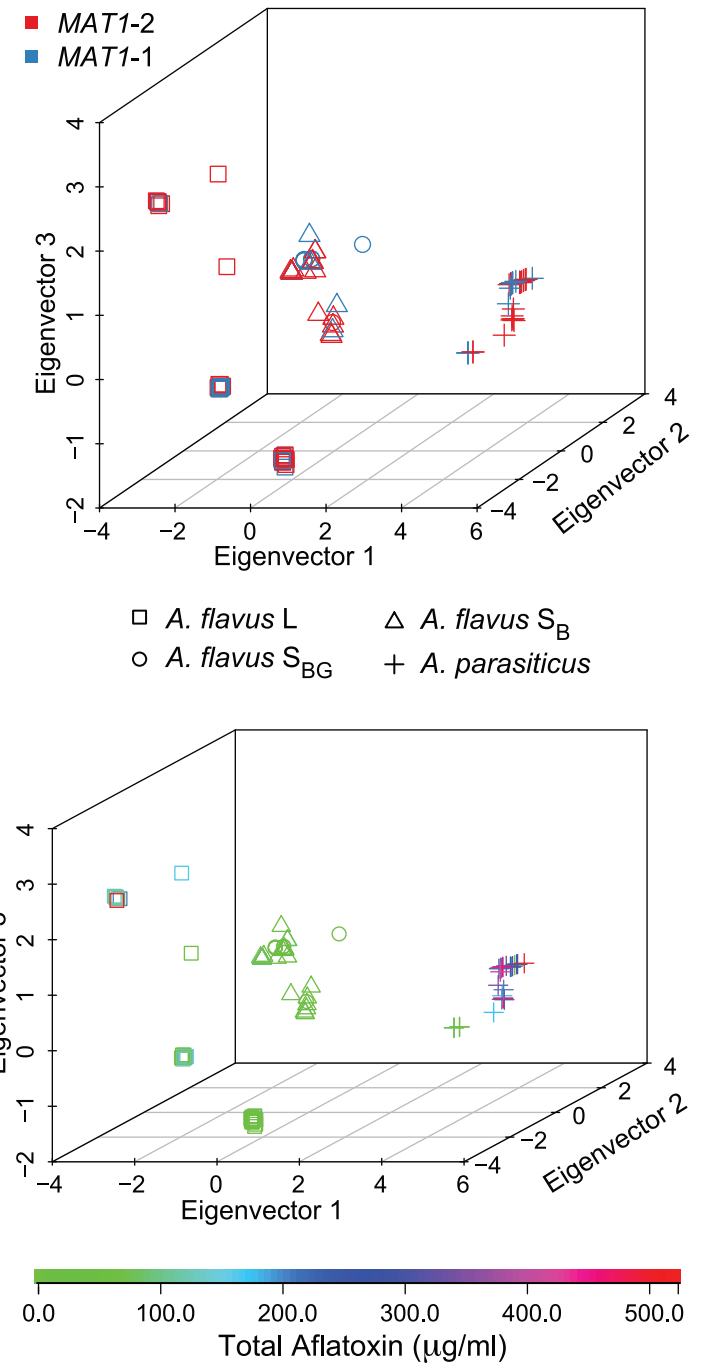

Supplement: Supplementary file 10 [file ECE3-7-9179-s010.pdf]

Maximum Likelihood Phylogeny

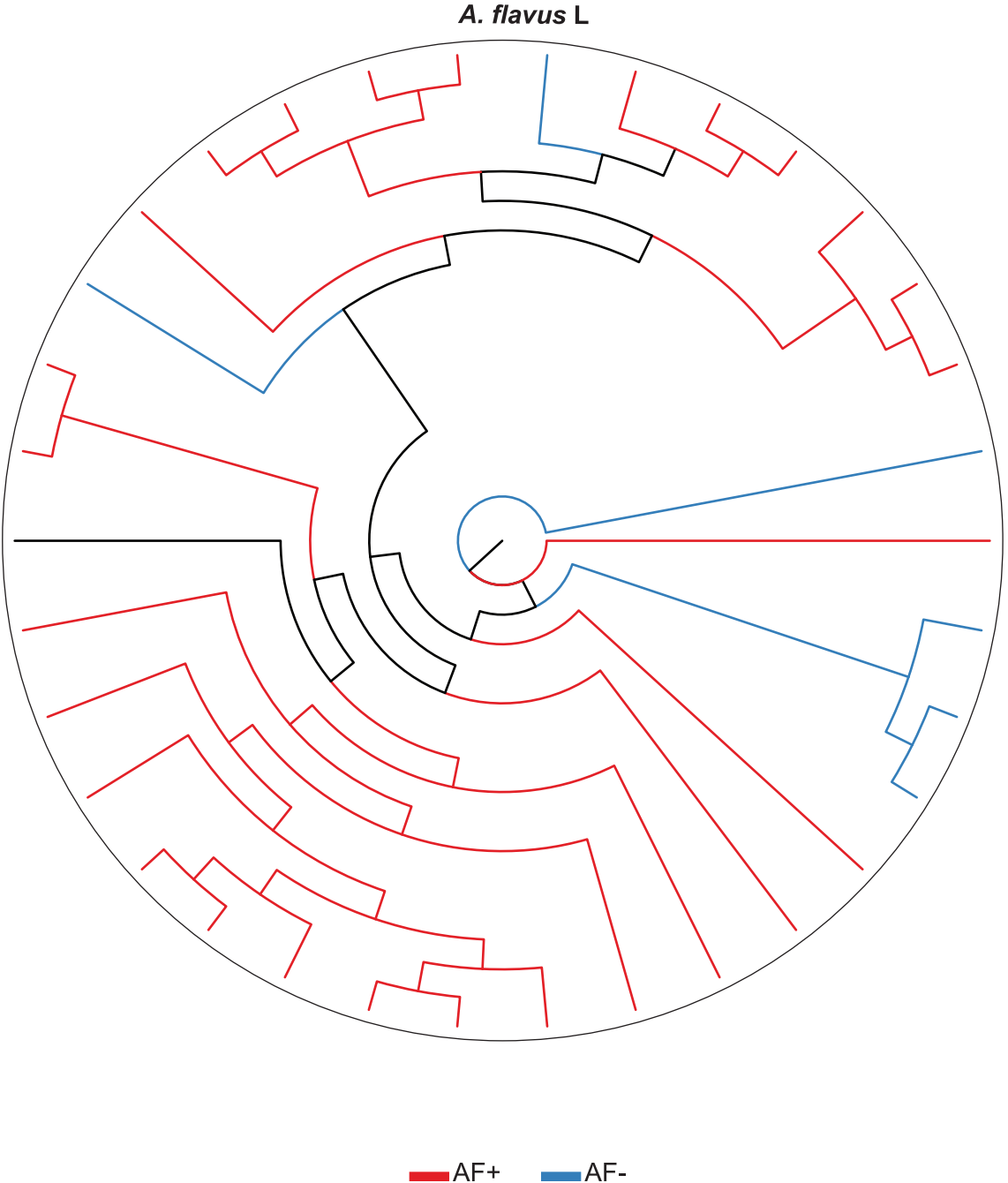

Principal Component Analysis

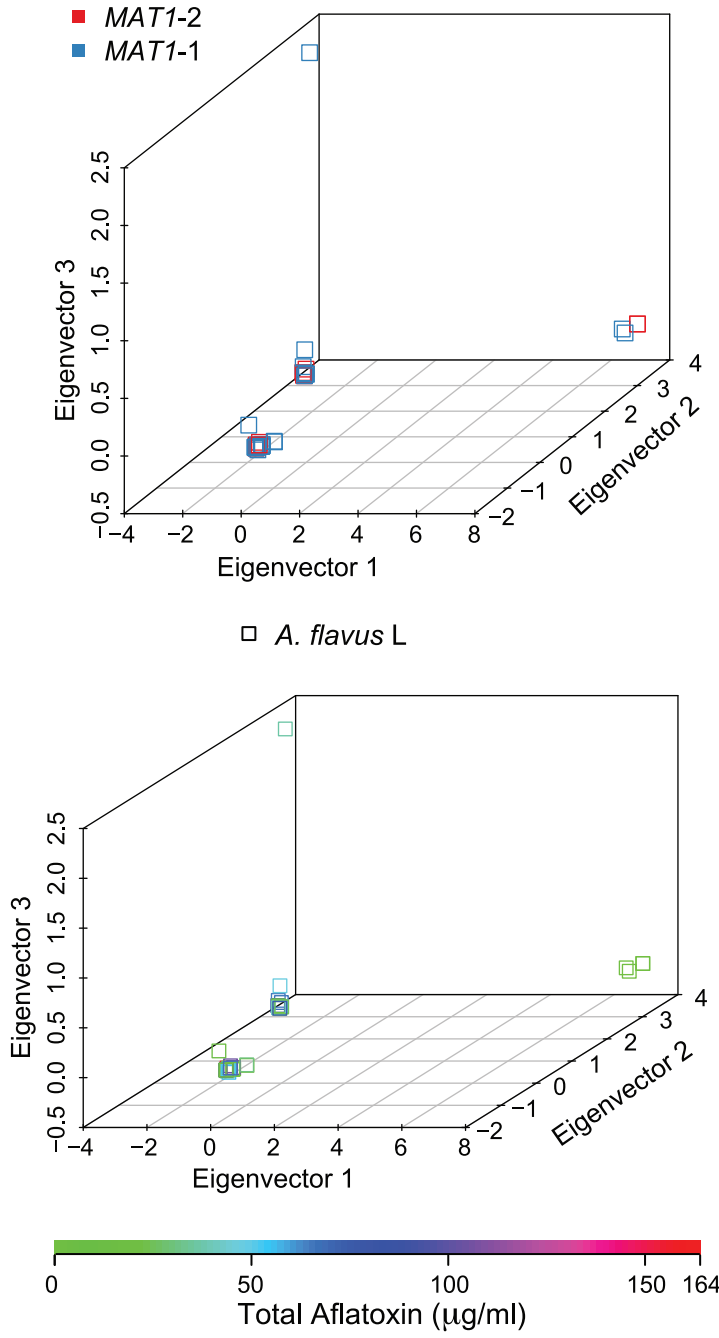

Supplement: Supplementary file 12 [file ECE3-7-9179-s012.pdf]
