# Supplementary material for: Global population structure and adaptive evolution of aflatoxin‐producing fungi
Source: Ecol Evol. 2017 Sep 30;7(21):9179–91. doi: 10.1002/ece3.3464 (PMC5677503; doi:10.1002/ece3.3464)
Supplement: Supplementary file 5 [file ECE3-7-9179-s005.pdf]

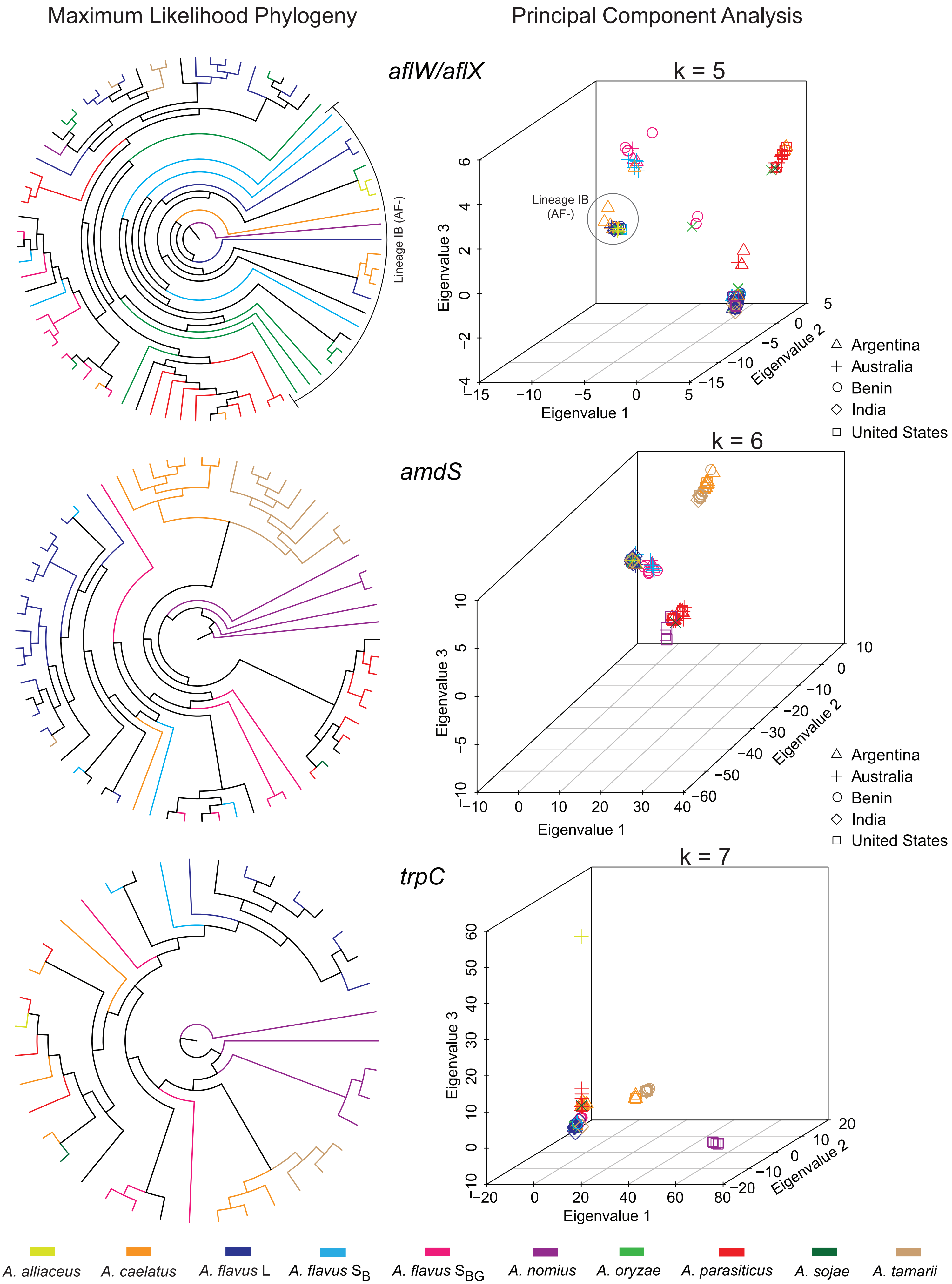

Maximum Likelihood Phylogeny
Principal Component Analysis
aflW/aflX
k = 5
Lineage IB (AF-)
Eigenvalue 1
Eigenvalue 2
Eigenvalue 3
Argentina
Australia
Benin
India
United States
amdS
k = 6
trpC
k = 7
A. alliaceus
A. caelatus
A. flavus L
A. flavus S$_B$
A. flavus S$_{BG}$
A. nomius
A. oryzae
A. parasiticus
A. sojae
A. tamarii
