# Supplementary material for: Global population structure and adaptive evolution of aflatoxin‐producing fungi
Source: Ecol Evol. 2017 Sep 30;7(21):9179–91. doi: 10.1002/ece3.3464 (PMC5677503; doi:10.1002/ece3.3464)
Supplement: Supplementary file 6 [file ECE3-7-9179-s006.pdf]

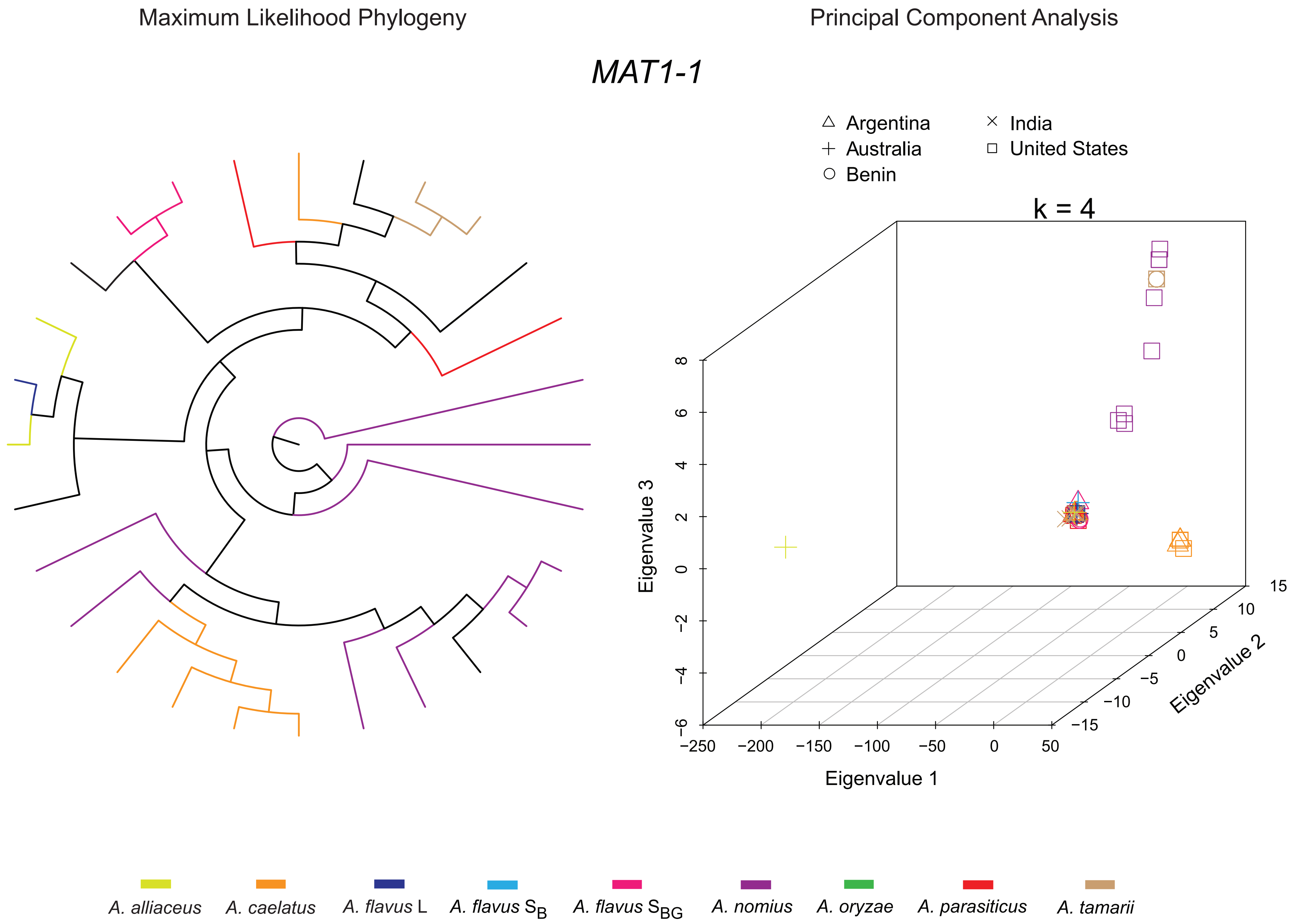
Maximum Likelihood Phylogeny
Principal Component Analysis
MAT1-1
Argentina
Australia
Benin
India
United States
k = 4
Eigenvalue 1
Eigenvalue 2
Eigenvalue 3
−250
−200
−150
−100
−50
0
50
−15
−10
−5
0
5
10
15
−6
−4
−2
0
2
4
6
8
A. alliaceus
A. caelatus
A. flavus L
A. flavus S$_B$
A. flavus S$_{BG}$
A. nomius
A. oryzae
A. parasiticus
A. tamarii
