# Supplementary material for: Global population structure and adaptive evolution of aflatoxin‐producing fungi
Source: Ecol Evol. 2017 Sep 30;7(21):9179–91. doi: 10.1002/ece3.3464 (PMC5677503; doi:10.1002/ece3.3464)
Supplement: Supplementary file 7 [file ECE3-7-9179-s007.pdf]

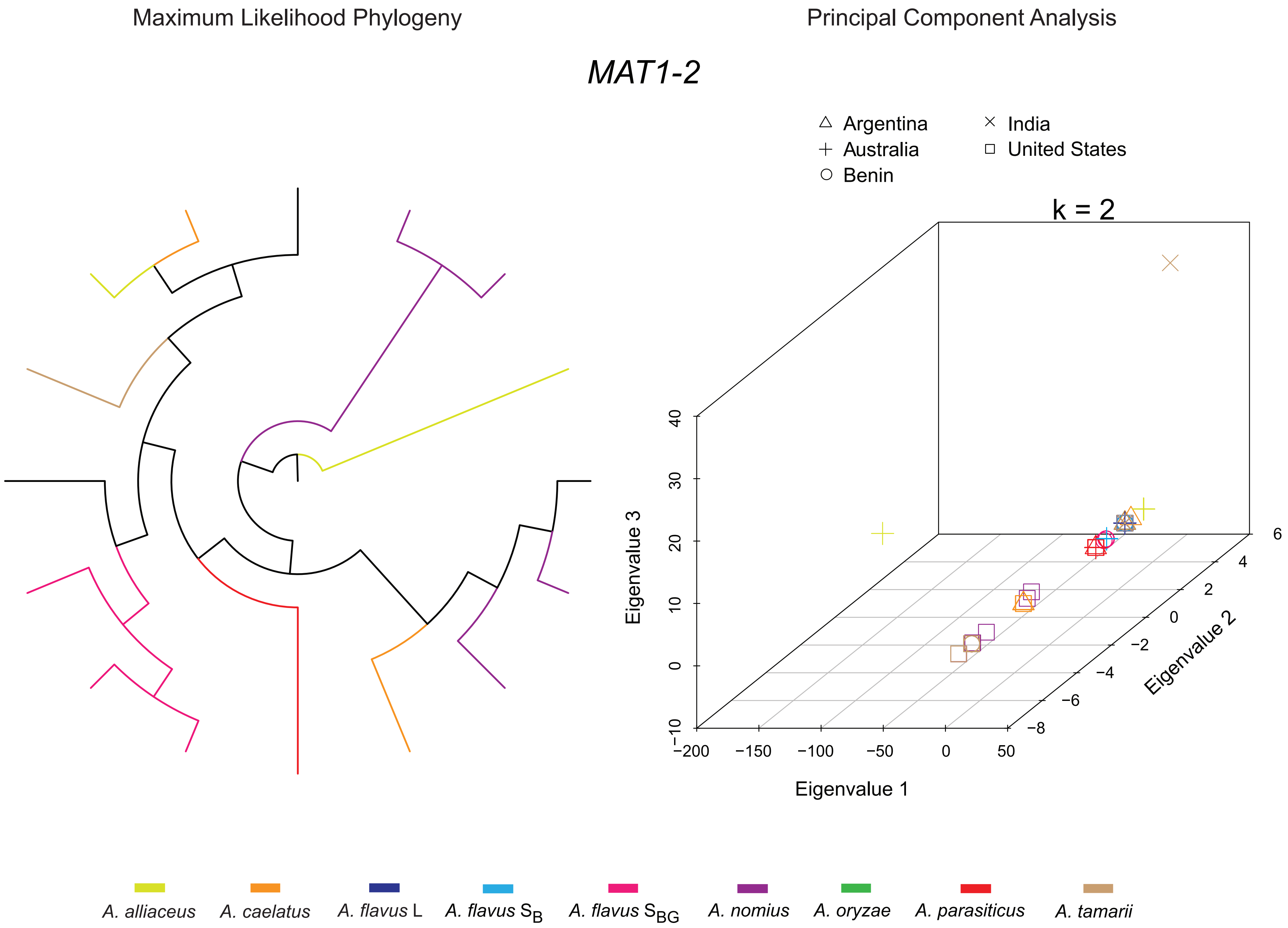

Maximum Likelihood Phylogeny
Principal Component Analysis
MAT1-2
Argentina
Australia
Benin
India
United States
k = 2
Eigenvalue 1
Eigenvalue 2
Eigenvalue 3
−200
−150
−100
−50
0
50
−10
0
10
20
30
40
−8
−6
−4
−2
0
2
4
6
A. alliaceus
A. caelatus
A. flavus L
A. flavus S_B
A. flavus S_BG
A. nomius
A. oryzae
A. parasiticus
A. tamarii
