# Supplementary material for: Global population structure and adaptive evolution of aflatoxin‐producing fungi
Source: Ecol Evol. 2017 Sep 30;7(21):9179–91. doi: 10.1002/ece3.3464 (PMC5677503; doi:10.1002/ece3.3464)
Supplement: Supplementary file 9 [file ECE3-7-9179-s009.pdf]

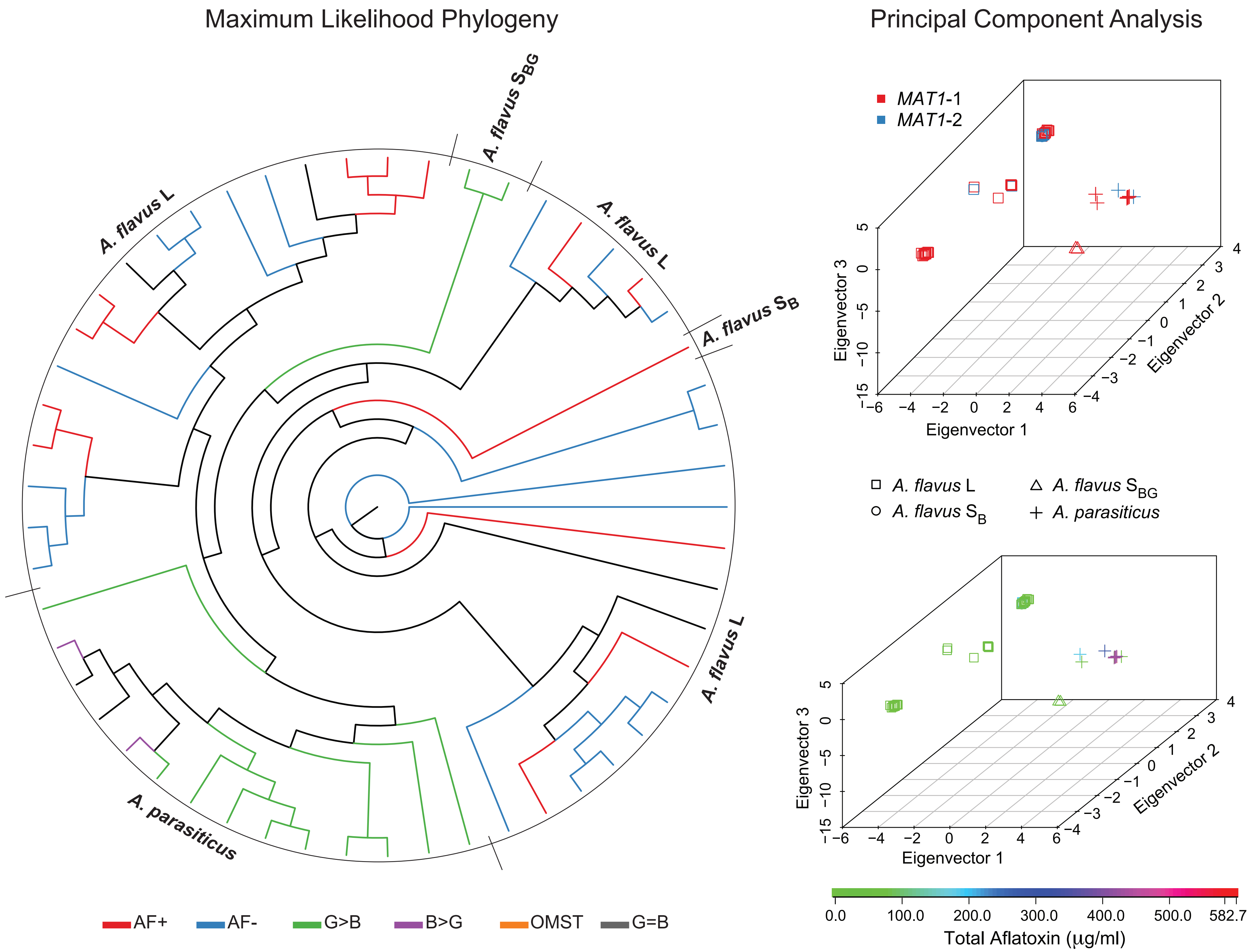

Maximum Likelihood Phylogeny
Principal Component Analysis
A. flavus L
A. flavus SBG
A. flavus L
A. flavus SB
A. flavus L
A. parasiticus
AF+
AF-
G>B
B>G
OMST
G=B
MAT1-1
MAT1-2
Eigenvector 1
Eigenvector 2
Eigenvector 3
A. flavus L
A. flavus SB
A. flavus SBG
A. parasiticus
Total Aflatoxin (µg/ml)
0.0
100.0
200.0
300.0
400.0
500.0
582.7
