# Supplementary material for: Global population structure and adaptive evolution of aflatoxin‐producing fungi
Source: Ecol Evol. 2017 Sep 30;7(21):9179–91. doi: 10.1002/ece3.3464 (PMC5677503; doi:10.1002/ece3.3464)
Supplement: Supplementary file 13 [file ECE3-7-9179-s013.doc]

Table S1. Climate, soil type and species richness, in sampled peanut fields, from each geographic region

|  |  | Córdoba, Argentina | Queensland, Australia | Littoral, Benin | Karnataka, India | Georgia, USA^c^ |
| --- | --- | --- | --- | --- | --- | --- |
|  | Temperature (^o^C)^a^ | 32 | 32 | 32 | 32 | 30 |
|  | Precipitation (mm)^b^ | 700 | 700 | 1400 | 1400 | 700 |
|  | Soil Type | sandy clay | sandy loam | feralitic clay | alfisol/clay | sandy loam |
|  |  |  |  |  |  |  |
| *A. alliaceus* |  | 0 | 9 | 0 | 0 | 0 |
| *A. caelatus* |  | 80 | 0 | 0 | 0 | 31 |
| *A. flavus* L |  | 80 | 80 | 80 | 80 | 79 |
| *A. flavus* S |  | 4 | 80 | 44 | 0 | 25 |
| *A. nomius* |  | 0 | 0 | 0 | 0 | 32 |
| *A. oryzae* |  | 0 | 0 | 0 | 0 | 0 |
| *A. parasiticus* |  | 80 | 80 | 0 | 0 | 114 |
| *A. sojae* |  | 0 | 0 | 0 | 0 | 0 |
| *A. tamarii* |  | 0 | 6 | 80 | 56 | 33 |
|  |  |  |  |  |  |  |
| Total |  | 244 | 255 | 204 | 136 | 314 |

^a^ Approximated mean annual temperature averaged over 50 years. Moore, Elliott *et al*. 2013. PLoS Pathogens 9(8): e1003574.

^b^ Approximated mean annual precipitation averaged over 50 years. Moore, Elliott *et al*. 2013. PLoS Pathogens 9(8): e1003574.

^c^ Species counts for USA only include those sampled in Georgia field.
