# Supplementary material for: Global population structure and adaptive evolution of aflatoxin‐producing fungi
Source: Ecol Evol. 2017 Sep 30;7(21):9179–91. doi: 10.1002/ece3.3464 (PMC5677503; doi:10.1002/ece3.3464)
Supplement: Supplementary file 14 [file ECE3-7-9179-s014.docx]

Table S2. GenBank accession numbers for *A. flavus* L-strain sequences used in this study

| **IC Strain** | ***W/X*** | ***M/N*** | ***MAT*** | ***amdS*** | ***mfs*** | ***trpC*** |
| --- | --- | --- | --- | --- | --- | --- |
| 203 | HQ002472 | HQ002673 | HQ002263 | HQ000178 | HQ000632 | HQ001382 |
| 204 | HQ002470 | HQ002674 | HQ001714 | HQ000179 | HQ000633 | HQ001383 |
| 217 | HQ002465 | HQ002675 | HQ001715 | HQ000180 | HQ000634 | HQ001384 |
| 218 | HQ002459 | HQ002676 | HQ001716 | HQ000181 | HQ000635 | HQ001385 |
| 219 | HQ002458 | HQ002677 | HQ001717 | HQ000182 | HQ000636 | HQ001386 |
| 220 | HQ002457 | HQ002678 | HQ001718 | HQ000183 | HQ000637 | HQ001387 |
| 221 | HQ002462 | HQ002679 | HQ002264 | HQ000184 | HQ000638 | HQ001388 |
| 222 | HQ002461 | HQ002680 | HQ002265 | HQ000185 | HQ000639 | HQ001389 |
| 223 | HQ002460 | HQ002681 | HQ002266 | HQ000186 | HQ000640 | HQ001390 |
| 225 | HQ002466 | HQ002682 | HQ001719 | HQ000187 | HQ000641 | HQ001391 |
| 226 | HQ002475 | HQ002683 | HQ001720 | HQ000188 | HQ000642 | HQ001392 |
| 227 | HQ002474 | HQ002684 | HQ002267 | HQ000189 | HQ000643 | HQ001393 |
| 228 | HQ002469 | HQ002685 | HQ002268 | HQ000190 | HQ000644 | HQ001394 |
| 229 | FJ877446 | FJ878062 | HQ002269 | FJ877376 | FJ877231 | FJ877161 |
| 232 | HM355230 | HM353569 | HQ002270 | HM354289 | HQ000645 | HM354021 |
| 233 | HM355231 | HM353570 | HQ002271 | HM354290 | HQ000646 | HM354022 |
| 234 | FJ877449 | FJ878072 | HQ002272 | FJ877379 | FJ877234 | FJ877164 |
| 237 | HM355232 | HM353571 | HQ002273 | HM354291 | HQ000647 | HM354023 |
| 238 | HM355233 | HM353572 | HQ002274 | HM354292 | HQ000648 | HM354024 |
| 239 | HM355234 | HM353573 | HQ002275 | HM354293 | HQ000649 | HM354025 |
| 240 | HM355235 | HM353574 | HQ002276 | HM354294 | HQ000650 | HM354026 |
| 241 | HM355236 | HM353575 | HQ002277 | HM354295 | HQ000651 | HM354027 |
| 242 | HM355237 | HM353576 | HQ002278 | HM354296 | HQ000652 | HM354028 |
| 243 | HQ002464 | HQ002686 | HQ001721 | HQ000191 | HQ000653 | HQ001395 |
| 245 | FJ877453 | FJ878064 | HQ002279 | FJ877383 | FJ877238 | FJ877168 |
| 248 | HM355238 | HM353577 | HQ002280 | HM354297 | HQ000654 | HM354029 |
| 249 | HM355239 | HM353578 | HQ002281 | HM354298 | HQ000655 | HM354030 |
| 250 | HM355240 | HM353579 | HQ002282 | HM354299 | HQ000656 | HM354031 |
| 251 | HM355241 | HM353580 | HQ002283 | HM354300 | HQ000657 | HM354032 |
| 252 |  |  | HQ002284 | FJ877385 | FJ877241 | FJ877170 |
| 253 | HQ002463 | HQ002687 | HQ002285 | HQ000192 | FJ877242 | HQ001396 |
| 254 | HQ002473 |  | HQ002286 | HQ000193 | FJ877243 | FJ877172 |
| 255 |  |  | HQ002287 | HQ000194 | HQ000658 | HQ001398 |
| 256 |  |  | HQ002288 | HQ000195 | HQ000659 | HQ001399 |
| 257 |  |  | HQ002289 | HQ000196 | HQ000660 | HQ001400 |
| 258 | FJ877458 | FJ878047 | HQ002290 | FJ877388 | FJ877244 | FJ877173 |
| 259 | FJ877459 | FJ878024 | HQ002291 | FJ877389 | FJ877245 | FJ877174 |
| 260 | FJ877460 | FJ878028 | HQ002292 | FJ877390 | FJ877246 | FJ877175 |
| 261 | HM355242 | HM353581 | HQ002293 | HM354301 | HQ000661 | HM354033 |
| 262 | HM355243 | HM353582 | HQ002294 | HM354302 | HQ000662 | HM354034 |
| 263 | FJ877461 | FJ878081 | HQ002295 | FJ877391 | FJ877247 | FJ877176 |
| 264 | FJ877462 | FJ878031 | HQ002296 |  | FJ877248 | FJ877177 |
| 265 | FJ877463 | FJ878034 | HQ002297 | FJ877392 | FJ877249 | FJ877178 |
| 267 | FJ877464 | FJ878063 | HQ002298 | FJ877393 | FJ877250 | FJ877179 |
| 268 | FJ877465 | FJ878038 | HQ002299 | FJ877394 | FJ877251 | FJ877180 |
| 269 | FJ877466 | FJ878035 | HQ002300 | FJ877395 | FJ877252 | FJ877181 |
| 270 | FJ877467 | FJ878070 | HQ002301 | FJ877396 | FJ877253 | FJ877182 |
| 271 | FJ877468 | FJ878030 | HQ002302 | FJ877397 | FJ877254 | FJ877183 |
| 272 | FJ877469 | FJ878069 | HQ002303 | FJ877398 | FJ877255 | FJ877184 |
| 273 | FJ877470 | FJ878026 | HQ002304 | FJ877399 | FJ877256 | FJ877185 |
| 274 | FJ877471 | FJ878043 | HQ002305 | FJ877400 | FJ877257 | FJ877186 |
| 275 | FJ877472 | FJ878027 | HQ002306 | FJ877401 | FJ877258 | FJ877187 |
| 276 | FJ877473 | FJ878079 | HQ002307 | FJ877402 | FJ877259 | FJ877188 |
| 277 | FJ877474 | FJ878052 | HQ002308 | FJ877403 | FJ877260 | FJ877189 |
| 278 | FJ877475 | FJ878046 |  | FJ877404 | FJ877261 | FJ877190 |
| 279 | FJ877476 | FJ878061 | HQ002309 | FJ877405 | FJ877262 | FJ877191 |
| 280 | FJ877477 | FJ878050 | HQ002310 | FJ877406 | FJ877263 | FJ877192 |
| 281 | FJ877478 | FJ878049 | HQ002311 | FJ877407 | FJ877264 | FJ877193 |
| 287 | FJ877484 | FJ878045 | HQ002312 | FJ877413 | FJ877270 | FJ877199 |
| 288 | FJ877485 | FJ878078 | HQ002313 | FJ877414 | FJ877271 | FJ877200 |
| 290 | FJ877487 | FJ878058 | HQ002314 | FJ877416 | FJ877273 | FJ877202 |
| 291 | FJ877488 | FJ878053 | HQ001725 | FJ877417 | FJ877274 | FJ877203 |
| 292 | FJ877489 | FJ878041 | HQ002315 | FJ877418 | FJ877275 | FJ877204 |
| 294 | FJ877491 | FJ878051 | HQ002316 | FJ877420 | FJ877277 | FJ877206 |
| 295 | FJ877492 | FJ878042 | HQ002317 | FJ877421 | FJ877278 | FJ877207 |
| 299 | FJ877496 | FJ878048 | HQ002318 | FJ877425 | FJ877282 | FJ877211 |
| 302 | FJ877499 | FJ878071 | HQ002319 | FJ877428 | FJ877285 | FJ877214 |
| 304 | FJ877501 | FJ878057 | HQ002320 | FJ877430 | FJ877287 | FJ877216 |
| 306 | FJ877503 | FJ878054 | HQ002321 | FJ877432 | FJ877289 | FJ877218 |
| 307 | FJ877504 | FJ878066 | HQ002322 | FJ877433 | FJ877290 | FJ877219 |
| 309 | FJ878506 |  | HQ002323 | FJ877435 | FJ877292 | FJ877221 |
| 310 | FJ877507 |  | HQ002324 | FJ877436 | FJ877293 | FJ877222 |
| 311 | FJ876508 |  | HQ001727 | FJ877437 | FJ877294 | FJ877223 |
| 312 | FJ875509 |  | HQ002324 | FJ877438 | FJ877295 | FJ877224 |
| 313 | FJ874510 |  | HQ002325 | FJ877439 | FJ877296 | FJ877225 |
| 314 | FJ873511 |  | HQ001728 | FJ877440 | FJ877297 | FJ877226 |
| 315 | FJ872512 |  | HQ001729 | FJ877441 | FJ877298 | FJ877227 |
| 316 | FJ871513 |  | HQ002326 | FJ877442 | FJ877299 | FJ877228 |
| 396 | HM355244 | HM353583 | HQ001730 | HM354303 | HQ000663 | HM354035 |
| 397 | HM355245 | HM353584 | HQ001731 | HM354304 | HQ000664 | HM354036 |
| 398 | HM355246 | HM353585 | HQ001732 | HM354305 | HQ000665 | HM354037 |
| 399 | HM355247 | HM353586 | HQ001733 | HM354306 | HQ000666 | HM354038 |
| 400 | HM355248 | HM353587 | HQ001734 | HM354307 | HQ000667 | HM354039 |
| 401 | HM355249 | HM353588 | HQ001735 | HM354308 | HQ000668 | HM354040 |
| 402 | HM355250 | HM353589 | HQ001736 | HM354309 | HQ000669 | HM354041 |
| 403 | HM355251 | HM353590 | HQ001737 | HM354310 | HQ000670 | HM354042 |
| 404 | HM355252 | HM353591 | HQ001738 | HM354311 | HQ000671 | HM354043 |
| 405 | HM355253 | HM353592 | HQ002327 | HM354312 | HQ000672 | HM354044 |
| 406 | HM355254 | HM353593 | HQ001739 | HM354313 | HQ000673 | HM354045 |
| 407 | HM355255 | HM353594 | HQ001740 | HM354314 | HQ000674 | HM354046 |
| 408 | HM355256 | HM353595 | HQ001741 | HM354315 | HQ000675 | HM354047 |
| 409 | HM355257 | HM353596 | HQ002328 | HM354316 | HQ000676 | HM354048 |
| 410 | HM355258 | HM353597 | HQ001742 | HM354317 | HQ000677 | HM354049 |
| 411 | HM355259 | HM353598 | HQ001743 | HM354318 | HQ000678 | HM354050 |
| 412 | HM355260 | HM353599 | HQ001744 | HM354319 | HQ000679 | HM354051 |
| 413 | HM355261 | HM353600 | HQ001745 | HM354320 | HQ000680 | HM354052 |
| 414 | HM355262 | HM353601 | HQ001746 | HM354321 | HQ000681 | HM354053 |
| 415 | HM355263 | HM353602 | HQ002329 | HM354322 | HQ000682 | HM354054 |
| 416 | HM355264 | HM353603 | HQ001747 | HM354323 | HQ000683 | HM354055 |
| 417 | HM355265 | HM353604 | HQ002330 | HM354324 | HQ000684 | HM354056 |
| 418 | HM355266 | HM353605 | HQ001748 | HM354325 | HQ000685 | HM354057 |
| 419 | HM355267 | HM353606 | HQ001749 | HM354326 | HQ000686 | HM354058 |
| 420 | HM355268 | HM353607 | HQ001750 | HM354327 | HQ000687 | HM354059 |
| 421 | HM355269 | HM353608 | HQ001751 | HM354328 | HQ000688 | HM354060 |
| 422 | HM355270 | HM353609 | HQ001752 | HM354329 | HQ000689 | HM354061 |
| 423 | HM355271 | HM353610 | HQ001753 | HM354330 | HQ000690 | HM354062 |
| 424 | HM355272 | HM353611 | HQ002331 | HM354331 | HQ000691 | HM354063 |
| 425 | HM355273 | HM353612 | HQ001754 | HM354332 | HQ000692 | HM354064 |
| 426 | HM355274 | HM353613 | HQ001755 | HM354333 | HQ000693 | HM354065 |
| 427 | HM355275 | HM353614 | HQ002332 | HM354334 | HQ000694 | HM354066 |
| 428 | HM355276 | HM353615 | HQ001756 | HM354335 | HQ000695 | HM354067 |
| 429 | HM355277 | HM353616 | HQ001757 | HM354336 | HQ000696 | HM354068 |
| 430 | HM355278 | HM353617 | HQ001758 | HM354337 | HQ000697 | HM354069 |
| 431 | HM355279 | HM353618 | HQ001759 | HM354338 | HQ000698 | HM354070 |
| 432 | HM355280 | HM353619 | HQ001760 | HM354339 | HQ000699 | HM354071 |
| 433 | HM355281 | HM353620 | HQ001761 | HM354340 | HQ000700 | HM354072 |
| 434 | HM355282 | HM353621 | HQ001762 | HM354341 | HQ000701 | HM354073 |
| 435 | HM355283 | HM353622 | HQ001763 | HM354342 | HQ000702 | HM354074 |
| 436 | HM355284 | HM353623 | HQ001764 | HM354343 | HQ000703 | HM354075 |
| 437 | HM355285 | HM353624 | HQ001765 | HM354344 | HQ000704 | HM354076 |
| 438 | HM355286 | HM353625 | HQ001766 | HM354345 | HQ000705 | HM354077 |
| 439 | HM355287 | HM353626 | HQ001767 | HM354346 | HQ000706 | HM354078 |
| 440 | HM355288 | HM353627 | HQ002333 | HM354347 | HQ000707 | HM354079 |
| 441 | HM355289 | HM353628 | HQ001768 | HM354348 | HQ000708 | HM354080 |
| 442 | HM355290 | HM353629 | HQ001769 | HM354349 | HQ000709 | HM354081 |
| 443 | HM355291 | HM353630 | HQ001770 | HM354350 | HQ000710 | HM354082 |
| 444 | HM355292 | HM353631 | HQ001771 | HM354351 | HQ000711 | HM354083 |
| 445 | HM355293 | HM353632 | HQ001772 | HM354352 | HQ000712 | HM354084 |
| 446 | HM355294 | HM353633 | HQ001773 | HM354353 | HQ000713 | HM354085 |
| 447 | HM355295 | HM353634 | HQ001774 | HM354354 | HQ000714 | HM354086 |
| 448 | HM355296 | HM353635 | HQ001775 | HM354355 | HQ000715 | HM354087 |
| 449 | HM355297 | HM353636 | HQ001776 | HM354356 | HQ000716 | HM354088 |
| 450 | HM355298 | HM353637 | HQ001777 | HM354357 | HQ000717 | HM354089 |
| 451 | HM355299 | HM353638 | HQ001778 | HM354358 | HQ000718 | HM354090 |
| 452 | HM355300 | HM353639 | HQ001779 | HM354359 | HQ000719 | HM354091 |
| 453 | HM355301 | HM353640 | HQ001780 | HM354360 | HQ000720 | HM354092 |
| 454 | HM355302 | HM353641 | HQ001781 | HM354361 | HQ000721 | HM354093 |
| 455 | HM355303 | HM353642 | HQ001782 | HM354362 | HQ000722 | HM354094 |
| 456 | HM355304 | HM353643 | HQ001783 | HM354363 | HQ000723 | HM354095 |
| 457 | HM355305 | HM353644 | HQ002334 | HM354364 | HQ000724 | HM354096 |
| 458 | HM355306 | HM353645 | HQ001784 | HM354365 | HQ000725 | HM354097 |
| 459 | HM355307 | HM353646 | HQ002335 | HM354366 | HQ000726 | HM354098 |
| 460 | HM355308 | HM353647 | HQ001785 | HM354367 | HQ000727 | HM354099 |
| 461 | HM355309 | HM353648 | HQ001786 | HM354368 | HQ000728 | HM354100 |
| 462 | HM355310 | HM353649 | HQ001787 | HM354369 | HQ000729 | HM354101 |
| 463 | HM355311 | HM353650 | HQ001788 | HM354370 | HQ000730 | HM354102 |
| 464 | HM355312 | HM353651 | HQ001789 | HM354371 | HQ000731 | HM354103 |
| 465 | HM355313 | HM353652 | HQ001790 | HM354372 | HQ000732 | HM354104 |
| 466 | HM355314 | HM353653 | HQ001791 | HM354373 | HQ000733 | HM354105 |
| 467 | HM355315 | HM353654 | HQ001792 | HM354374 | HQ000734 | HM354106 |
| 468 | HM355316 | HM353655 | HQ002336 | HM354375 | HQ000735 | HM354107 |
| 469 | HM355317 | HM353656 | HQ001793 | HM354376 | HQ000736 | HM354108 |
| 470 | HM355318 | HM353657 | HQ002337 | HM354377 | HQ000737 | HM354109 |
| 471 | HM355319 | HM353658 | HQ002338 | HM354378 | HQ000738 | HM354110 |
| 472 | HM355320 | HM353659 | HQ001794 | HM354379 | HQ000739 | HM354111 |
| 474 | HM355321 | HM353660 | HQ001795 | HM354380 | HQ000740 | HM354112 |
| 475 | HM355322 | HM353661 | HQ001796 | HM354381 | HQ000741 | HM354113 |
| 640 | HM355323 | HM353662 | HQ002339 | HM354382 | HQ000742 | HM354114 |
| 642 | HM355324 | HM353663 | HQ002340 | HM354383 | HQ000743 | HM354115 |
| 643 | HM355325 | HM353664 | HQ002341 | HM354384 | HQ000744 | HM354116 |
| 646 | HM355326 | HM353665 | HQ002342 | HM354385 | HQ000745 | HM354117 |
| 648 | HM355327 | HM353666 | HQ001797 | HM354386 | HQ000746 | HM354118 |
| 650 | HM355328 | HM353667 | HQ001798 | HM354387 | HQ000747 | HM354119 |
| 651 | HM355329 | HM353668 | HQ002343 | HM354388 | HQ000748 | HM354120 |
| 652 | HM355330 | HM353669 | HQ001799 | HM354389 | HQ000749 | HM354121 |
| 655 | HM355331 | HM353670 | HQ002344 | HM354390 | HQ000750 | HM354122 |
| 656 | HM355332 | HM353671 | HQ002345 | HM354391 | HQ000751 | HM354123 |
| 657 | HM355333 | HM353672 | HQ002346 | HM354392 | HQ000752 | HM354124 |
| 658 | HM355334 | HM353673 | HQ002347 | HM354393 | HQ000753 | HM354125 |
| 659 | HM355335 | HM353674 | HQ002348 | HM354394 | HQ000754 | HM354126 |
| 660 | HM355336 | HM353675 | HQ002349 | HM354395 | HQ000755 | HM354127 |
| 661 | HM355337 | HM353676 | HQ002350 | HM354396 | HQ000756 | HM354128 |
| 662 | HM355338 | HM353677 | HQ001800 | HM354397 | HQ000757 | HM354129 |
| 663 | HM355339 | HM353678 | HQ002351 | HM354398 | HQ000758 | HM354130 |
| 664 | HM355340 | HM353679 | HQ002352 | HM354399 | HQ000759 | HM354131 |
| 666 | HM355341 | HM353680 | HQ002353 | HM354400 | HQ000760 | HM354132 |
| 667 | HM355342 | HM353681 | HQ002354 | HM354401 | HQ000761 | HM354133 |
| 670 | HM355343 | HM353682 | HQ001801 | HM354402 | HQ000762 | HM354134 |
| 671 | HM355344 | HM353683 | HQ001802 | HM354403 | HQ000763 | HM354135 |
| 672 | HM355345 | HM353684 | HQ002355 | HM354404 | HQ000764 | HM354136 |
| 673 | HM355346 | HM353685 | HQ002356 | HM354405 | HQ000765 | HM354137 |
| 674 | HM355347 | HM353686 | HQ002357 | HM354406 | HQ000766 | HM354138 |
| 675 | HM355348 | HM353687 | HQ001803 | HM354407 | HQ000767 | HM354139 |
| 676 | HM355349 | HM353688 | HQ002358 | HM354408 | HQ000768 | HM354140 |
| 677 | HM355350 | HM353689 | HQ002359 | HM354409 | HQ000769 | HM354141 |
| 678 | HM355351 | HM353690 | HQ002360 | HM354410 | HQ000770 | HM354142 |
| 679 | HM355352 | HM353691 | HQ002361 | HM354411 | HQ000771 | HM354143 |
| 680 | HM355353 | HM353692 | HQ002362 | HM354412 | HQ000772 | HM354144 |
| 682 | HM355354 | HM353693 | HQ001804 | HM354413 | HQ000773 | HM354145 |
| 683 | HM355355 | HM353694 | HQ002363 | HM354414 | HQ000774 | HM354146 |
| 684 | HM355356 | HM353695 | HQ001805 | HM354415 | HQ000775 | HM354147 |
| 685 | HM355357 | HM353696 | HQ001806 | HM354416 | HQ000776 | HM354148 |
| 686 | HM355358 | HM353697 | HQ002364 | HM354417 | HQ000777 | HM354149 |
| 688 | HM355359 | HM353698 | HQ001807 | HM354418 | HQ000778 | HM354150 |
| 695 | HM355360 | HM353699 | HQ01808 | HM354419 | HQ000779 | HM354151 |
| 696 | HM355361 | HM353700 | HQ002365 | HM354420 | HQ000780 | HM354152 |
| 697 | HM355362 | HM353701 | HQ002366 | HM354421 | HQ000781 | HM354153 |
| 698 | HM355363 | HM353702 | HQ002367 | HM354422 | HQ000782 | HM354154 |
| 701 | HM355364 | HM353703 | HQ002368 | HM354423 | HQ000783 | HM354155 |
| 702 | HM355365 | HM353704 | HQ001809 | HM354424 | HQ000784 | HM354156 |
| 703 | HM355366 | HM353705 | HQ002369 | HM354425 | HQ000785 | HM354157 |
| 704 | HM355367 | HM353706 | HQ001810 | HM354426 | HQ000786 | HM354158 |
| 708 | HM355368 | HM353707 | HQ001811 | HM354427 | HQ000787 | HM354159 |
| 709 | HM355369 | HM353708 | HQ001812 | HM354428 | HQ000788 | HM354160 |
| 711 | HM355370 | HM353709 | HQ002370 | HM354429 | HQ000789 | HM354161 |
| 712 | HM355371 | HM353710 | HQ001813 | HM354430 | HQ000790 | HM354162 |
| 719 | HM355372 | HM353711 | HQ002371 | HM354431 | HQ000791 | HM354163 |
| 899 | HQ002467 | HQ002688 | HQ002372 | HQ000197 | HQ000792 | HQ001401 |
| 1179 | HQ002468 | HQ002672 | HQ002247 | FJ877372 | FJ877230 | FJ877157 |
| 1227 | HQ002471 |  | HQ002248 | HQ000177 | HQ000586 | HQ001381 |
| 1027 | HM355105 | HM353444 | HQ001634 | HM354164 | HQ000506 | HM353896 |
| 1028 | HM355106 | HM353445 | HQ001635 | HM354165 | HQ000507 | HM353897 |
| 1029 | HM355107 | HM353446 | HQ002216 | HM354166 | HQ000508 | HM353898 |
| 1030 | HM355108 | HM353447 | HQ001636 | HM354167 | HQ000509 | HM353899 |
| 1031 | HM355109 | HM353448 | HQ002217 | HM354168 | HQ000510 | HM353900 |
| 1032 | HM355110 | HM353449 | HQ001637 | HM354169 | HQ000511 | HM353901 |
| 1033 | HM355111 | HM353450 | HQ002218 | HM354170 | HQ000512 | HM353902 |
| 1034 | HM355112 | HM353451 | HQ002219 | HM354171 | HQ000513 | HM353903 |
| 1035 | HM355113 | HM353452 | HQ001638 | HM354172 | HQ000514 | HM353904 |
| 1036 | HM355114 | HM353453 | HQ002220 | HM354173 | HQ000515 | HM353905 |
| 1037 | HM355115 | HM353454 | HQ001639 | HM354174 | HQ000516 | HM353906 |
| 1038 | HM355116 | HM353455 | HQ002221 | HM354175 | HQ000517 | HM353907 |
| 1039 | HM355117 | HM353456 | HQ002222 | HM354176 | HQ000518 | HM353908 |
| 1040 | HM355118 | HM353457 | HQ002223 | HM354177 | HQ000519 | HM353909 |
| 1041 | HM355119 | HM353458 | HQ001640 | HM354178 | HQ000520 | HM353910 |
| 1042 | HM355120 | HM353459 | HQ002224 | HM354179 | HQ000521 | HM353911 |
| 1043 | HM355121 | HM353460 | HQ001641 | HM354180 | HQ000522 | HM353912 |
| 1044 | HM355122 | HM353461 | HQ001642 | HM354181 | HQ000523 | HM353913 |
| 1045 | HM355123 | HM353462 | HQ001643 | HM354182 | HQ000524 | HM353914 |
| 1046 | HM355124 | HM353463 | HQ002225 | HM354183 | HQ000525 | HM353915 |
| 1047 | HM355125 | HM353464 | HQ002226 | HM354184 | HQ000526 | HM353916 |
| 1048 | HM355126 | HM353465 | HQ001644 | HM354185 | HQ000527 | HM353917 |
| 1049 | HM355127 | HM353466 | HQ001645 | HM354186 | HQ000528 | HM353918 |
| 1050 | HM355128 | HM353467 | HQ001646 | HM354187 | HQ000529 | HM353919 |
| 1051 | HM355129 | HM353468 | HQ002227 | HM354188 | HQ000530 | HM353920 |
| 1052 | HM355130 | HM353469 | HQ002228 | HM354189 | HQ000531 | HM353921 |
| 1053 | HM355131 | HM353470 | HQ002229 | HM354190 | HQ000532 | HM353922 |
| 1054 | HM355132 | HM353471 | HQ002230 | HM354191 | HQ000533 | HM353923 |
| 1055 | HM355133 | HM353472 | HQ001647 | HM354192 | HQ000534 | HM353924 |
| 1056 | HM355134 | HM353473 | HQ001648 | HM354193 | HQ000535 | HM353925 |
| 1057 | HM355135 | HM353474 | HQ002231 | HM354194 | HQ000536 | HM353926 |
| 1058 | HM355136 | HM353475 | HQ002232 | HM354195 | HQ000537 | HM353927 |
| 1059 | HM355137 | HM353476 | HQ001649 | HM354196 | HQ000538 | HM353928 |
| 1060 | HM355138 | HM353477 | HQ001650 | HM354197 | HQ000539 | HM353929 |
| 1061 | HM355139 | HM353478 | HQ001651 | HM354198 | HQ000540 | HM353930 |
| 1062 | HM355140 | HM353479 | HQ001652 | HM354199 | HQ000541 | HM353931 |
| 1063 | HM355141 | HM353480 | HQ001653 | HM354200 | HQ000542 | HM353932 |
| 1064 | HM355142 | HM353481 | HQ001654 | HM354201 | HQ000543 | HM353933 |
| 1065 | HM355143 | HM353482 | HQ002233 | HM354202 | HQ000544 | HM353934 |
| 1066 | HM355144 | HM353483 | HQ001655 | HM354203 | HQ000545 | HM353935 |
| 1067 | HM355145 | HM353484 | HQ001656 | HM354204 | HQ000546 | HM353936 |
| 1068 | HM355146 | HM353485 | HQ001657 | HM354205 | HQ000547 | HM353937 |
| 1069 | HM355147 | HM353486 | HQ002234 | HM354206 | HQ000548 | HM353938 |
| 1070 | HM355148 | HM353487 | HQ001658 | HM354207 | HQ000549 | HM353939 |
| 1071 | HM355149 | HM353488 | HQ001659 | HM354208 | HQ000550 | HM353940 |
| 1072 | HM355150 | HM353489 | HQ002235 | HM354209 | HQ000551 | HM353941 |
| 1073 | HM355151 | HM353490 | HQ002236 | HM354210 | HQ000552 | HM353942 |
| 1074 | HM355152 | HM353491 | HQ002237 | HM354211 | HQ000553 | HM353943 |
| 1075 | HM355153 | HM353492 | HQ001660 | HM354212 | HQ000554 | HM353944 |
| 1076 | HM355154 | HM353493 | HQ001661 | HM354213 | HQ000555 | HM353945 |
| 1077 | HM355155 | HM353494 | HQ001662 | HM354214 | HQ000556 | HM353946 |
| 1078 | HM355156 | HM353495 | HQ001663 | HM354215 | HQ000557 | HM353947 |
| 1079 | HM355157 | HM353496 | HQ002238 | HM354216 | HQ000558 | HM353948 |
| 1080 | HM355158 | HM353497 | HQ001664 | HM354217 | HQ000559 | HM353949 |
| 1081 | HM355159 | HM353498 | HQ001665 | HM354218 | HQ000560 | HM353950 |
| 1082 | HM355160 | HM353499 | HQ001666 | HM354219 | HQ000561 | HM353951 |
| 1083 | HM355161 | HM353500 | HQ002239 | HM354220 | HQ000562 | HM353952 |
| 1084 | HM355162 | HM353501 | HQ001667 | HM354221 | HQ000563 | HM353953 |
| 1085 | HM355163 | HM353502 | HQ001668 | HM354222 | HQ000564 | HM353954 |
| 1086 | HM355164 | HM353503 | HQ001669 | HM354223 | HQ000565 | HM353955 |
| 1087 | HM355165 | HM353504 | HQ001670 | HM354224 | HQ000566 | HM353956 |
| 1088 | HM355166 | HM353505 | HQ001671 | HM354225 | HQ000567 | HM353957 |
| 1089 | HM355167 | HM353506 | HQ002240 | HM354226 | HQ000568 | HM353958 |
| 1090 | HM355168 | HM353507 | HQ002241 | HM354227 | HQ000569 | HM353959 |
| 1091 | HM355169 | HM353508 | HQ001672 | HM354228 | HQ000570 | HM353960 |
| 1092 | HM355170 | HM353509 | HQ001673 | HM354229 | HQ000571 | HM353961 |
| 1093 | HM355171 | HM353510 | HQ001674 | HM354230 | HQ000572 | HM353962 |
| 1094 | HM355172 | HM353511 | HQ001675 | HM354231 | HQ000573 | HM353963 |
| 1095 | HM355173 | HM353512 | HQ002242 | HM354232 | HQ000574 | HM353964 |
| 1096 | HM355174 | HM353513 | HQ001676 | HM354233 | HQ000575 | HM353965 |
| 1097 | HM355175 | HM353514 | HQ002243 | HM354234 | HQ000576 | HM353966 |
| 1098 | HM355176 | HM353515 | HQ001677 | HM354235 | HQ000577 | HM353967 |
| 1099 | HM355177 | HM353516 | HQ002244 | HM354236 | HQ000578 | HM353968 |
| 1100 | HM355178 | HM353517 | HQ002245 | HM354237 | HQ000579 | HM353969 |
| 1101 | HM355179 | HM353518 | HQ001678 | HM354238 | HQ000580 | HM353970 |
| 1102 | HM355180 | HM353519 | HQ002246 | HM354239 | HQ000581 | HM353971 |
| 1103 | HM355181 | HM353520 | HQ001679 | HM354240 | HQ000582 | HM353972 |
| 1104 | HM355182 | HM353521 | HQ001680 | HM354241 | HQ000583 | HM353973 |
| 1105 | HM355183 | HM353522 | HQ001681 | HM354242 | HQ000584 | HM353974 |
| 1106 | HM355184 | HM353523 | HQ001682 | HM354243 | HQ000585 | HM353975 |
| 1229 | HM355185 | HM353524 | HQ002249 | HM354244 | HQ000587 | HM353976 |
| 1230 | HM355186 | HM353525 | HQ002250 | HM354245 | HQ000588 | HM353977 |
| 1233 | HM355187 | HM353526 | HQ001683 | HM354246 | HQ000589 | HM353978 |
| 1237 | HM355188 | HM353527 | HQ002251 | HM354247 | HQ000590 | HM353979 |
| 1239 | HM355189 | HM353528 | HQ001684 | HM354248 | HQ000591 | HM353980 |
| 1241 | HM355190 | HM353529 | HQ001685 | HM354249 | HQ000592 | HM353981 |
| 1245 | HM355191 | HM353530 | HQ002252 | HM354250 | HQ000593 | HM353982 |
| 1249 | HM355192 | HM353531 | HQ002253 | HM354251 | HQ000594 | HM353983 |
| 1250 | HM355193 | HM353532 | HQ001686 | HM354252 | HQ000595 | HM353984 |
| 1251 | HM355194 | HM353533 | HQ001687 | HM354253 | HQ000596 | HM353985 |
| 1252 | HM355195 | HM353534 | HQ001688 | HM354254 | HQ000597 | HM353986 |
| 1253 | HM355196 | HM353535 | HQ001689 | HM354255 | HQ000598 | HM353987 |
| 1254 | HM355197 | HM353536 | HQ002254 | HM354256 | HQ000599 | HM353988 |
| 1255 | HM355198 | HM353537 | HQ001690 | HM354257 | HQ000600 | HM353989 |
| 1257 | HM355199 | HM353538 | HQ001691 | HM354258 | HQ000601 | HM353990 |
| 1258 | HM355200 | HM353539 | HQ001692 | HM354259 | HQ000602 | HM353991 |
| 1260 | HM355201 | HM353540 | HQ002255 | HM354260 | HQ000603 | HM353992 |
| 1262 | HM355202 | HM353541 | HQ001693 | HM354261 | HQ000604 | HM353993 |
| 1264 | HM355203 | HM353542 | HQ002256 | HM354262 | HQ000605 | HM353994 |
| 1265 | HM355204 | HM353543 | HQ001694 | HM354263 | HQ000606 | HM353995 |
| 1266 | HM355205 | HM353544 | HQ001695 | HM354264 | HQ000607 | HM353996 |
| 1268 | HM355206 | HM353545 | HQ001696 | HM354265 | HQ000608 | HM353997 |
| 1269 | HM355207 | HM353546 | HQ001697 | HM354266 | HQ000609 | HM353998 |
| 1270 | HM355208 | HM353547 | HQ002257 | HM354267 | HQ000610 | HM353999 |
| 1271 | HM355209 | HM353548 | HQ001698 | HM354268 | HQ000611 | HM354000 |
| 1272 | HM355210 | HM353549 | HQ001699 | HM354269 | HQ000612 | HM354001 |
| 1274 | HM355211 | HM353550 | HQ002258 | HM354270 | HQ000613 | HM354002 |
| 1275 | HM355212 | HM353551 | HQ001700 | HM354271 | HQ000614 | HM354003 |
| 1276 | HM355213 | HM353552 | HQ001701 | HM354272 | HQ000615 | HM354004 |
| 1277 | HM355214 | HM353553 | HQ001702 | HM354273 | HQ000616 | HM354005 |
| 1279 | HM355215 | HM353554 | HQ002259 | HM354274 | HQ000617 | HM354006 |
| 1280 | HM355216 | HM353555 | HQ001703 | HM354275 | HQ000618 | HM354007 |
| 1281 | HM355217 | HM353556 | HQ001704 | HM354276 | HQ000619 | HM354008 |
| 1282 | HM355218 | HM353557 | HQ001705 | HM354277 | HQ000620 | HM354009 |
| 1290 | HM355219 | HM353558 | HQ001706 | HM354278 | HQ000621 | HM354010 |
| 1291 | HM355220 | HM353559 | HQ001707 | HM354279 | HQ000622 | HM354011 |
| 1293 | HM355221 | HM353560 | HQ002260 | HM354280 | HQ000623 | HM354012 |
| 1295 | HM355222 | HM353561 | HQ002261 | HM354281 | HQ000624 | HM354013 |
| 1296 | HM355223 | HM353562 | HQ001708 | HM354282 | HQ000625 | HM354014 |
| 1297 | HM355224 | HM353563 | HQ001709 | HM354283 | HQ000626 | HM354015 |
| 1303 | HM355225 | HM353564 | HQ001710 | HM354284 | HQ000627 | HM354016 |
| 1304 | HM355226 | HM353565 | HQ001711 | HM354285 | HQ000628 | HM354017 |
| 1305 | HM355227 | HM353566 | HQ002262 | HM354286 | HQ000629 | HM354018 |
| 1306 | HM355228 | HM353567 | HQ001712 | HM354287 | HQ000630 | HM354019 |
| 1307 | HM355229 | HM353568 | HQ001713 | HM354288 | HQ000631 | HM354020 |

IC numbers for U.S.A. strains (203-316; 899; 1179; 1227)

IC numbers for Argentina strains (396-475)

IC numbers for Australia strains (640-719)

IC numbers for Benin strains (1027-1106)

IC numbers for India strains (1229-1307)
