# Supplementary material for: Global population structure and adaptive evolution of aflatoxin‐producing fungi
Source: Ecol Evol. 2017 Sep 30;7(21):9179–91. doi: 10.1002/ece3.3464 (PMC5677503; doi:10.1002/ece3.3464)
Supplement: Supplementary file 15 [file ECE3-7-9179-s015.docx]

Table S3. GenBank accession numbers for *A. flavus* S-strain sequences used in this study

| **IC Strain** | ***W/X*** | ***M/N*** | ***MAT*** | ***amdS*** | ***mfs*** | ***trpC*** |
| --- | --- | --- | --- | --- | --- | --- |
| 1112^a^ | HM745674 | HM745864 | HQ001882 | HM745788 | HQ000417 | HM745598 |
| 1113 ^a^ | HM745675 | HM745865 | HQ001883 | HM745789 | HQ000418 | HM745599 |
| 1117 ^a^ | HQ002415 | HQ002812 | HQ002055 | HQ000326 | HQ000419 | HQ001186 |
| 1118 ^a^ | HQ002377 | HQ002816 | HQ001884 | HQ000327 | HQ000420 | HQ001187 |
| 1119 ^a^ | HQ002374 | HQ002839 | HQ001885 | HQ000328 | HQ000421 | HQ001188 |
| 1120 ^a^ | HM745676 | HM745866 | HQ001886 | HM745790 | HQ000422 | HM745600 |
| 1121 ^a^ | HQ002376 | HQ002808 | HQ001887 | HQ000329 | HQ000423 | HQ001189 |
| 1133 ^a^ | HQ002378 | HQ002805 | HQ001888 | HQ000330 | HQ000424 | HQ001190 |
| 1134 ^a^ | HQ002373 | HQ002804 | HQ002056 | HQ000331 | HQ000425 | HQ001191 |
| 1135 ^a^ | HQ002375 | HQ002813 | HQ001889 | HQ000332 | HQ000426 | HQ001192 |
| 1140 ^a^ | HM745677 | HM745867 | HQ002057 | HM745791 | HQ000427 | HM745601 |
| 1141 ^a^ | HQ002379 | HQ002840 | HQ001890 | HQ000333 | HQ000428 | HQ001193 |
| 1142 ^a^ | HM745678 | HM745868 | HQ002058 | HM745792 | HQ000429 | HM745602 |
| 1144 ^a^ | HQ002380 | HQ002841 | HQ002059 | HQ000334 | HQ000430 | HQ001194 |
| 1145 ^a^ | HQ002408 | HQ002806 | HQ002060 | HQ000335 | HQ000431 | HQ001195 |
| 1146 ^a^ | HQ002384 | HQ002801 | HQ002061 | HQ000336 | HQ000432 | HQ001196 |
| 1147 ^a^ | HQ002410 | HQ002807 | HQ002062 | HQ000337 | HQ000433 | HQ001197 |
| 1148 ^a^ | HQ002399 | HQ002815 | HQ002063 | HQ000338 | HQ000434 | HQ001198 |
| 1149 ^a^ | HQ002388 | HQ002814 | HQ002064 | HQ000339 | HQ000435 | HQ001199 |
| 1150 ^a^ | HM745679 | HM745869 | HQ002065 | HM745793 | HQ000436 | HM745603 |
| 1151 ^a^ | HQ002423 | HQ002800 | HQ002066 | HQ000340 | HQ000437 | HQ001200 |
| 1152 ^b^ | HM745680 | HM745870 | HQ002067 | HM745794 | HQ000438 | HM745604 |
| 1153 ^b^ | HM745681 | HM745871 | HQ001891 | HM745795 | HQ000439 | HM745605 |
| 1154 ^b^ | HM745682 | HM745872 | HQ002068 | HM745796 | HQ000440 | HM745606 |
| 1155 ^b^ | HM745683 | HM745873 | HQ002069 | HM745797 | HQ000441 | HM745607 |
| 1156 ^b^ | HQ002381 | HQ002802 | HQ002070 | HQ000341 | HQ000442 | HQ001201 |
| 1157 ^b^ | HM745684 | HM745874 | HQ002071 | HM745798 | HQ000443 | HM745608 |
| 1160 ^b^ | HM745685 | HM745875 | HQ001892 | HM745799 | HQ000444 | HM745609 |
| 1161 ^b^ | HM745686 | HM745876 | HQ001893 | HM745800 | HQ000445 | HM745610 |
| 1162 ^b^ | HM745687 | HM745877 | HQ001894 | HM745801 | HQ000446 | HM745611 |
| 1163 ^b^ | HM745688 | HM745878 | HQ002072 | HM745802 | HQ000447 | HM745612 |
| 1164 ^b^ | HM745689 | HM745879 | HQ001895 | HM745803 | HQ000448 | HM745613 |
| 1165 ^b^ | HM745690 | HM745880 | HQ001896 | HM745804 | HQ000449 | HM745614 |
| 1167 ^b^ | HQ002406 | HQ002803 | HQ001897 | HQ000342 | HQ000450 | HQ001202 |
| 1168 ^b^ | HM745691 | HM745881 | HQ001898 | HM745805 | HQ000451 | HM745615 |
| 1169 ^b^ | HM745692 | HM745882 | HQ001899 | HM745806 | HQ000452 | HM745616 |
| 1171 ^b^ | HM745693 | HM745883 | HQ001900 | HM745807 | HQ000453 | HM745617 |
| 1174 ^b^ | HM745694 | HM745884 | HQ001901 | HM745808 | HQ000454 | HM745618 |
| 1175 ^b^ | HM745695 | HM745885 | HQ001902 | HM745809 | HQ000455 | HM745619 |
| 1176 ^b^ | HM745696 | HM745886 | HQ001903 | HM745810 | HQ000456 | HM745620 |
| 1177 ^b^ | HM745697 | HM745887 | HQ002073 | HM745811 | HQ000457 | HM745621 |
| 1178 ^b^ | HM745698 | HM745888 | HQ001904 | HM745812 | HQ000458 | HM745622 |
| 1228 ^b^ | HQ002405 |  | HQ001905 | HQ000343 | HQ000459 | HQ001203 |
| 476 ^b^ | HM745699 | HM745889 | HQ002074 | HM745813 | HQ000460 | HM745623 |
| 477 ^a^ | HM745700 | HM745890 | HQ001906 | HM745814 | HQ000461 | HM745624 |
| 478 ^a^ | HM745701 | HM745891 | HQ001907 | HM745815 | HQ000462 | HM745625 |
| 479 ^b^ | HM745702 | HM745892 | HQ002075 | HM745816 | HQ000463 | HM745626 |
| 720 ^a^ | HM745703 | HM745893 | HQ001908 | HM745817 | HQ000464 | HM745627 |
| 723 ^b^ | HM745704 | HM745894 | HQ002076 | HM745818 | HQ000465 | HM745628 |
| 725 ^b^ | HQ002383 | HQ002849 | HQ002077 | HQ000344 | HQ000466 | HQ001204 |
| 727 ^b^ | HQ002422 | HQ002847 | HQ002078 | HQ000345 | HQ000467 | HQ001205 |
| 728 ^b^ | HQ002409 | HQ002831 | HQ002079 | HQ000346 | HQ000468 | HQ001206 |
| 729 ^b^ | HQ002387 | HQ002845 | HQ002080 | HQ000347 | HQ000469 | HQ001207 |
| 731 ^a^ | HQ002385 | HQ002827 | HQ001909 | HQ000348 | HQ000470 | HQ001208 |
| 732 ^b^ | HQ002395 | HQ002846 | HQ001910 | HQ000349 | HQ000471 | HQ001209 |
| 733 ^a^ | HQ002417 | HQ002826 | HQ001911 | HQ000350 | HQ000472 | HQ001210 |
| 735 ^a^ | HQ002411 | HQ002836 | HQ001912 | HQ000351 | HQ000473 | HQ001211 |
| 736 ^b^ | HQ002414 | HQ002848 | HQ002081 | HQ000352 | HQ000474 | HQ001212 |
| 737 ^b^ | HQ002420 | HQ002835 | HQ001914 | HQ000353 | HQ000475 | HQ001213 |
| 741 ^b^ | HQ002413 | HQ002844 | HQ002083 | HQ000354 | HQ000476 | HQ001214 |
| 742 ^a^ | HQ002393 | HQ002825 | HQ001913 | HQ000355 | HQ000477 | HQ001215 |
| 743 ^b^ | HQ002421 | HQ002809 | HQ002084 | HQ000356 | HQ000478 | HQ001216 |
| 744 ^a^ | HM745705 | HM745895 | HQ001914 | HM745819 | HQ000479 | HM745629 |
| 748 ^b^ | HQ002403 | HQ002843 | HQ002085 | HQ000357 | HQ000480 | HQ001217 |
| 749 ^b^ | HQ002416 | HQ002842 | HQ002086 | HQ000358 | HQ000481 | HQ001218 |
| 751 ^b^ | HQ002401 | HQ002838 | HQ002087 | HQ000359 | HQ000482 | HQ001219 |
| 753 ^b^ | HQ002418 | HQ002811 | HQ002088 | HQ000360 | HQ000483 | HQ001220 |
| 755 ^b^ | HM745706 | HM745896 | HQ002089 | HM745820 | HQ000484 | HM745630 |
| 758 ^b^ | HQ002389 | HQ002810 | HQ002090 | HQ000361 | HQ000485 | HQ001221 |
| 760 ^b^ | HM745707 | HM745897 | HQ002091 | HM745821 | HQ000486 | HM745631 |
| 762 ^b^ | HQ002400 | HQ002829 | HQ002092 | HQ000362 | HQ000487 | HQ001222 |
| 768 ^b^ | HM745708 | HM745898 | HQ002093 | HM745822 | HQ000488 | HM745632 |
| 770 ^b^ | HQ002390 | HQ002833 | HQ002094 | HQ000363 | HQ000489 | HQ001223 |
| 777 ^b^ | HQ002402 | HQ002830 | HQ002095 | HQ000364 | HQ000490 | HQ001224 |
| 778 ^a^ | HQ002397 | HQ002828 | HQ001915 | HQ000365 | HQ000491 | HQ001225 |
| 779 ^b^ | HM745709 | HM745899 | HQ002096 | HM745823 | HQ000492 | HM745633 |
| 780 ^b^ | HQ002396 | HQ002834 | HQ002097 | HQ000366 | HQ000493 | HQ001226 |
| 785 ^b^ | HQ002392 | HQ002818 | HQ002098 | HQ000367 | HQ000494 | HQ001227 |
| 786 ^b^ | HQ002407 | HQ002821 | HQ002099 | HQ000368 | HQ000495 | HQ001228 |
| 787 ^b^ | HQ002386 | HQ002817 | HQ002100 | HQ000369 | HQ000496 | HQ001229 |
| 788 ^a^ | HQ002394 | HQ002819 | HQ002101 | HQ000370 | HQ000497 | HQ001230 |
| 790 ^b^ | HM745710 | HM745900 | HQ001916 | HM745824 | HQ000498 | HM745634 |
| 791 ^b^ | HQ002412 | HQ002820 | HQ001917 | HQ000371 | HQ000499 | HQ001231 |
| 792 ^b^ | HQ002382 | HQ002822 | HQ002102 | HQ000372 | HQ000500 | HQ001232 |
| 793 ^b^ | HQ002398 | HQ002832 | HQ001918 | HQ000373 | HQ000501 | HQ001233 |
| 796 ^b^ | HQ002404 | HQ002837 | HQ001919 | HQ000374 | HQ000502 | HQ001234 |
| 797 ^b^ | HM745711 | HM745901 | HQ002103 | HM745825 | HQ000503 | HM745635 |
| 798 ^b^ | HQ002419 | HQ002824 | HQ002104 | HQ000375 | HQ000504 | HQ001235 |
| 799 ^b^ | HQ002391 | HQ002823 | HQ001920 | HQ000376 | HQ000505 | HQ001236 |

^a^ Strains that produce B and G aflatoxins

^b^ Strains that produce B aflatoxins only

IC numbers for U.S.A. strains (1154-1178; 1228)

IC numbers for Argentina strains (476-479)

IC numbers for Australia strains (720-799)

IC numbers for Benin strains (1112-1153)
