# Supplementary material for: Global population structure and adaptive evolution of aflatoxin‐producing fungi
Source: Ecol Evol. 2017 Sep 30;7(21):9179–91. doi: 10.1002/ece3.3464 (PMC5677503; doi:10.1002/ece3.3464)
Supplement: Supplementary file 16 [file ECE3-7-9179-s016.docx]

Table S4. GenBank accession numbers for *A. parasiticus* sequences used in this study

| **IC Strain** | ***W/X*** | ***M/N*** | ***MAT*** | ***amdS*** | ***mfs*** | ***trpC*** |
| --- | --- | --- | --- | --- | --- | --- |
| 1 | HM354778 | HM353306 | HQ001490 | HM354439 | HQ000836 | HM353306 |
| 2 | DQ390832 | DQ391040 | HQ001499 | DQ391094 | HQ000845 | DQ391156 |
| 3 |  |  |  |  | KX853146 |  |
| 4 |  |  | HQ001528 |  |  |  |
| 5 | HM354872 | HM353400 | HQ001587 | HM354533 | HQ000950 | HM353852 |
| 6 |  |  |  |  | KX853147 |  |
| 7 | HM354879 | HM353407 | HQ001596 | HM354540 | HQ000969 | HM353859 |
| 8 | HM354916 | HM353443 | HQ001616 | HM354576 | HQ001010 | HM353895 |
| 9 |  |  |  |  | KX853148 |  |
| 10 | HM354771 | HM353299 | HQ001445 | HM354432 | HQ000801 | HM353752 |
| 11 | DQ390831 | DQ391032 | HQ001457 | DQ391098 | HQ000809 | DQ391143 |
| 12 | HM354772 | HM353300 | HQ001467 | HM354433 | HQ000815 | HM353753 |
| 13 | HM354773 | HM353301 | HQ001478 | HM354434 | HQ000825 | HM353754 |
| 14 | HM354774 | HM353302 | HQ001484 | HM354435 | HQ000831 | HM353755 |
| 15 | DQ390826 | DQ391034 | HQ001485 | DQ391097 | HQ000832 | DQ391140 |
| 16 |  |  | HQ001486 |  |  |  |
| 17 | HM354775 | HM353303 | HQ001487 | HM354436 | HQ000833 | HM353756 |
| 18 | HM354776 | HM353304 | HQ001488 | HM354437 | HQ000834 | HM353757 |
| 19 | HM354777 | HM353305 | HQ001489 | HM354438 | HQ000835 | HM353758 |
| 21 | HM354779 | HM353307 | HQ001491 | HM354440 | HQ000837 | HM353760 |
| 22 | HM354780 | HM353308 | HQ001492 | HM354441 | HQ000838 | HM353761 |
| 23 | HM354781 | HM353309 | HQ001493 | HM354442 | HQ000839 | HM353762 |
| 24 | HM354782 | HM353310 | HQ001494 | HM354443 | HQ000840 | HM353763 |
| 25 | DQ390825 | DQ391039 | KX853136 | DQ391104 | HQ000841 | DQ391149 |
| 26 | HM354783 | HM353311 | HQ001495 | HM354444 | HQ000842 | HM353764 |
| 27 | HM354784 | HM353312 | HQ001496 | HM354445 | HQ000843 | HM353765 |
| 28 |  |  | HQ001497 |  |  |  |
| 29 | HM354785 | HM353313 | HQ001498 | HM354446 | HQ000844 | HM353766 |
| 30 |  |  | HQ001500 |  | KX853149 |  |
| 32 | HM354786 | HM353314 | HQ001501 | HM354447 | HQ000859 | HM353767 |
| 33_M1^a^ | HM354787 | HM353315 | HQ001502 | HM354448 | HQ000862 | HM353768 |
| 33_M2 ^a^ |  |  | HQ001999 |  |  |  |
| 34 | HM354788 | HM353316 | HQ001503 | HM354449 | HQ000863 | HM353769 |
| 35 | HM354789 | HM353317 | HQ001504 | HM354450 | HQ000864 | HM353770 |
| 36 | HM354790 | HM353318 | HQ001505 | HM354451 | HQ000865 | HM353771 |
| 37 | HM354791 | HM353319 | HQ001506 | HM354452 | HQ000866 | HM353772 |
| 38 | DQ390827 | DQ391038 | HQ002000 | DQ391095 | HQ000867 | DQ391142 |
| 39 | HM354792 | HM353320 | KX853137 | HM354453 | HQ000868 | HM353773 |
| 40 | HM354793 | HM353321 | HQ001507 | HM354454 | HQ000869 | HM353774 |
| 41 |  |  | HQ001508 |  |  |  |
| 42 | HM354794 | HM353322 | HQ001509 | HM354455 | HQ000870 | HM353775 |
| 44 | HM354795 | HM353323 | HQ001510 | HM354456 | HQ000871 | HM353776 |
| 45 |  |  | HQ001511 |  | KX853150 |  |
| 46 | HM354796 | HM353324 | HQ001512 | HM354457 | HQ000872 | HM353777 |
| 47 | HM354797 | HM353325 | HQ001513 | HM354458 | HQ000873 | HM353778 |
| 48 | HM354804 | HM353332 | HQ001520 | HM354465 | HQ000880 | HM353785 |
| 49 | HM354812 | HM353340 | HQ001527 | HM354473 | HQ000888 | HM353792 |
| 50 | HM354821 | HM353359 | HQ001537 | HM354492 | HQ000897 | HM353811 |
| 51 | HM354831 | HM353335 | HQ001547 | HM354468 | HQ000907 | HM353788 |
| 52 | HM354841 | HM353369 | HQ001556 | HM354502 | HQ000917 | HM353821 |
| 53_M1 ^a^ | HM354852 | HM353380 | HQ001567 | HM354513 | HQ000928 | HM353832 |
| 53_M2 ^a^ |  |  | HQ002003 |  |  |  |
| 54 | HM354863 | HM353391 | HQ001578 | HM354524 | HQ000939 | HM353843 |
| 55 | HM354870 | HM353398 | HQ001585 | HM354531 | HQ000946 | HM353850 |
| 56 | DQ390839 | DQ391036 | HQ001586 | DQ391112 | HQ000947 | DQ391137 |
| 58 | HM354871 | HM353399 | HQ002004 | HM354532 | HQ000948 | HM353851 |
| 59 | DQ390837 | DQ391035 | HQ002005 | DQ391106 | HQ000949 | DQ391138 |
| 60 | HM354873 | HM353401 | HQ002006 | HM354534 | HQ000951 | HM353853 |
| 61 | DQ390846 | DQ391017 | HQ001588 | DQ391102 | HQ000952 | DQ391148 |
| 62 | HM354874 | HM353402 | KX853138 | HM354535 | HQ000953 | HM353854 |
| 63 | HM354875 | HM353403 | KX853139 | HM354536 | HQ000954 | HM353855 |
| 64 | HM354876 | HM353404 | KX853140 | HM354537 | HQ000955 | HM353856 |
| 65_M1 ^a^ | DQ390848 | DQ391018 | HQ001589 | DQ391109 | HQ000956 | DQ391150 |
| 65_M2 ^a^ |  |  | KX853983 |  |  |  |
| 66 | HM354877 | HM353405 | HQ002007 | HM354538 | HQ000957 | HM353857 |
| 67_M1 ^a^ | DQ390844 | DQ391019 | HQ001590 | DQ391101 | HQ000958 | DQ391141 |
| 67_M2 ^a^ |  |  | KX853984 |  |  |  |
| 68 | HM354878 | HM353406 | HQ001591 | HM354539 | HQ000959 | HM353858 |
| 69 | DQ390840 | DQ391020 | HQ002008 | DQ391096 | HQ000960 | DQ391145 |
| 70 | DQ390838 | DQ391021 | KX853141 | DQ391108 | HQ000961 | DQ391152 |
| 71_M1 ^a^ | DQ390843 | DQ391022 | HQ001592 | DQ391090 | HQ000962 | DQ391154 |
| 71_M2 ^a^ |  |  | KX853985 |  |  |  |
| 72_M1 ^a^ | DQ390845 | DQ391023 | HQ002009 | DQ391103 | HQ000963 | DQ391160 |
| 72_M2 ^a^ |  |  | KX853986 |  |  |  |
| 73 | DQ390829 | DQ391024 | KX853142 | DQ391100 | HQ000964 | DQ391146 |
| 74 | DQ390834 | DQ391025 | KX853143 | DQ391089 | HQ000965 | DQ391153 |
| 75 | DQ390847 | DQ391028 | KX853144 | DQ391111 | HQ000966 | DQ391155 |
| 76 | DQ390842 | DQ391029 | KX853987 | DQ391110 | HQ000967 | DQ391151 |
| 77 | DQ390841 | HQ002721 | HQ001593 | HQ000138 | HQ000968 | DQ391144 |
| 78 |  |  | HQ001594 |  |  | DQ391159 |
| 79 |  |  | HQ001595 |  |  |  |
| 80 |  |  | HQ001597 |  |  |  |
| 81 | HQ002499 | HQ002722 | HQ001600 | HQ000137 | HQ000982 | HQ001284 |
| 82 |  |  |  |  | KX853151 |  |
| 83 | HQ002518 | HQ002723 | HQ001605 | HQ000143 | HQ000992 | HQ001285 |
| 84 | HQ002541 | HQ002724 | HQ001607 | HQ000142 | HQ000996 | HQ001286 |
| 85 |  |  |  |  | KX853152 |  |
| 86 | HQ002540 | HQ002725 | HQ001611 | HQ000140 | HQ001005 | HQ001287 |
| 87 |  |  |  |  | KX853153 |  |
| 88 | HQ002542 | HQ002726 | HQ001614 | HQ000168 | HQ001009 | HQ001288 |
| 89 |  |  | HQ001615 |  |  |  |
| 90 |  |  | HQ001620 |  |  |  |
| 91 |  |  | HQ001621 |  |  |  |
| 92 |  |  | HQ001626 |  |  |  |
| 93 |  |  | HQ001627 |  |  |  |
| 94 |  |  | HQ001628 |  |  |  |
| 95 | HQ002496 | HQ002727 | HQ001629 | HQ000169 | HQ001033 | HQ001311 |
| 96 | HQ002495 | HQ002728 | HQ001630 | HQ000170 | HQ001034 | HQ001312 |
| 97 | HQ002503 | HQ002729 | HQ001631 | HQ000174 | HQ001035 | HQ001313 |
| 98 | HQ002504 | DQ391026 | HQ001632 | HQ000173 | HQ001036 | DQ391158 |
| 99 | HQ002530 | HQ002730 | HQ001633 | HQ000153 | HQ001037 | HQ001314 |
| 100 | HQ002550 | HQ002731 | HQ001435 | HQ000146 | HQ000793 | HQ001237 |
| 101 | HQ002476 | HQ002732 | HQ001436 | HQ000145 | HQ000794 | HQ001238 |
| 102 | HQ002517 | HQ002733 | HQ001437 | HQ000154 | HQ000795 | HQ001239 |
| 103 |  |  | HQ001438 |  |  |  |
| 104 |  |  | HQ001439 |  |  |  |
| 105 | HQ002510 | HQ002734 | HQ001440 | HQ000112 | HQ000796 | HQ001240 |
| 106 | HQ002512 | HQ002735 | HQ001441 | HQ000159 | HQ000797 | HQ001241 |
| 107 | HQ002490 | HQ002736 | HQ001442 | HQ000115 | HQ000798 | HQ001242 |
| 108 | HQ002481 | HQ002737 | HQ001443 | HQ000116 | HQ000799 | HQ001243 |
| 109 | HQ002483 | HQ002738 | HQ001444 | HQ000147 | HQ000800 | HQ001244 |
| 110 | HQ002487 | HQ002739 | HQ001447 | HQ000114 | HQ000803 | HQ001246 |
| 111 | HQ002522 | HQ002740 | HQ001448 | HQ000113 | HQ000804 | HQ001247 |
| 112 | HQ002523 | HQ002741 | HQ001449 | HQ000095 | HQ000805 | HQ001248 |
| 113 |  |  | HQ001450 |  | KX853154 |  |
| 114 |  |  | HQ001451 |  | KX853155 |  |
| 115 | HQ002513 | DQ391031 | HQ001452 | HQ000096 | HQ000806 | DQ391157 |
| 116 |  |  | HQ001453 |  |  |  |
| 117 |  |  | HQ001454 |  |  |  |
| 118 | HQ002559 | HQ002742 | HQ001455 | HQ000101 | HQ000807 | HQ001249 |
| 119 | HQ002488 | HQ002743 | HQ001456 | HQ000105 | HQ000808 | HQ001250 |
| 120 |  |  | HQ001458 |  | KX853156 |  |
| 121 |  |  | HQ001459 |  | KX853157 |  |
| 122 |  |  |  |  | KX853158 |  |
| 123 | HQ002489 | DQ391033 | HQ001460 | HQ000100 | HQ000810 | DQ391147 |
| 124 |  |  | HQ001461 |  | KX853159 |  |
| 125 | HQ002484 | HQ002744 | HQ001462 | HQ000099 | HQ000811 | HQ001251 |
| 126 | HQ002508 | HQ002745 | HQ001463 | HQ000097 | HQ000812 | HQ001252 |
| 127 |  |  | HQ001464 |  | KX853160 |  |
| 128 | HQ002485 | HQ002746 | HQ001465 | HQ000098 | HQ000813 | HQ001253 |
| 129 | HQ002509 | HQ002747 | HQ001466 | HQ000166 | HQ000814 | HQ001254 |
| 130 | HQ002515 | HQ002748 | HQ001468 | HQ000165 | HQ000816 | HQ001255 |
| 131 | HQ002486 | HQ002749 | HQ001469 | HQ000162 | HQ000817 | HQ001256 |
| 132 |  |  | HQ001470 |  | KX853161 |  |
| 133 | HQ002482 | HQ002750 | HQ001471 | HQ000163 | HQ000818 | HQ001257 |
| 134 | HQ002557 | HQ002751 | HQ001472 | HQ000164 | HQ000819 | HQ001258 |
| 135 | HQ002558 | HQ002752 | HQ001473 | HQ000167 | HQ000820 | HQ001259 |
| 136 | HQ002493 | HQ002753 | HQ001474 | HQ000161 | HQ000821 | HQ001260 |
| 137 | HQ002477 | HQ002754 | HQ001475 | HQ000160 | HQ000822 | HQ001261 |
| 138 | HQ002505 | HQ002755 | HQ001476 | HQ000109 | HQ000823 | HQ001262 |
| 139 | HQ002480 | HQ002756 | HQ001477 | HQ000110 | HQ000824 | HQ001263 |
| 140 | HQ002547 | HQ002757 | HQ001479 | HQ000111 | HQ000826 | HQ001264 |
| 141 | HQ002494 | HQ002758 | HQ001480 | HQ000108 | HQ000827 | HQ001265 |
| 142 | HQ002498 | HQ002759 | HQ001481 | HQ000107 | HQ000828 | HQ001266 |
| 143 | HQ002506 | HQ002760 | HQ001482 | HQ000158 | HQ000829 | HQ001267 |
| 144 | HQ002548 | HQ002761 | HQ001483 | HQ000141 | HQ000830 | HQ001268 |
| 317 | HQ002521 | HQ002762 | HQ001996 | HQ000120 | HQ000846 | HQ001269 |
| 318 | HQ002554 | HQ002763 | KX853988 | HQ000121 | HQ000847 | HQ001270 |
| 319 | HQ002553 | HQ002764 | KX853989 | HQ000106 | HQ000848 | HQ001271 |
| 320 | HQ002552 | HQ002765 | KX853990 | HQ000104 | HQ000849 | HQ001272 |
| 321 | HQ002497 | HQ002766 | KX853991 | HQ000128 | HQ000850 | HQ001273 |
| 322 | HQ002555 | HQ002767 | KX853992 | HQ000102 | HQ000851 | HQ001274 |
| 323 | HQ002551 | HQ002768 | KX853993 | HQ000103 | HQ000852 | HQ001275 |
| 324 | HQ002549 | HQ002769 | KX853994 | HQ000119 | HQ000853 | HQ001276 |
| 325 | HQ002500 | HQ002770 | KX853995 | HQ000117 | HQ000854 | HQ001277 |
| 326 | HQ002507 | HQ002771 | KX853996 | HQ000118 | HQ000855 | HQ001278 |
| 327 | HQ002514 | HQ002772 | KX853997 | HQ000139 | HQ000856 | HQ001279 |
| 328 | HQ002478 | HQ002773 | HQ001997 | HQ000176 | HQ000857 | HQ001280 |
| 329 | HQ002492 | HQ002774 | KX853998 | HQ000172 | HQ000858 | HQ001281 |
| 330 | HQ002491 | HQ002775 | HQ001998 | HQ000148 | HQ000860 | HQ001282 |
| 331 | HQ002520 | HQ002776 | KX853999 | HQ000149 | HQ000861 | HQ001283 |
| 480 | HM354798 | HM353326 | HQ001514 | HM354459 | HQ000874 | HM353779 |
| 484 | HM354799 | HM353327 | HQ001515 | HM354460 | HQ000875 | HM353780 |
| 485 | HM354800 | HM353328 | HQ001516 | HM354461 | HQ000876 | HM353781 |
| 486 | HM354801 | HM353329 | HQ001517 | HM354462 | HQ000877 | HM353782 |
| 487 | HM354802 | HM353330 | HQ001518 | HM354463 | HQ000878 | HM353783 |
| 489 | HM354803 | HM353331 | HQ001519 | HM354464 | HQ000879 | HM353784 |
| 490 | HM354805 | HM353333 | HQ001521 | HM354466 | HQ000881 | HM353786 |
| 491 | HM354806 | HM353334 | HQ001522 | HM354467 | HQ000882 |  |
| 494 | HM354807 | HM353335 | HQ002001 | HM354468 | HQ000883 | HM353787 |
| 495 | HM354808 | HM353336 | HQ001523 | HM354469 | HQ000884 | HM353788 |
| 496 | HM354809 | HM353337 | HQ001524 | HM354470 | HQ000885 | HM353789 |
| 497 | HM354810 | HM353338 | HQ001525 | HM354471 | HQ000886 | HM353790 |
| 499 | HM354811 | HM353339 | HQ001526 | HM354472 | HQ000887 | HM353791 |
| 500 | HM354813 | HM353341 | HQ001529 | HM354474 | HQ000889 | HM353793 |
| 502 | HM354814 | HM353342 | HQ001530 | HM354475 | HQ000890 | HM353794 |
| 504 | HM354815 | HM353343 | HQ001531 | HM354476 | HQ000891 | HM353795 |
| 505 | HM354816 | HM353344 | HQ001532 | HM354477 | HQ000892 | HM353796 |
| 506 | HM354817 | HM353345 | HQ001533 | HM354478 | HQ000893 | HM353797 |
| 507 | HM354818 | HM353346 | HQ001534 | HM354479 | HQ000894 | HM353798 |
| 508 | HM354819 | HM353347 | HQ001535 | HM354480 | HQ000895 | HM353799 |
| 509 | HM354820 | HM353348 | HQ001536 | HM354481 | HQ000896 | HM353800 |
| 510 | HM354822 | HM353350 | HQ001538 | HM354483 | HQ000898 | HM353802 |
| 511 | HM354823 | HM353351 | HQ001539 | HM354484 | HQ000899 | HM353803 |
| 512 | HM354824 | HM353352 | HQ001540 | HM354485 | HQ000900 | HM353804 |
| 513 | HM354825 | HM353353 | HQ001541 | HM354486 | HQ000901 | HM353805 |
| 514 | HM354826 | HM353354 | HQ001542 | HM354487 | HQ000902 | HM353806 |
| 516 | HM354827 | HM353355 | HQ001543 | HM354488 | HQ000903 | HM353807 |
| 517 | HM354828 | HM353356 | HQ001544 | HM354489 | HQ000904 | HM353808 |
| 518 | HM354829 | HM353357 | HQ001545 | HM354490 | HQ000905 | HM353809 |
| 519 | HM354830 | HM353358 | HQ001546 | HM354491 | HQ000906 | HM353810 |
| 520 | HM354831 | HM353359 | HQ001547 | HM354492 | HQ000907 | HM353811 |
| 521 | HM354833 | HM353361 | HQ001549 | HM354494 | HQ000909 | HM353813 |
| 522 | HM354834 | HM353362 | HQ001550 | HM354495 | HQ000910 | HM353814 |
| 523 | HM354835 | HM353363 | HQ001551 | HM354496 | HQ000911 | HM353815 |
| 524 | HM354836 | HM353364 | HQ001552 | HM354497 | HQ000912 | HM353816 |
| 525 | HM354837 | HM353365 | HQ001553 | HM354498 | HQ000913 | HM353817 |
| 526 | HM354838 | HM353366 | HQ002002 | HM354499 | HQ000914 | HM353818 |
| 528 | HM354839 | HM353367 | HQ001554 | HM354500 | HQ000915 | HM353819 |
| 529 | HM354840 | HM353368 | HQ001555 | HM354501 | HQ000916 | HM353820 |
| 530 | HM354842 | HM353370 | HQ001557 | HM354503 | HQ000918 | HM353822 |
| 531 | HM354843 | HM353371 | HQ001558 | HM354504 | HQ000919 | HM353823 |
| 532 | HM354844 | HM353372 | HQ001559 | HM354505 | HQ000920 | HM353824 |
| 533 | HM354845 | HM353373 | HQ001560 | HM354506 | HQ000921 | HM353825 |
| 534 | HM354846 | HM353374 | HQ001561 | HM354507 | HQ000922 | HM353826 |
| 535 | HM354847 | HM353375 | HQ001562 | HM354508 | HQ000923 | HM353827 |
| 536 | HM354848 | HM353376 | HQ001563 | HM354509 | HQ000924 | HM353828 |
| 537 | HM354849 | HM353377 | HQ001564 | HM354510 | HQ000925 | HM353829 |
| 538 | HM354850 | HM353378 | HQ001565 | HM354511 | HQ000926 | HM353830 |
| 539 | HM354851 | HM353379 | HQ001566 | HM354512 | HQ000927 | HM353831 |
| 540 | HM354853 | HM353381 | HQ001568 | HM354514 | HQ000929 | HM353833 |
| 541 | HM354854 | HM353382 | HQ001569 | HM354515 | HQ000930 | HM353834 |
| 542 | HM354855 | HM353383 | HQ001570 | HM354516 | HQ000931 | HM353835 |
| 543 | HM354856 | HM353384 | HQ001571 | HM354517 | HQ000932 | HM353836 |
| 544 | HM354857 | HM353385 | HQ001572 | HM354518 | HQ000933 | HM353837 |
| 545 | HM354858 | HM353386 | HQ001573 | HM354519 | HQ000934 | HM353838 |
| 546 | HM354859 | HM353387 | HQ001574 | HM354520 | HQ000935 | HM353839 |
| 547 | HM354860 | HM353388 | HQ001575 | HM354521 | HQ000936 | HM353840 |
| 548 | HM354861 | HM353389 | HQ001576 | HM354522 | HQ000937 | HM353841 |
| 549 | HM354862 | HM353390 | HQ001577 | HM354523 | HQ000938 | HM353842 |
| 551 | HM354864 | HM353392 | HQ001579 | HM354525 | HQ000940 | HM353844 |
| 552 | HM354865 | HM353393 | HQ001580 | HM354526 | HQ000941 | HM353845 |
| 553 | HM354866 | HM353394 | HQ001581 | HM354527 | HQ000942 | HM353846 |
| 554 | HM354867 | HM353395 | HQ001582 | HM354528 | HQ000943 | HM353847 |
| 555 | HM354868 | HM353396 | HQ001583 | HM354529 | HQ000944 | HM353848 |
| 556 | HM354869 | HM353397 | HQ001584 | HM354530 | HQ000945 | HM353849 |
| 800 | HM354880 | HM353408 | HQ002010 | HM354541 | HQ000970 | HM353860 |
| 801 | HM354881 | HM353409 | HQ002011 | HM354542 | HQ000971 | HM353861 |
| 804 | HM354882 | HM353410 | HQ002012 | HM354543 | HQ000972 | HM353862 |
| 805 | HM354883 | HM353411 | HQ002013 | HM354544 | HQ000973 | HM353863 |
| 806 | HM354884 | HM353412 | HQ002014 | HM354545 | HQ000974 | HM353864 |
| 807 | HM354885 | HM353413 | HQ002015 | HM354546 | HQ000975 | HM353865 |
| 808 | HM354886 | HM353414 | HQ002016 | HM354547 | HQ000976 | HM353866 |
| 809 | HM354887 | HM353415 | HQ002017 | HM354548 | HQ000977 | HM353867 |
| 811 | HM354888 | HM353416 | HQ001598 | HM354549 | HQ000978 | HM353868 |
| 813 | HM354889 | HM353417 | HQ001599 | HM354550 | HQ000979 | HM353869 |
| 814 | HM354890 | HM353418 | HQ002018 | HM354551 | HQ000980 | HM353870 |
| 816 | HM354891 | HM353419 | HQ002019 | HM354552 | HQ000981 | HM353871 |
| 822 | HM354892 | HM353420 | HQ002020 | HM354553 | HQ000983 | HM353872 |
| 824 | HM354893 | HM353421 | HQ001601 | HM354554 | HQ000984 | HM353873 |
| 825 | HM354894 | HM353422 | HQ001602 | HM354555 | HQ000985 | HM353874 |
| 828 | HM354895 | HM353423 | HQ002021 | HM354556 | HQ000986 | HM353875 |
| 832 | HM354896 | HM353424 | HQ002022 | HM354557 | HQ000987 | HM353876 |
| 835 | HM354897 | HM353425 | HQ001603 | HM354558 | HQ000988 | HM353877 |
| 836 | HM354898 | HM353426 | HQ001604 | HM354559 | HQ000989 | HM353878 |
| 837 | HM354899 | HM353427 | HQ002023 | HM354560 | HQ000990 | HM353879 |
| 839 | HM354900 | HM353428 | HQ002024 | HM354561 | HQ000991 | HM353880 |
| 840 | HM354901 | HM353429 | HQ002025 | HM354562 | HQ000993 | HM353881 |
| 844 | HM354902 | HM353430 | HQ001606 | HM354563 | HQ000994 | HM353882 |
| 848 | HM354903 | HM353431 | HQ002026 | HM354564 | HQ000995 | HM353883 |
| 851 | HM354904 | HM353432 | HQ001608 | HM354565 | HQ000997 | HM353884 |
| 853 | HM354905 | HM353433 | HQ002027 | HM354566 | HQ000998 | HM353885 |
| 854 | HM354906 | HM353434 | HQ002028 | HM354567 | HQ000999 | HM353886 |
| 860 | HM354907 | HM353435 | HQ002029 | HM354568 | HQ001000 | HM353887 |
| 863 | HM354908 | HM353436 | HQ002030 | HM354569 | HQ001001 | HM353888 |
| 864 | HM354909 | HM353437 | HQ001609 | HM354570 | HQ001002 | HM353889 |
| 867 | HM354910 | HM353438 | HQ002031 | HM354571 | HQ001003 | HM353890 |
| 868 | HM354911 | HM353439 | HQ001610 | HM354572 | HQ001004 | HM353891 |
| 872 | HM354913 | HM353440 | HQ001612 | HM354573 | HQ001006 | HM353892 |
| 875 | HM354914 | HM353441 | HQ001613 | HM354574 | HQ001007 | HM353893 |
| 876 | HM354915 | HM353442 | HQ002032 | HM354575 | HQ001008 | HM353894 |
| 905 | HQ002533 | HQ002777 | HQ001617 | HQ000157 | HQ001011 | HQ001289 |
| 906 | HQ002543 | HQ002778 | HQ001618 | HQ000151 | HQ001012 | HQ001290 |
| 907 | HQ002544 | HQ002779 | HQ001619 | HQ000150 | HQ001013 | HQ001291 |
| 908 | HQ002536 | HQ002780 | HQ002033 | HQ000122 | HQ001014 | HQ001292 |
| 909 | HQ002529 | HQ002781 | HQ002034 | HQ000123 | HQ001015 | HQ001293 |
| 910 | HQ002528 | HQ002782 | HQ002035 | HQ000124 | HQ001016 | HQ001294 |
| 911 | HQ002527 | HQ002783 | HQ002036 | HQ000171 | HQ001017 | HQ001295 |
| 912 | HQ002524 | HQ002784 | HQ002037 | HQ000125 | HQ001018 | HQ001296 |
| 913 | HQ002525 | HQ002785 | HQ002038 | HQ000126 | HQ001019 | HQ001297 |
| 915 | HQ002526 | HQ002786 | HQ002039 | HQ000127 | HQ001020 | HQ001298 |
| 916 | HQ002537 | HQ002787 | HQ002040 | HQ000129 | HQ001021 | HQ001299 |
| 917 | HQ002538 | HQ002788 | HQ002041 | HQ000130 | HQ001022 | HQ001300 |
| 918 | HQ002539 | HQ002789 | HQ002042 | HQ000134 | HQ001023 | HQ001301 |
| 919 | HQ002535 | HQ002790 | HQ002043 | HQ000133 | HQ001024 | HQ001302 |
| 920 | HQ002545 | HQ002791 | HQ001622 | HQ000152 | HQ001025 | HQ001303 |
| 921 | HQ002519 | HQ002792 | HQ001623 | HQ000156 | HQ001026 | HQ001304 |
| 922 | HQ002532 | HQ002793 | HQ001624 | HQ000155 | HQ001027 | HQ001305 |
| 923 | HQ002534 | HQ002794 | HQ002044 | HQ000135 | HQ001028 | HQ001306 |
| 924 | HQ002501 | HQ002795 | HQ002045 | HQ000136 | HQ001029 | HQ001307 |
| 925 | HQ002531 | HQ002796 | HQ001625 | HQ000144 | HQ001030 | HQ001308 |
| 926 | HQ002502 | HQ002797 | HQ002046 | HQ000132 | HQ001031 | HQ001309 |
| 927 | HQ002546 | HQ002798 | HQ002047 | HQ000131 | HQ001032 | HQ001310 |
| 1107 | HQ002479 | HQ002799 | HQ001446 | HQ000175 | HQ000802 | HQ001245 |

^a^ Strains that amplified both mating-type idiomorphs

IC numbers for U.S.A. strains (1-144; 317-331; 905-927)

IC numbers for Argentina strains (480-559)

IC numbers for Australia strains (800-876)

IC number for SU-1 strain (1107)
