# Supplementary material for: Global population structure and adaptive evolution of aflatoxin‐producing fungi
Source: Ecol Evol. 2017 Sep 30;7(21):9179–91. doi: 10.1002/ece3.3464 (PMC5677503; doi:10.1002/ece3.3464)
Supplement: Supplementary file 17 [file ECE3-7-9179-s017.docx]

Table S5. GenBank accession numbers for *A. caelatus* sequences used in this study

| **IC Strain** | ***W/X*** | ***MAT*** | ***amdS*** | ***mfs*** | ***trpC*** |
| --- | --- | --- | --- | --- | --- |
| 1559 | HQ002632 | HQ001939 | HQ000377 | HQ001071 | HQ001326 |
| 1560 | HQ002633 | HQ001940 | HQ000378 | HQ001072 | HQ001327 |
| 1561 | HQ002634 | HQ001941 | HQ000379 | HQ001073 |  |
| 1562 | HQ002635 | HQ001942 | HQ000380 | HQ001074 | HQ001328 |
| 1563 | HQ002636 | HQ001943 | HQ000381 | HQ001075 |  |
| 1564 | HQ002637 | HQ001944 | HQ000382 | HQ001076 | HQ001329 |
| 1565 |  | HQ001945 | HQ000383 | HQ001077 | HQ001330 |
| 1566 | HQ002638 | HQ001946 | HQ000384 | HQ001078 | HQ001331 |
| 1567 | HQ002639 | HQ001947 |  | HQ001079 |  |
| 1568 |  | HQ001948 |  | HQ001080 | HQ001332 |
| 1569 | HQ002640 | HQ001949 | HQ000385 | HQ001081 | HQ001333 |
| 1570 |  | HQ001950 |  | HQ001082 | HQ001334 |
| 1571 | HQ002641 | HQ001951 | HQ000386 | HQ001083 |  |
| 1572 | HQ002642 | HQ001952 | HQ000387 | HQ001084 | HQ001335 |
| 1573 | HQ002643 | HQ002105 | HQ000388 | HQ001085 | HQ001336 |
| 1574 | HQ002644 |  | HQ000389 | HQ001086 | HQ001337 |
| 1575 | HQ002645 | HQ001953 | HQ000390 | HQ001087 |  |
| 1576 |  | HQ001954 |  |  | HQ001338 |
| 1577 | HQ002646 | HQ001955 | HQ000391 | HQ001088 | HQ001339 |
| 1578 | HQ002647 | HQ001956 |  | HQ001089 |  |
| 1579 |  | HQ001957 |  | HQ001090 |  |
| 1580 | HQ002648 | HQ002106 | HQ000392 | HQ001091 | HQ001340 |
| 1581 | HQ002649 | HQ002107 | HQ000393 | HQ001092 | HQ001341 |
| 1582 | HQ002650 | HQ002108 |  | HQ001093 |  |
| 1583 |  | HQ001958 |  | HQ001094 |  |
| 1584 | HQ002651 | HQ002109 | HQ000394 | HQ001095 | HQ001342 |
| 1585 | HQ002652 | HQ001959 |  | HQ001096 |  |
| 1586 | HQ002653 | HQ001960 |  | HQ001097 |  |
| 1587 | HQ002654 | HQ001961 |  | HQ001098 |  |
| 1588 |  | HQ001962 |  |  |  |
| 1589 |  | HQ001963 |  |  |  |
| 162 | HQ002655 | HQ001964 | HQ000395 | HQ001099 | HQ001343 |
| 561 | HQ002656 | HQ001965 |  |  | HQ001344 |
| 564 |  |  |  |  | HQ001345 |
| 565 | HQ002657 | HQ001966 | HQ000396 | HQ001100 | HQ001346 |
| 566 | HQ002658 | HQ002110 |  |  | HQ001347 |
| 567 | HQ002659 | HQ002111 | HQ000397 | HQ001101 | HQ001348 |
| 568 | HQ002660 | HQ001967 | HQ000398 | HQ001102 | HQ001349 |
| 569 |  | HQ001968 |  | HQ001103 | HQ001350 |
| 570 | HQ002661 | HQ002112 | HQ000399 | HQ001104 | HQ001351 |
| 571 | HQ002662 |  |  |  | HQ001352 |
| 572 |  |  |  |  | HQ001353 |
| 573 |  |  |  |  | HQ001354 |
| 574 |  |  |  |  | HQ001355 |
| 577 |  | HQ001969 | HQ000400 |  | HQ001356 |
| 578 |  |  |  |  | HQ001357 |
| 580 | HQ002663 | HQ001970 | HQ000401 |  | HQ001358 |
| 582 |  | HQ001971 |  | HQ001105 | HQ001359 |
| 588 |  |  |  |  | HQ001360 |
| 590 |  |  |  |  | HQ001361 |
| 591 | HQ002664 | HQ002113 | HQ000402 | HQ001106 | HQ001362 |
| 596 |  | HQ002114 |  | HQ001107 |  |
| 598 | HQ002665 | HQ001972 | HQ000403 | HQ001108 | HQ001363 |
| 599 |  | HQ001973 | HQ000404 |  | HQ001364 |
| 600 |  | HQ001974 |  |  | HQ001365 |
| 601 |  | HQ001975 | HQ000405 | HQ001109 |  |
| 602 |  | HQ001976 |  |  |  |
| 603 |  | HQ001977 |  |  | HQ001366 |
| 604 |  |  |  |  | HQ001367 |
| 606 |  | HQ001979 |  |  |  |
| 610 |  | HQ001980 |  |  |  |
| 611 | HQ002666 | HQ002115 | HQ000406 | HQ001110 | HQ001368 |
| 613 |  | HQ002116 | HQ000407 | HQ001111 | HQ001369 |
| 614 |  | HQ001981 |  |  |  |
| 615 |  |  |  |  | HQ001370 |
| 616 |  | HQ001982 |  |  | HQ001371 |
| 617 | HQ002667 | HQ001983 | HQ000408 |  | HQ001372 |
| 618 | HQ002668 | HQ002117 | HQ000409 | HQ001112 | HQ001373 |
| 619 |  | HQ001984 |  |  |  |
| 620 |  | HQ001985 |  |  |  |
| 621 |  | HQ002118 |  |  |  |
| 622 |  | KX853145 |  |  |  |
| 623 |  | HQ002119 |  |  |  |
| 624 | HQ002669 | HQ002120 | HQ000410 | HQ001113 | HQ001374 |
| 626 | HQ002670 | HQ001987 | HQ000411 | HQ001114 | HQ001375 |
| 627 |  | HQ001988 |  |  |  |
| 628 |  | HQ001989 |  |  |  |
| 629 |  | HQ001990 |  |  |  |
| 630 |  | HQ001991 | HQ000412 | HQ001115 | HQ001376 |
| 631 |  | HQ001992 |  |  |  |
| 632 |  | HQ001993 |  |  |  |
| 633 |  | HQ002121 | HQ000413 |  | HQ001377 |
| 634 |  | HQ001994 | HQ000414 |  | HQ001378 |
| 635 |  | HQ002122 |  |  |  |
| 636 |  | HQ002123 | HQ000415 |  | HQ001379 |
| 639 | HQ002671 | HQ001995 | HQ000416 | HQ001116 | HQ001380 |

IC numbers for U.S.A. strains (1559-1589; 162)

IC numbers for Argentina strains (561-639)
