# Supplementary material for: Global population structure and adaptive evolution of aflatoxin‐producing fungi
Source: Ecol Evol. 2017 Sep 30;7(21):9179–91. doi: 10.1002/ece3.3464 (PMC5677503; doi:10.1002/ece3.3464)
Supplement: Supplementary file 18 [file ECE3-7-9179-s018.docx]

Table S6. GenBank accession numbers for *A. tamarii* sequences used in this study

| **IC Strain** | ***W/X*** | ***MAT*** | ***amdS*** | ***mfs*** | ***trpC*** |
| --- | --- | --- | --- | --- | --- |
| 1309 | HQ002424 |  | HQ000221 | KX853162 | HQ001135 |
| 1310 | HQ002425 | HQ001818 | HQ000222 | KX853163 | HQ001136 |
| 1311 | HQ002426 | HQ001819 | HQ000223 |  | HQ001137 |
| 1325 | HQ002427 | HQ002138 | HQ000226 |  | HQ001140 |
| 1345 | HQ002428 |  |  |  |  |
| 1353 | HQ002429 | HQ001824 | HQ000228 |  | HQ001145 |
| 1355 | HQ002430 | HQ001825 |  |  | HQ001146 |
| 1525 | HQ002431 | HQ001826 |  |  |  |
| 1526 | HQ002432 | HQ002143 |  |  |  |
| 1529 | HQ002433 | HQ001828 |  |  |  |
| 1530 | HQ002434 | HQ001829 | HQ000232 |  |  |
| 1531 | HQ002435 | HQ002145 |  |  |  |
| 1532 | HQ002436 | HQ001830 | HQ000233 |  |  |
| 1533 | HQ002437 | HQ001831 | HQ000234 |  | HQ001149 |
| 1534 | HQ002438 | HQ001832 | HQ000235 |  | HQ001150 |
| 1535 | HQ002439 | HQ001833 | HQ000236 |  | HQ001151 |
| 1537 | HQ002440 | HQ001835 | HQ000237 |  | HQ001152 |
| 1538 | HQ002441 | HQ001836 | HQ000238 |  | HQ001153 |
| 1539 | HQ002442 | HQ001837 | HQ000239 | HQ001117 | HQ001154 |
| 1540 | HQ002443 | HQ001838 | HQ000240 |  | HQ001155 |
| 1541 | HQ002444 | HQ002146 | HQ000241 |  | HQ001156 |
| 1542 | HQ002445 | HQ002147 | HQ000242 |  | HQ001157 |
| 1543 | HQ002446 | HQ002148 | HQ000243 |  | HQ001158 |
| 1545 | HQ002447 | HQ001839 | HQ000244 |  | HQ001159 |
| 1548 | HQ002448 | HQ002151 | HQ000245 |  | HQ001160 |
| 1549 | HQ002449 | HQ002152 | HQ000246 |  | HQ001161 |
| 1550 | HQ002450 | HQ002153 | HQ000247 |  | HQ001162 |
| 1552 | HQ002451 | HQ002155 | HQ000248 |  | HQ001163 |
| 1553 | HQ002452 | HQ002156 |  |  |  |
| 1554 | HQ002453 | HQ002157 |  |  |  |
| 1556 | HQ002454 | HQ002159 |  |  |  |
| 1557 | HQ002455 | HQ002160 |  |  | HQ001164 |
| 164 | HQ002456 | HQ001841 | HQ000249 |  | HQ001165 |

IC numbers for U.S.A. strains (1525-1557; 164)

IC numbers for India strains (1309-1355)
