# Supplementary material for: Global population structure and adaptive evolution of aflatoxin‐producing fungi
Source: Ecol Evol. 2017 Sep 30;7(21):9179–91. doi: 10.1002/ece3.3464 (PMC5677503; doi:10.1002/ece3.3464)
Supplement: Supplementary file 19 [file ECE3-7-9179-s019.docx]

Table S7. GenBank accession numbers for *A. oryzae* sequences used in this study

| **IC Strain** | ***W/X*** | ***M/N*** | ***MAT*** | ***amdS*** | ***mfs*** | ***trpC*** |
| --- | --- | --- | --- | --- | --- | --- |
| 1180 | HQ002592 | HQ002704 | HQ001850 | HQ000305 | HQ001038 | HQ001402 |
| 1181 | HQ002593 | HQ002707 | HQ001851 | HQ000304 | HQ001039 | HQ001403 |
| 1182 | HQ002595 |  |  |  |  |  |
| 1183 | HQ002594 | HQ002693 | HQ002048 | HQ000309 | HQ001040 | HQ001404 |
| 1184 | HQ002590 | HQ002705 | HQ001852 | HQ000308 | HQ001041 | HQ001405 |
| 1185 | HQ002589 |  | HQ001853 |  |  |  |
| 1186 | HQ002588 | HQ002719 | HQ001854 | HQ000310 | HQ001042 | HQ001406 |
| 1187 | HQ002587 | HQ002702 | HQ001855 | HQ000302 | HQ001043 | HQ001407 |
| 1188 | HQ002586 | HQ002720 | HQ001856 | HQ000307 | HQ001044 | HQ001408 |
| 1189 | HQ002585 | HQ002717 | HQ001857 | HQ000306 | HQ001045 | HQ001409 |
| 1190 | HQ002584 | HQ002716 | HQ001858 | HQ000301 | HQ001046 | HQ001410 |
| 1191 | HQ002602 |  | HQ001859 |  |  |  |
| 1192 |  |  | HQ001860 |  |  |  |
| 1193 | HQ002603 | HQ002689 | HQ002049 | HQ000300 | HQ001047 | HQ001411 |
| 1194 | HQ002604 | HQ002701 | HQ002050 | HQ000299 | HQ001048 | HQ001412 |
| 1195 | HQ002606 | HQ002714 | HQ001861 | HQ000298 | HQ001049 | HQ001413 |
| 1196 | HQ002605 | HQ002715 | HQ001862 | HQ000297 | HQ001050 | HQ001414 |
| 1197 | HQ002601 |  | HQ002051 | HQ000296 | HQ001051 | HQ001415 |
| 1199 | HQ002599 |  |  |  |  |  |
| 1200 | HQ002598 | HQ002718 | HQ001863 | HQ000295 | HQ001052 | HQ001416 |
| 1202 | HQ002597 | HQ002698 | HQ002052 | HQ000294 | HQ001053 | HQ001417 |
| 1203 | HQ002596 |  |  |  |  |  |
| 1204 | HQ002567 |  | HQ001864 |  |  |  |
| 1205 | HQ002568 | HQ002713 | HQ001865 | HQ000319 | HQ001054 | HQ001418 |
| 1206 | HQ002569 | HQ002712 | HQ001866 | HQ000293 | HQ001055 | HQ001419 |
| 1207 | HQ002570 | HQ002699 | HQ001867 | HQ000321 | HQ001056 | HQ001420 |
| 1208 | HQ002572 |  | HQ002053 | HQ000322 | HQ001057 | HQ001421 |
| 1209 | HQ002571 | HQ002711 | HQ001868 | HQ000323 | HQ001058 | HQ001422 |
| 1210 | HQ002566 | HQ002697 | HQ001869 | HQ000324 | HQ001059 | HQ001423 |
| 1211 | HQ002565 |  |  |  |  |  |
| 1212 | HQ002564 |  |  |  |  |  |
| 1214 | HQ002563 |  |  |  |  |  |
| 1216 | HQ002562 |  |  |  |  |  |
| 1217 | HQ002561 | HQ002696 | HQ001870 | HQ000325 | HQ001060 | HQ001424 |
| 1218 | HQ002560 | HQ002706 | HQ001871 | HQ000320 | HQ001061 | HQ001425 |
| 1219 | HQ002579 |  |  |  |  |  |
| 1221 | HQ002580 | HQ002691 | HQ001872 | HQ000318 | HQ001062 | HQ001426 |
| 1222 | HQ002581 | HQ002692 | HQ001873 | HQ000317 | HQ001063 | HQ001427 |
| 1223 | HQ002583 | HQ002710 | HQ001874 | HQ000316 | HQ001064 | HQ001428 |
| 1224 | HQ002582 | HQ002695 | HQ001875 | HQ000315 | HQ001065 | HQ001429 |
| 1225 | HQ002578 |  | HQ001876 |  |  |  |
| 1226 | HQ002577 | HQ002700 | HQ001877 | HQ000314 | HQ001066 | HQ001430 |
| 900 | HQ002591 | HQ002703 | HQ002054 | HQ000303 | HQ001067 | HQ001431 |
| 901 | HQ002576 | HQ002709 | HQ001878 | HQ000313 | HQ001068 | HQ001432 |
| 902 | HQ002575 | HQ002694 | HQ001879 | HQ000312 | HQ001069 | HQ001433 |
| 903 | HQ002574 | HQ002708 | HQ001880 | HQ000311 | HQ001070 | HQ001434 |

IC numbers for Japan strains (1180-1226; 900-903)
