# Supplementary material for: Global population structure and adaptive evolution of aflatoxin‐producing fungi
Source: Ecol Evol. 2017 Sep 30;7(21):9179–91. doi: 10.1002/ece3.3464 (PMC5677503; doi:10.1002/ece3.3464)
Supplement: Supplementary file 20 [file ECE3-7-9179-s020.docx]

Table S8. GenBank accession numbers for *A. nomius* sequences used in this study

| **IC Strain** | ***W/X*** | ***M/N*** | ***MAT*** | ***amdS*** | ***trpC*** |
| --- | --- | --- | --- | --- | --- |
| 157 | HQ002618 | HQ002851 | HQ001936 | HQ000290 | HQ001324 |
| 1493 |  |  | HQ002185 |  |  |
| 1494 |  |  | HQ001922 | HQ000287 |  |
| 1495 |  |  | HQ002186 |  |  |
| 1496 | HQ002631 |  | HQ002187 |  |  |
| 1497 | HQ002630 |  | HQ002188 |  |  |
| 1498 | HQ002626 |  | HQ002189 | HQ000288 | HQ001316 |
| 1499 |  |  | HQ002190 |  |  |
| 1500 |  |  | HQ002191 |  |  |
| 1501 |  |  | HQ002192 |  |  |
| 1502 | HQ002625 |  | HQ002193 |  |  |
| 1503 |  |  | HQ002194 |  |  |
| 1504 | HQ002622 |  | HQ002195 |  |  |
| 1505 |  |  | HQ002196 |  |  |
| 1506 | HQ002623 |  | HQ002197 |  |  |
| 1507 | HQ002624 |  | HQ002198 |  |  |
| 1508 | HQ002629 |  | HQ002199 |  | HQ001317 |
| 1509 |  |  | HQ002200 |  |  |
| 1510 | HQ002621 |  | HQ001923 |  | HQ001318 |
| 1511 | HQ002610 |  | HQ001924 |  | HQ001319 |
| 1512_M1^a^ | HQ002611 |  | HQ001925 |  |  |
| 1512_M2 ^a^ |  |  | HQ002201 |  |  |
| 1513 |  |  | HQ001926 |  |  |
| 1514_M1 ^a^ |  |  | HQ001927 |  |  |
| 1514_M2 ^a^ |  |  | HQ002202 |  |  |
| 1516_M1 ^a^ |  |  | HQ001928 | HQ000284 | HQ001320 |
| 1516_M2 ^a^ |  |  | HQ002203 |  |  |
| 1517 | HQ002612 |  | HQ001929 |  |  |
| 1518 | HQ002609 |  | HQ002204 | HQ000289 | HQ001321 |
| 1519_M1 ^a^ |  |  | HQ001930 |  |  |
| 1520_M1 ^a^ | HQ002608 |  | HQ001931 |  |  |
| 1520_M2 ^a^ |  |  | HQ002206 |  |  |
| 1521_M1 ^a^ | HQ002607 |  | HQ001932 |  |  |
| 1521_M2 ^a^ |  |  | HQ002207 |  |  |
| 1522_M1 ^a^ | HQ002627 |  | HQ001933 |  |  |
| 1522_M2 ^a^ |  |  | HQ002208 |  |  |
| 1523 | HQ002628 |  | HQ001934 | HQ000285 | HQ001322 |
| 1524 | HQ002620 |  | HQ001935 | HQ000286 | HQ001323 |

^a^ Strains that amplified both mating-type idiomorphs

IC numbers for U.S.A. strains (1493-1524; 157)
