# Supplementary material for: Global population structure and adaptive evolution of aflatoxin‐producing fungi
Source: Ecol Evol. 2017 Sep 30;7(21):9179–91. doi: 10.1002/ece3.3464 (PMC5677503; doi:10.1002/ece3.3464)
Supplement: Supplementary file 21 [file ECE3-7-9179-s021.docx]

Table S9. GenBank accession numbers for *A. alliaceus* and *A. sojae* sequences used in this study

| **IC Strain** | ***W/X*** | ***M/N*** | ***MAT*** | ***amdS*** | ***mfs*** | ***trpC*** |
| --- | --- | --- | --- | --- | --- | --- |
| 886 |  | HQ002852 |  |  |  |  |
| 887 |  |  | HQ002209 |  |  |  |
| 888 | HQ002619 |  | HQ001937 |  |  |  |
| 889 | HQ002617 | HQ002853 | HQ002210 |  |  |  |
| 890 | HQ002616 |  | HQ002211 |  |  |  |
| 891 | HQ002615 | HQ002854 | HQ002212 | HQ000292 |  | HQ001325 |
| 892_M1^a^ | HQ002614 |  | HQ001938 |  | HQ001119 |  |
| 892_M2 ^a^ |  |  | HQ002213 |  |  |  |
| 893 |  |  | HQ002214 |  |  |  |
| 894 |  | HQ002855 | HQ002215 |  |  |  |
| 1215 | HQ002613 | HQ002850 | HQ001921 | HQ000291 | HQ001118 | HQ001315 |

^a^ Strains that amplified both mating-type idiomorphs

IC numbers for Australian *A. allicaeus* strains (886-894)

IC number for *A. sojae* Type strain (1215)
