# Supplementary material for: Global population structure and adaptive evolution of aflatoxin‐producing fungi
Source: Ecol Evol. 2017 Sep 30;7(21):9179–91. doi: 10.1002/ece3.3464 (PMC5677503; doi:10.1002/ece3.3464)
Supplement: Supplementary file 22 [file ECE3-7-9179-s022.doc]

Table S10. Haplotype identities for *amdS* heuristic phylogeny in Figure S1

| Haplotype | Isolate Identities |
| --- | --- |
| H1 | IC157, IC1518 |
| H2 | IC1000, IC1001, IC1002, IC1360, IC999 |
| H3 | IC1027, IC1046, IC1051, IC297, IC659, IC708 |
| H4 | IC1494, IC1498 |
| H5 | IC317, IC318, IC319, IC320, IC321, IC322, IC323, IC324, IC325, IC326, IC58, IC59, IC60, IC65, IC66, IC70, IC71, IC72, IC744, IC74, IC76, IC800, IC801, IC804, IC805, IC807, IC809, IC828, IC832, IC837, IC839, IC840, IC848, IC854, IC863, IC908, IC909, IC910, IC912, IC913, IC915, IC916, IC917, IC918, IC919, IC923, IC924, IC926, IC927 |
| H6 | IC517, IC518 |
| H7 | IC816, IC825 |
| H8 | IC494, IC526, IC860, IC876 |
| H9 | IC723, IC725, IC727, IC728, IC729, IC732, IC736, IC737, IC741, IC748, IC749, IC762, IC770, IC779, IC780, IC792, IC796, IC797 |
| H10 | IC1112, IC1117, IC1140, IC1144, IC1146, IC1148, IC1150 |
| H11 | IC1145, IC1147, IC1151 |
| H12 | IC1028, IC1030, IC1031, IC1032, IC1034, IC1036, IC1037, IC1038, IC1039, IC1041, IC1042, IC1043, IC1044, IC1045, IC1047, IC1048, IC1049, IC1050, IC1052, IC1053, IC1054, IC1055, IC1056, IC1058, IC1059, IC1061, IC1063, IC1065, IC1067, IC1068, IC1069, IC1070, IC1071, IC1072, IC1073, IC1074, IC1075, IC1077, IC1079, IC1080, IC1081, IC1082, IC1083, IC1084, IC1085, IC1089, IC1090, IC1091, IC1092, IC1093, IC1094, IC1096, IC1098, IC1099, IC1100, IC1101, IC1102, IC1103, IC1104, IC1106, IC1118, IC1134, IC1149, IC1152, IC1154, IC1155, IC1156, IC1157, IC1160, IC1161, IC1163, IC1164, IC1165, IC1167, IC1168, IC1169, IC1171, IC1174, IC1175, IC1176, IC1177, IC1178, IC1180, IC1181, IC1183, IC1184, IC1186, IC1187, IC1188, IC1189, IC1190, IC1193, IC1194, IC1195, IC1196, IC1197, IC1200, IC1202, IC1205, IC1206, IC1207, IC1208, IC1209, IC1210, IC1217, IC1218, IC1221, IC1222, IC1223, IC1224, IC1226, IC1227, IC1228, IC1229, IC1230, IC1233, IC1237, IC1241, IC1249, IC1252, IC1253, IC1260, IC1262, IC1264, IC1265, IC1266, IC1269, IC1270, IC1271, IC1272, IC1275, IC1277, IC1281, IC1291, IC1297, IC1303, IC1305, IC1306, IC1307, IC1311, IC204, IC219, IC220, IC221, IC222, IC223, IC227, IC228, IC229, IC232, IC233, IC234, IC237, IC238, IC239, IC240, IC241, IC242, IC243, IC245, IC248, IC249, IC250, IC251, IC263, IC264, IC265, IC272, IC273, IC274, IC275, IC276, IC277, IC279, IC280, IC283, IC284, IC285, IC286, IC287, IC289, IC290, IC292, IC293, IC294, IC296, IC299, IC300, IC301, IC302, IC304, IC307, IC311, IC314, IC315, IC397, IC398, IC399, IC400, IC401, IC404, IC405, IC406, IC407, IC408, IC409, IC410, IC412, IC414, IC415, IC417, IC418, IC419, IC420, IC421, IC422, IC423, IC424, IC427, IC429, IC430, IC431, IC434, IC440, IC442, IC443, IC444, IC445, IC452, IC453, IC455, IC456, IC457, IC458, IC460, IC461, IC468, IC469, IC470, IC471, IC568, IC643, IC646, IC650, IC651, IC652, IC655, IC656, IC657, IC658, IC660, IC661, IC662, IC663, IC664, IC672, IC673, IC674, IC675, IC676, IC677, IC678, IC679, IC680, IC682, IC683, IC684, IC685, IC686, IC688, IC696, IC697, IC698, IC701, IC702, IC703, IC711, IC719, IC891, IC901, IC902, IC903, IC904 |
| H13 | IC1251 |
| H14 | IC1257, IC1282, IC1290, IC1296 |
| H15 | IC1319, IC1321, IC1331 |
| H16 | IC617 |
| H17 | IC1141, IC1153, IC1162, IC258, IC259, IC260, IC261, IC262, IC308, IC402, IC413, IC425, IC428, IC432, IC433, IC435, IC437, IC439, IC447, IC448, IC449, IC459, IC463, IC464, IC465, IC466, IC467, IC476, IC479 |
| H18 | IC1274, IC1293 |
| H19 | IC310, IC313, IC396, IC403, IC438, IC441, IC451, IC454, IC474, IC580, IC671, IC900 |
| H20 | IC1035, IC1057, IC1066, IC1179, IC1239, IC1254, IC1258, IC1279, IC1295, IC1304, IC203, IC267, IC268, IC269, IC270, IC271, IC278, IC281, IC295, IC298, IC303, IC305, IC309, IC312, IC411, IC416, IC426, IC436, IC446, IC450, IC462, IC472, IC475, IC695, IC899 |
| H21 | IC1064 |
| H22 | IC1280 |
| H23 | IC1033 |
| H24 | IC217, IC218, IC225, IC226, IC244, IC252, IC253, IC254, IC255, IC256, IC257, IC316 |
| H25 | IC1060, IC288, IC640, IC648, IC666, IC667, IC670, IC704, IC709, IC712 |
| H26 | IC1029, IC1040, IC1062, IC1076, IC1078, IC1086, IC1087, IC1088, IC1095, IC1097, IC1105, IC1245, IC1250, IC1255, IC1268, IC1276, IC1309, IC1310, IC282, IC291, IC306 |
| H27 | IC1580, IC1581 |
| H28 | IC806 |
| H29 | IC981 |
| H30 | IC1559, IC1560, IC1561, IC1562, IC1563, IC1564, IC1565, IC1566, IC1569, IC1571, IC1572, IC1573, IC1574, IC1575, IC162, IC567, IC591, IC598, IC599, IC601, IC611, IC613, IC624, IC626, IC630, IC633, IC634, IC636, IC639 |
| H31 | IC1577, IC1584, IC565 |
| H32 | IC570 |
| H33 | IC618 |
| H34 | IC577 |
| H35 | IC1006, IC1008, IC1009, IC1013, IC1014, IC1364, IC947, IC949, IC951, IC958, IC974, IC995 |
| H36 | IC164 |
| H37 | IC1353 |
| H38 | IC1003, IC1007, IC1010, IC1011, IC1015, IC1016, IC1017, IC1018, IC1020, IC1021, IC1022, IC1023, IC1024, IC953, IC955, IC960, IC961, IC966, IC967, IC968, IC971, IC972, IC976, IC978, IC982, IC992, IC993, IC994, IC996, IC997 |
| H39 | IC962 |
| H40 | IC1550, IC1552, IC984, IC986, IC987, IC988 |
| H41 | IC1005, IC1012, IC1325, IC1357, IC1530, IC1532, IC1533, IC1534, IC1535, IC1537, IC1538, IC1539, IC1540, IC1541, IC1542, IC1543, IC1545, IC1548, IC1549, IC948, IC950, IC956, IC963 |
|  |  |
|  |  |
| H42 | IC100, IC101, IC102, IC105, IC106, IC107, IC108, IC109, IC10, IC110, IC111, IC112, IC115, IC118, IC119, IC11, IC123, IC125, IC126, IC128, IC129, IC12, IC130, IC131, IC133, IC134, IC135, IC136, IC137, IC138, IC139, IC13, IC140, IC141, IC142, IC143, IC144, IC14, IC15, IC17, IC1, IC2, IC327, IC329, IC32, IC330, IC331, IC33, IC34, IC35, IC36, IC37, IC38, IC39, IC40, IC42, IC484, IC486, IC487, IC489, IC491, IC496, IC497, IC502, IC504, IC505, IC506, IC507, IC508, IC509, IC510, IC512, IC513, IC514, IC516, IC520, IC521, IC522, IC529, IC52, IC530, IC532, IC533, IC53, IC540, IC541, IC542, IC543, IC545, IC549, IC54, IC553, IC554, IC555, IC556, IC55, IC56, IC5, IC642, IC68, IC69, IC73, IC75, IC77, IC7, IC81, IC824, IC83, IC844, IC84, IC851, IC868, IC86, IC88, IC8, IC906, IC907, IC911, IC920, IC95, IC96, IC97, IC98, IC99 |
| H43 | IC1113, IC1119, IC1120, IC1121, IC1133, IC1135, IC1142 |
| H44 | IC1107, IC328, IC490, IC534 |
| H45 | IC1215 |
| H46 | IC18, IC19, IC21, IC22, IC23, IC24, IC25, IC26, IC27, IC29, IC44, IC46, IC47, IC480, IC485, IC48, IC495, IC499, IC49, IC500, IC50, IC511, IC519, IC51, IC523, IC524, IC525, IC528, IC531, IC535, IC536, IC537, IC538, IC539, IC544, IC546, IC547, IC548, IC551, IC552, IC61, IC62, IC63, IC64, IC67, IC808, IC814, IC822, IC835, IC853, IC864, IC867, IC872, IC875, IC905, IC921, IC922, IC925 |
| H47 | IC811, IC813, IC836 |
| H48 | IC731 |
| H49 | IC477, IC742, IC790, IC791, IC793 |
| H50 | IC733, IC735, IC758, IC778, IC799 |
| H51 | IC720 |
| H52 | IC478 |
| H53 | IC743, IC751, IC753, IC755, IC760, IC768, IC777, IC785, IC786, IC787, IC788, IC798 |
| H54 | IC1523, IC1524 |
| H55 | IC1516 |

*A. alliaceus* (886-894)

*A. caelatus* (162; 560-639; 1559-1589)

*A. flavus* L (203-316; 396-475; 640-719; 899; 1179; 1027-1106; 1227; 1229-1308)

*A. flavus* S (476-479; 720-799; 1110-1178; 1228)

*A. nomius* (157; 1493-1524)

*A. oryzae* (900-904; 1180-1214; 1216-1226)

*A. parasiticus* (1-144; 317-331; 480-559; 800-876; 905-927; 1107)

*A. sojae* (1215)

*A. tamarii* (164; 947-1026; 1309-1364; 1525-1558)

* Underlined numbers indicate evidence of trans-speciation among the majority of isolates sharing a haplotype.
