# Supplementary material for: Global population structure and adaptive evolution of aflatoxin‐producing fungi
Source: Ecol Evol. 2017 Sep 30;7(21):9179–91. doi: 10.1002/ece3.3464 (PMC5677503; doi:10.1002/ece3.3464)
Supplement: Supplementary file 23 [file ECE3-7-9179-s023.doc]

Table S11. Haplotype identities for *trpC* heuristic phylogeny in Figure S1

| Haplotype | Isolate Identities |
| --- | --- |
| H1 | IC157 |
| H2 | IC1027, IC1028, IC1037, IC1039, IC1041, IC1043, IC1045, IC1046, IC1047, IC1049, IC1051, IC1053, IC1055, IC1056, IC1059, IC1062, IC1065, IC1067, IC1068, IC1070, IC1071, IC1072, IC1073, IC1080, IC1081, IC1088, IC1089, IC1092, IC1095, IC1100, IC1102, IC1103, IC1104, IC1105, IC1180, IC1184, IC1187, IC1194, IC1197, IC1207, IC1208, IC1226, IC1230, IC1233, IC1237, IC1249, IC1251, IC1254, IC1257, IC1260, IC1268, IC1282, IC1290, IC1296, IC243, IC244, IC245, IC248, IC249, IC250, IC251, IC278, IC280, IC281, IC285, IC288, IC289, IC292, IC293, IC303, IC304, IC306, IC307, IC396, IC399, IC400, IC403, IC406, IC407, IC412, IC420, IC429, IC430, IC438, IC441, IC443, IC444, IC451, IC454, IC455, IC474, IC640, IC642, IC646, IC650, IC651, IC652, IC655, IC660, IC664, IC666, IC667, IC671, IC674, IC677, IC679, IC683, IC684, IC695, IC697, IC701, IC703, IC711, IC899, IC902 |
| H3 | IC1258 |
| H4 | IC1305, IC676 |
| H5 | IC1295 |
| H6 | IC678 |
| H7 | IC1306 |
| H8 | IC1029, IC1052, IC1054, IC1061, IC1087, IC1227, IC1245, IC1265, IC1279, IC1281, IC227, IC228, IC229, IC232, IC233, IC274, IC275, IC279, IC283, IC284, IC286, IC287, IC291, IC295, IC298, IC312, IC405, IC410, IC421, IC648, IC662, IC673, IC680, IC682, IC686, IC696, IC709, IC712 |
| H9 | IC1030, IC1032, IC1034, IC1036, IC1040, IC1042, IC1044, IC1048, IC1050, IC1058, IC1060, IC1063, IC1069, IC1074, IC1075, IC1076, IC1077, IC1078, IC1079, IC1082, IC1083, IC1084, IC1085, IC1086, IC1091, IC1093, IC1094, IC1096, IC1097, IC1099, IC1101, IC1106, IC1153, IC1154, IC1156, IC1157, IC1160, IC1161, IC1162, IC1163, IC1164, IC1165, IC1167, IC1168, IC1169, IC1171, IC1174, IC1175, IC1176, IC1177, IC1178, IC1179, IC1229, IC1239, IC1241, IC1250, IC1262, IC1266, IC1269, IC1271, IC1272, IC1277, IC1280, IC1303, IC1304, IC1307, IC204, IC217, IC218, IC219, IC220, IC221, IC222, IC223, IC225, IC226, IC234, IC237, IC238, IC239, IC240, IC241, IC242, IC252, IC253, IC254, IC255, IC256, IC257, IC258, IC259, IC260, IC261, IC262, IC267, IC268, IC269, IC277, IC282, IC290, IC294, IC296, IC297, IC300, IC301, IC308, IC309, IC310, IC311, IC315, IC316, IC397, IC398, IC401, IC402, IC404, IC408, IC409, IC413, IC414, IC415, IC417, IC418, IC419, IC422, IC423, IC424, IC425, IC427, IC428, IC431, IC432, IC433, IC434, IC435, IC436, IC437, IC439, IC442, IC445, IC447, IC448, IC449, IC452, IC453, IC456, IC457, IC458, IC459, IC460, IC461, IC463, IC464, IC465, IC467, IC469, IC470, IC471, IC476, IC479, IC617, IC643, IC656, IC658, IC672, IC685, IC688, IC702, IC704, IC719 |
| H10 | IC1145, IC1147, IC1151 |
| H11 | IC1112 |
| H12 | IC1113, IC1118, IC1119, IC1120, IC1121, IC1133, IC1134, IC1135, IC1141, IC1142 |
| H13 | IC1117, IC1140, IC1144, IC1146, IC1148, IC1149, IC1150 |
| H14 | IC1228 |
| H15 | IC478, IC720, IC723, IC725, IC727, IC728, IC729, IC731, IC732, IC733, IC735, IC736, IC737, IC741, IC742, IC743, IC744, IC748, IC749, IC751, IC753, IC755, IC758, IC760, IC762, IC768, IC770, IC777, IC778, IC779, IC780, IC785, IC786, IC787, IC788, IC790, IC791, IC792, IC793, IC796, IC797, IC798, IC799 |
| H16 | IC1155, IC1183, IC1188, IC1190, IC1193, IC1195, IC1200, IC1202, IC1205, IC1206, IC1209, IC1221, IC1222, IC1223, IC313, IC314, IC900, IC903 |
| H17 | IC1309, IC1311 |
| H18 | IC1264, IC1275, IC440, IC468, IC675 |
| H19 | IC1255 |
| H20 | IC1031, IC1033, IC1035, IC1038, IC1057, IC1064, IC1066, IC1090, IC1098, IC1152, IC1181, IC1186, IC1189, IC1196, IC1210, IC1217, IC1218, IC1224, IC1253, IC1270, IC1274, IC1276, IC1291, IC1293, IC1297, IC203, IC263, IC264, IC265, IC270, IC271, IC272, IC273, IC276, IC299, IC302, IC305, IC411, IC416, IC426, IC446, IC450, IC462, IC466, IC472, IC475, IC477, IC657, IC659, IC661, IC663, IC670, IC698, IC708, IC901, IC904 |
| H21 | IC1252 |
| H22 | IC580 |
| H23 | IC891 |
| H24 | IC633, IC639 |
| H25 | IC839 |
| H26 | IC863 |
| H27 | IC867 |
| H28 | IC569 |
| H29 | IC1001, IC1005, IC1012, IC1310, IC1319, IC1321, IC1325, IC1327, IC1331, IC1343, IC1353, IC1355, IC1357, IC1360, IC1533, IC1534, IC1535, IC1537, IC1538, IC1539, IC1540, IC1541, IC1542, IC1543, IC1545, IC1548, IC1549, IC1550, IC1552, IC948, IC950, IC956, IC963, IC984 |
| H30 | IC100, IC101, IC102, IC105, IC106, IC107, IC108, IC109, IC10, IC1107, IC110, IC111, IC112, IC115, IC118, IC119, IC11, IC123, IC125, IC126, IC128, IC129, IC12, IC130, IC131, IC133, IC134, IC135, IC136, IC137, IC138, IC139, IC13, IC140, IC141, IC142, IC143, IC144, IC14, IC15, IC17, IC18, IC19, IC1, IC21, IC22, IC23, IC24, IC25, IC26, IC27, IC29, IC2, IC317, IC318, IC319, IC320, IC321, IC322, IC323, IC324, IC325, IC326, IC327, IC328, IC329, IC32, IC330, IC331, IC33, IC34, IC35, IC36, IC37, IC38, IC39, IC40, IC42, IC44, IC46, IC47, IC480, IC484, IC485, IC486, IC487, IC489, IC48, IC490, IC494, IC495, IC496, IC497, IC499, IC49, IC500, IC502, IC504, IC505, IC506, IC507, IC508, IC509, IC50, IC510, IC511, IC512, IC513, IC514, IC516, IC517, IC518, IC519, IC51, IC520, IC521, IC522, IC523, IC524, IC525, IC526, IC528, IC529, IC52, IC530, IC531, IC532, IC533, IC534, IC535, IC536, IC537, IC538, IC539, IC53, IC540, IC541, IC542, IC543, IC544, IC545, IC546, IC547, IC548, IC549, IC54, IC551, IC552, IC553, IC554, IC555, IC556, IC55, IC561, IC564, IC565, IC566, IC567, IC568, IC56, IC570, IC571, IC572, IC573, IC574, IC577, IC578, IC588, IC58, IC590, IC598, IC599, IC59, IC5, IC603, IC604, IC60, IC615, IC61, IC62, IC63, IC64, IC65, IC66, IC67, IC68, IC69, IC70, IC71, IC72, IC73, IC74, IC75, IC76, IC77, IC78, IC7, IC800, IC801, IC804, IC805, IC806, IC807, |
|  |  |
| H30 | IC808, IC809, IC811, IC813, IC814, IC816, IC81, IC822, IC824, IC825, IC828, IC832, IC835, IC836, IC837, IC83, IC840, IC844, IC848, IC84, IC851, IC853, IC854, IC860, IC864, IC868, IC86, IC872, IC875, IC876, IC88, IC8, IC905, IC906, IC907, IC908, IC909, IC910, IC911, IC912, IC913, IC915, IC916, IC917, IC918, IC919, IC920, IC921, IC922, IC923, IC924, IC925, IC926, IC927, IC95, IC96, IC97, IC98, IC99 |
| H31 | IC1559, IC1560, IC1562, IC1564, IC1565, IC1566, IC1568, IC1569, IC1570, IC1572, IC1573, IC1574, IC1576, IC1577, IC1584, IC162, IC582, IC591, IC611, IC613, IC618, IC624, IC626, IC630, IC634, IC636 |
| H32 | IC1580, IC1581 |
| H33 | IC616 |
| H34 | IC600 |
| H35 | IC1215 |
| H36 | IC1006, IC1007, IC1008, IC1009, IC1011, IC1013, IC1015, IC1017, IC1020, IC1022, IC1023, IC1024, IC1332, IC1557, IC164, IC947, IC949, IC953, IC955, IC958, IC960, IC961, IC966, IC967, IC972, IC974, IC982, IC992, IC994, IC996 |
| H37 | IC1498, IC1511, IC1516 |
| H38 | IC1508, IC1510 |
| H39 | IC1518, IC1523, IC1524 |

*A. alliaceus* (886-894)

*A. caelatus* (162; 560-639; 1559-1589)

*A. flavus* L (203-316; 396-475; 640-719; 899; 1179; 1027-1106; 1227; 1229-1308)

*A. flavus* S (476-479; 720-799; 1110-1178; 1228)

*A. nomius* (157; 1493-1524)

*A. oryzae* (900-904; 1180-1214; 1216-1226)

*A. parasiticus* (1-144; 317-331; 480-559; 800-876; 905-927; 1107)

*A. sojae* (1215)

*A. tamarii* (164; 947-1026; 1309-1364; 1525-1558)

* Underlined numbers indicate evidence of trans-speciation among the majority of isolates sharing a haplotype.
