# Supplementary material for: Global population structure and adaptive evolution of aflatoxin‐producing fungi
Source: Ecol Evol. 2017 Sep 30;7(21):9179–91. doi: 10.1002/ece3.3464 (PMC5677503; doi:10.1002/ece3.3464)
Supplement: Supplementary file 24 [file ECE3-7-9179-s024.doc]

Table S12. Haplotype identities for *MAT1-1* heuristic phylogeny in Figure S2

| Haplotype | Isolate Identities |
| --- | --- |
| H1 | IC157 |
| H2 | IC1512 |
| H3 | IC1520 |
| H4 | IC892 |
| H5 | IC1353 |
| H6 | IC1000, IC1004, IC1017, IC1020, IC1191, IC1192, IC1510, IC1511, IC1513, IC1519, IC1521, IC1522, IC1524, IC1525, IC1527, IC1529, IC1530, IC1532, IC1533, IC1534, IC1535, IC1536, IC1537, IC1538, IC1539, IC1540, IC1545, IC1546, IC164, IC960, IC967, IC971, IC974, IC980, IC981, IC993, IC999 |
| H7 | IC888 |
| H8 | IC1559, IC1560, IC1561, IC1562, IC1563, IC1564, IC1565, IC1566, IC1567, IC1568, IC1569, IC1570, IC1571, IC1572, IC1575, IC1576, IC1577, IC1578, IC1579, IC1583, IC1585, IC1586, IC1587, IC1588, IC1589, IC565, IC569, IC577, IC582, IC598, IC599, IC600, IC601, IC602, IC603, IC605, IC606, IC610, IC614, IC616, IC620, IC622, IC626, IC627, IC628, IC629, IC630, IC631, IC632, IC634, IC639 |
| H9 | IC619 |
| H10 | IC100, IC101, IC102, IC103, IC104, IC105, IC106, IC107, IC108, IC109, IC10, IC110, IC111, IC112, IC113, IC114, IC115, IC116, IC117, IC118, IC11, IC120, IC121, IC123, IC124, IC125, IC127, IC128, IC129, IC12, IC130, IC131, IC132, IC1332, IC133, IC1343, IC134, IC135, IC136, IC137, IC138, IC139, IC13, IC140, IC141, IC142, IC143, IC144, IC14, IC15, IC16, IC17, IC1, IC2, IC30, IC32, IC33, IC34, IC36, IC37, IC39, IC40, IC41, IC42, IC484, IC486, IC487, IC489, IC491, IC496, IC497, IC4, IC502, IC504, IC505, IC506, IC507, IC508, IC509, IC510, IC512, IC514, IC516, IC520, IC521, IC522, IC528, IC529, IC52, IC530, IC532, IC533, IC53, IC540, IC541, IC542, IC543, IC544, IC545, IC549, IC54, IC553, IC554, IC555, IC55, IC568, IC56, IC5, IC67, IC68, IC71, IC72, IC74, IC75, IC77, IC78, IC79, IC7, IC80, IC81, IC83, IC84, IC86, IC88, IC89, IC8, IC90, IC91, IC92, IC93, IC94, IC95, IC96, IC97, IC98, IC99 |
| H11 | IC1027, IC1028, IC1030, IC1032, IC1035, IC1037, IC1041, IC1043, IC1044, IC1045, IC1048, IC1049, IC1050, IC1055, IC1056, IC1059, IC1060, IC1061, IC1062, IC1064, IC1066, IC1067, IC1068, IC1070, IC1071, IC1075, IC1076, IC1077, IC1078, IC1080, IC1081, IC1082, IC1084, IC1085, IC1086, IC1087, IC1088, IC1091, IC1092, IC1093, IC1094, IC1096, IC1098, IC1101, IC1103, IC1104, IC1105, IC1106, IC1153, IC1160, IC1161, IC1162, IC1164, IC1165, IC1167, IC1168, IC1169, IC1171, IC1174, IC1175, IC1176, IC1178, IC1180, IC1181, IC1184, IC1185, IC1186, IC1187, IC1188, IC1189, IC1190, IC1195, IC1196, IC1200, IC1204, IC1205, IC1206, IC1207, IC1209, IC1210, IC1217, IC1218, IC1221, IC1222, IC1223, IC1224, IC1226, IC1228, IC1233, IC1239, IC1241, IC1250, IC1251, IC1252, IC1253, IC1255, IC1257, IC1258, IC1262, IC1265, IC1266, IC1268, IC1269, IC126, IC1271, IC1272, IC1275, IC1276, IC1277, IC1280, IC1281, IC1282, IC1290, IC1291, IC1296, IC1297, IC1303, IC1304, IC1306, IC1307, IC1310, IC1311, IC1319, IC204, IC243, IC244, IC278, IC282, IC283, IC289, IC291, IC293, IC296, IC297, IC298, IC300, IC301, IC303, IC305, IC308, IC310, IC314, IC396, IC397, IC398, IC399, |
| H11 | IC400, IC401, IC402, IC403, IC404, IC406, IC407, IC408, IC410, IC411, IC412, IC413, IC414, IC416, IC418, IC419, IC420, IC421, IC422, IC423, IC425, IC426, IC428, IC429, IC430, IC431, IC432, IC433, IC434, IC435, IC436, IC437, IC438, IC439, IC441, IC442, IC443, IC444, IC445, IC446, IC447, IC448, IC449, IC450, IC451, IC452, IC453, IC454, IC455, IC456, IC458, IC460, IC461, IC462, IC463, IC464, IC465, IC466, IC467, IC469, IC472, IC474, IC475, IC580, IC617, IC648, IC650, IC652, IC65, IC662, IC670, IC671, IC675, IC682, IC684, IC685, IC688, IC695, IC702, IC704, IC708, IC709, IC712, IC899, IC901, IC902, IC903, IC904 |
| H12 | IC1112 |
| H13 | IC477, IC478, IC720, IC731, IC732, IC733, IC735, IC742, IC744, IC778, IC790, IC791, IC793, IC796, IC799 |
| H14 | IC1113, IC1118, IC1119, IC1120, IC1121, IC1133, IC1135, IC1141 |
| H15 | IC1063, IC217, IC218, IC219, IC220, IC225, IC226, IC311, IC315 |
| H16 | IC1355 |
| H17 | IC1107, IC119, IC1225, IC1327, IC18, IC19, IC21, IC22, IC23, IC24, IC25, IC26, IC27, IC28, IC29, IC35, IC44, IC45, IC46, IC47, IC480, IC485, IC48, IC490, IC495, IC499, IC49, IC500, IC50, IC511, IC513, IC517, IC518, IC519, IC51, IC523, IC524, IC525, IC531, IC534, IC535, IC536, IC537, IC538, IC539, IC546, IC547, IC548, IC551, IC552, IC556, IC561, IC61, IC63, IC64, IC70, IC811, IC813, IC824, IC825, IC835, IC836, IC844, IC851, IC864, IC868, IC872, IC875, IC905, IC921, IC922, IC925 |
| H18 | IC73, IC906, IC907, IC920 |
| H19 | IC62 |
| H20 | IC1516 |
| H21 | IC1523 |
| H22 | IC1514 |
| H23 | IC1517 |
| H24 | IC1494 |

*A. alliaceus* (886-894)

*A. caelatus* (162; 560-639; 1559-1589)

*A. flavus* L (203-316; 396-475; 640-719; 899; 1179; 1027-1106; 1227; 1229-1308)

*A. flavus* S (476-479; 720-799; 1110-1178; 1228)

*A. nomius* (157; 1493-1524)

*A. oryzae* (900-904; 1180-1214; 1216-1226)

*A. parasiticus* (1-144; 317-331; 480-559; 800-876; 905-927; 1107)

*A. sojae* (1215)

*A. tamarii* (164; 947-1026; 1309-1364; 1525-1558)

* Underlined numbers indicate evidence of trans-speciation among the majority of isolates sharing a haplotype.
