# Supplementary material for: Global population structure and adaptive evolution of aflatoxin‐producing fungi
Source: Ecol Evol. 2017 Sep 30;7(21):9179–91. doi: 10.1002/ece3.3464 (PMC5677503; doi:10.1002/ece3.3464)
Supplement: Supplementary file 25 [file ECE3-7-9179-s025.doc]

Table S13. Haplotype identities for *MAT1-2* heuristic phylogeny in Figure S2

| Haplotype | Isolate Identities |
| --- | --- |
| H1 | IC1493, IC1005, IC1006, IC1007, IC1008, IC1009, IC1011, IC1012, IC1013, IC1015, IC1022, IC1023, IC1024, IC1025, IC1495, IC1496, IC1497, IC1498, IC1505, IC1512, IC1520, IC1531, IC1541, IC1542, IC1543, IC1544, IC1547, IC1548, IC1549, IC1550, IC1551, IC1552, IC1553, IC1554, IC1555, IC1556, IC1557, IC1558, IC947, IC948, IC949, IC950, IC953, IC955, IC956, IC957, IC958, IC959, IC962, IC963, IC964, IC966, IC968, IC969, IC970, IC972, IC975, IC977, IC979, IC982, IC985, IC989, IC990, IC991, IC995, IC996, IC998 |
| H2 | IC1029, IC1031, IC1033, IC1034, IC1036, IC1038, IC1040, IC1042, IC1046, IC1047, IC1051, IC1052, IC1054, IC1057, IC1058, IC1065, IC1069, IC1072, IC1073, IC1074, IC1079, IC1083, IC1089, IC1090, IC1095, IC1097, IC1099, IC1100, IC1102, IC1152, IC1154, IC1155, IC1156, IC1157, IC1163, IC1177, IC1179, IC1183, IC1193, IC1194, IC1197, IC1202, IC1208, IC1227, IC1229, IC1230, IC1237, IC1245, IC1249, IC1254, IC1260, IC1263, IC1264, IC1270, IC1274, IC1279, IC1289, IC1293, IC1295, IC1300, IC1301, IC1305, IC1308, IC1321, IC1325, IC1331, IC1343, IC203, IC221, IC222, IC223, IC227, IC228, IC229, IC232, IC233, IC234, IC237, IC238, IC239, IC240, IC241, IC242, IC245, IC248, IC249, IC250, IC251, IC252, IC253, IC254, IC255, IC256, IC257, IC258, IC259, IC260, IC261, IC263, IC263, IC264, IC267, IC270, IC272, IC274, IC276, IC277, IC279, IC280, IC281, IC284, IC285, IC286, IC288, IC290, IC292, IC294, IC295, IC299, IC302, IC304, IC306, IC307, IC309, IC312, IC313, IC316, IC405, IC409, IC415, IC417, IC424, IC427, IC440, IC457, IC459, IC468, IC470, IC471, IC476, IC479, IC567, IC570, IC640, IC642, IC643, IC646, IC651, IC655, IC656, IC657, IC658, IC659, IC660, IC661, IC663, IC664, IC666, IC667, IC672, IC673, IC674, IC676, IC677, IC678, IC679, IC680, IC681, IC683, IC686, IC690, IC691, IC696, IC697, IC701, IC703, IC710, IC711, IC719, IC899, IC900 |
| H3 | IC1573, IC1580, IC1581, IC1582, IC1584, IC591, IC596, IC611, IC613, IC618, IC621, IC623, IC624, IC633, IC635, IC636 |
| H4 | IC892 |
| H5 | IC1500 |
| H6 | IC1039, IC1053 |
| H7 | IC1124 |
| H8 | IC894 |
| H9 | IC1357 |
| H10 | IC1142 |
| H11 | IC566 |
| H12 | IC317, IC318, IC319, IC320, IC321, IC322, IC323, IC324, IC325, IC326, IC327, IC328, IC329, IC330, IC331, IC33, IC38, IC494, IC526, IC53, IC58, IC59, IC60, IC65, IC66, IC67, IC69, IC71, IC72, IC76, IC800, IC801, IC804, IC805, IC806, IC807, IC808, IC809, IC814, IC816, IC822, IC828, IC832, IC837, IC839, IC840, IC848, IC853, IC854, IC860, IC863, IC867, IC876, IC908, IC909, IC910, IC911, IC912, IC913, IC915, IC916, IC917, IC918, IC919, IC923, IC924, IC926, IC927 |
|  |  |
|  |  |
| H13 | IC723, IC725, IC727, IC728, IC729, IC736, IC737, IC739, IC741, IC743, IC748, IC749, IC751, IC753, IC755, IC758, IC760, IC762, IC768, IC770, IC777, IC779, IC780, IC781, IC784, IC785, IC786, IC787, IC788, IC792, IC797, IC798 |
| H14 | IC287 |
| H15 | IC1117, IC1134, IC1140, IC1143, IC1144, IC1145, IC1146, IC1147, IC1148, IC1149, IC1150, IC1151, IC1173 |
| H16 | IC1332 |
| H17 | IC1158, IC698 |
| H18 | IC1507, IC1509 |
| H19 | IC1521 |
| H20 | IC1506 |
| H21 | IC1501 |
| H22 | IC1503, IC1504 |
| H23 | IC1522 |
| H24 | IC1502 |
| H25 | IC1514 |
| H26 | IC1518 |
| H27 | IC1508 |
| H28 | IC1516, IC1526, IC1528 |
| H29 | IC1499 |
| H30 | IC984 |

*A. alliaceus* (886-894)

*A. caelatus* (162; 560-639; 1559-1589)

*A. flavus* L (203-316; 396-475; 640-719; 899; 1179; 1027-1106; 1227; 1229-1308)

*A. flavus* S (476-479; 720-799; 1110-1178; 1228)

*A. nomius* (1493-1524)

*A. oryzae* (900-904; 1180-1214; 1216-1226)

*A. parasiticus* (1-144; 317-331; 480-559; 800-876; 905-927; 1107)

*A. sojae* (1215)

*A. tamarii* (164; 947-1026; 1309-1364; 1525-1558)

* Underlined numbers indicate evidence of trans-speciation among the majority of isolates sharing a haplotype.
