# Supplementary material for: Global population structure and adaptive evolution of aflatoxin‐producing fungi
Source: Ecol Evol. 2017 Sep 30;7(21):9179–91. doi: 10.1002/ece3.3464 (PMC5677503; doi:10.1002/ece3.3464)
Supplement: Supplementary file 26 [file ECE3-7-9179-s026.doc]

Table S14. Haplotype identities for *mfs* heuristic phylogeny for Figure S2

| Haplotype | Isolate Identities |
| --- | --- |
| H1 | IC157 |
| H2 | IC100, IC101, IC102, IC105, IC106, IC107, IC108, IC109, IC10, IC110, IC111, IC112, IC113, IC114, IC115, IC118, IC119, IC11, IC120, IC121, IC122, IC123, IC124, IC125, IC126, IC127, IC128, IC129, IC12, IC130, IC131, IC132, IC133, IC134, IC135, IC136, IC137, IC138, IC139, IC13, IC140, IC141, IC142, IC143, IC144, IC14, IC15, IC17, IC18, IC19, IC1, IC20, IC21, IC22, IC23, IC24, IC25, IC26, IC27, IC29, IC2, IC30, IC327, IC32, IC33, IC34, IC35, IC36, IC37, IC38, IC39, IC3, IC40, IC42, IC43, IC44, IC45, IC46, IC47, IC480, IC484, IC485, IC487, IC489, IC48, IC490, IC491, IC495, IC496, IC497, IC499, IC49, IC500, IC502, IC504, IC505, IC506, IC507, IC508, IC509, IC50, IC510, IC511, IC512, IC513, IC514, IC516, IC519, IC51, IC520, IC521, IC522, IC523, IC524, IC525, IC528, IC52, IC530, IC531, IC532, IC533, IC534, IC535, IC536, IC537, IC538, IC539, IC53, IC540, IC541, IC542, IC543, IC544, IC545, IC546, IC547, IC548, IC549, IC54, IC551, IC552, IC553, IC554, IC555, IC556, IC55, IC568, IC56, IC5, IC67, IC68, IC69, IC6, IC75, IC77, IC7, IC807, IC814, IC81, IC82, IC83, IC844, IC84, IC853, IC85, IC867, IC86, IC87, IC88, IC8, IC905, IC921, IC922, IC925, IC95, IC96, IC97, IC98, IC99, IC9 |
| H3 | IC1107, IC317, IC318, IC319, IC320, IC321, IC322, IC323, IC324, IC325, IC326, IC328, IC65, IC66, IC70, IC71, IC76, IC808, IC809, IC824, IC835, IC851, IC864, IC872, IC875, IC908, IC909, IC910, IC912, IC913, IC915, IC916, IC917, IC918, IC919, IC923, IC924, IC926, IC927 |
| H4 | IC825 |
| H5 | IC832, IC848 |
| H6 | IC868 |
| H7 | IC329, IC330, IC331, IC806, IC911 |
| H8 | IC494, IC58, IC59, IC60, IC72, IC74 |
| H9 | IC73, IC906, IC907, IC920 |
| H10 | IC61, IC62, IC63, IC64 |
| H11 | IC860, IC876 |
| H12 | IC800, IC801, IC804, IC805, IC816, IC822, IC837, IC839, IC840, IC854, IC863 |
| H13 | IC828 |
| H14 | IC486 |
| H15 | IC1027, IC1029, IC1030, IC1031, IC1032, IC1033, IC1034, IC1036, IC1037, IC1038, IC1040, IC1041, IC1043, IC1045, IC1046, IC1049, IC1050, IC1051, IC1056, IC1059, IC1062, IC1063, IC1065, IC1066, IC1069, IC1072, IC1073, IC1074, IC1075, IC1076, IC1077, IC1078, IC1080, IC1081, IC1086, IC1087, IC1088, IC1090, IC1092, IC1095, IC1096, IC1097, IC1098, IC1103, IC1104, IC1105, IC1152, IC1154, IC1156, IC1157, IC1158, IC1160, IC1161, IC1162, IC1163, IC1164, IC1165, IC1166, IC1167, IC1168, IC1169, IC1171, IC1174, IC1175, IC1176, IC1178, IC1179, IC1228, IC1229, IC1237, IC1239, IC1241, IC1245, IC1249, IC1250, IC1252, IC1253, IC1254, IC1255, IC1258, IC1265, IC1268, IC1269, IC1270, IC1271, IC1276, IC1280, IC1295, IC1297, IC1304, IC1305, IC1307, IC1309, IC1311, IC234, IC235, IC236, IC237, IC238, IC239, IC240, IC241, IC242, IC243, IC244, IC252, IC253, IC254, IC255, IC256, |
| H15 | IC257, IC258, IC259, IC260, IC261, IC262, IC263, IC264, IC265, IC267, IC268, IC269, IC272, IC273, IC274, IC275, IC276, IC278, IC280, IC281, IC282, IC283, IC284, IC287, IC288, IC289, IC290, IC292, IC293, IC294, IC295, IC297, IC298, IC299, IC300, IC301, IC303, IC304, IC308, IC309, IC316, IC396, IC399, IC400, IC403, IC407, IC409, IC410, IC411, IC412, IC413, IC415, IC416, IC417, IC420, IC421, IC422, IC424, IC426, IC427, IC429, IC430, IC436, IC438, IC440, IC441, IC443, IC444, IC446, IC450, IC451, IC454, IC455, IC459, IC468, IC470, IC471, IC472, IC474, IC475, IC476, IC479, IC645, IC646, IC652, IC655, IC656, IC657, IC658, IC659, IC661, IC662, IC663, IC674, IC675, IC677, IC678, IC679, IC680, IC682, IC685, IC688, IC690, IC692, IC693, IC694, IC701, IC702, IC704, IC708, IC721, IC899 |
| H16 | IC449 |
| H17 | IC1054, IC1180, IC1181, IC1184, IC1186, IC1187, IC1189, IC1194, IC1196, IC1197, IC1202, IC1207, IC1208, IC1210, IC1217, IC1218, IC1224, IC1226, IC310, IC313, IC314, IC397, IC398, IC401, IC402, IC404, IC408, IC414, IC418, IC419, IC423, IC425, IC428, IC431, IC432, IC433, IC434, IC435, IC437, IC439, IC442, IC445, IC447, IC448, IC452, IC453, IC456, IC458, IC460, IC461, IC463, IC464, IC465, IC466, IC467, IC469, IC673, IC892, IC900, IC901, IC902, IC904 |
| H18 | IC1129 |
| H19 | IC1044, IC1048, IC1082, IC1094, IC227, IC228, IC229, IC230, IC231, IC232, IC233, IC279, IC286, IC306, IC312, IC405, IC698 |
| H20 | IC1183, IC1188, IC1190, IC1193, IC1195, IC1200, IC1205, IC1206, IC1209, IC1221, IC1222, IC1223, IC903 |
| H21 | IC695 |
| H22 | IC1028, IC1035, IC1042, IC1052, IC1058, IC1060, IC1064, IC1079, IC1084, IC1085, IC1089, IC1091, IC1093, IC1099, IC1100, IC1101, IC1102, IC1106, IC1177, IC1227, IC1230, IC1233, IC1251, IC1257, IC1260, IC1262, IC1264, IC1266, IC1275, IC1277, IC1279, IC1281, IC1282, IC1290, IC1296, IC245, IC246, IC247, IC248, IC249, IC250, IC251, IC285, IC291, IC305, IC307, IC406, IC462, IC643, IC644, IC648, IC650, IC664, IC666, IC667, IC670, IC686, IC689, IC696, IC709, IC712, IC719 |
| H23 | IC753, IC760 |
| H24 | IC478, IC722, IC723, IC725, IC727, IC728, IC729, IC731, IC732, IC733, IC734, IC735, IC736, IC737, IC738, IC741, IC748, IC749, IC758, IC762, IC764, IC770, IC772, IC776, IC778, IC779, IC780, IC782, IC788, IC790, IC791, IC792, IC793, IC796, IC797 |
| H25 | IC1061, IC1083, IC1272, IC1274, IC1291, IC1293, IC1303, IC1306, IC640, IC642, IC651, IC660, IC672, IC676, IC683, IC684, IC697, IC703 |
| H26 | IC477, IC720, IC742, IC743, IC744, IC751, IC755, IC768, IC774, IC775, IC777, IC781, IC784, IC785, IC786, IC787, IC798, IC799 |
| H27 | IC1155 |
| H28 | IC1047, IC1057, IC270, IC271, IC302 |
| H29 | IC296 |
| H30 | IC691, IC711 |
| H31 | IC1039, IC1053, IC1055, IC1067, IC1068, IC1070, IC1071, IC1153, IC671 |
|  |  |
|  | |
| H32 | IC517, IC518, IC811, IC813, IC836, IC838 |
| H33 | IC1585, IC1587 |
| H34 | IC217, IC218, IC219, IC220, IC221, IC222, IC223, IC225, IC226, IC277, IC457 |
| H35 | IC311, IC315 |
| H36 | IC1539, IC1559, IC1560, IC1561, IC1562, IC1563, IC1564, IC1565, IC1566, IC1567, IC1568, IC1569, IC1570, IC1571, IC1572, IC1573, IC1574, IC1575, IC162, IC567, IC569, IC570, IC582, IC591, IC598, IC601, IC611, IC613, IC618, IC626, IC630, IC639 |
| H37 | IC1584 |
| H38 | IC565 |
| H39 | IC1577, IC1579, IC1583, IC1586 |
| H40 | IC1580, IC1581, IC1582, IC624 |
| H41 | IC1578 |
| H42 | IC596 |
| H43 | IC1112 |
| H44 | IC1117, IC1130, IC1134, IC1140, IC1144, IC1146, IC1148, IC1149, IC1150 |
| H45 | IC1124, IC1145, IC1147, IC1151 |
| H46 | IC1113, IC1118, IC1119, IC1120, IC1121, IC1133, IC1135, IC1141, IC1142 |
| H47 | IC526 |

*A. alliaceus* (886-894)

*A. caelatus* (162; 560-639; 1559-1589)

*A. flavus* L (203-316; 396-475; 640-719; 899; 1179; 1027-1106; 1227; 1229-1308)

*A. flavus* S (476-479; 720-799; 1110-1178; 1228)

*A. nomius* (157; 1493-1524)

*A. oryzae* (900-904; 1180-1214; 1216-1226)

*A. parasiticus* (1-144; 317-331; 480-559; 800-876; 905-927; 1107)

*A. sojae* (1215)

*A. tamarii* (164; 947-1026; 1309-1364; 1525-1558)

* Underlined numbers indicate evidence of trans-speciation among the majority of isolates sharing a haplotype.
