# Supplementary material for: Global population structure and adaptive evolution of aflatoxin‐producing fungi
Source: Ecol Evol. 2017 Sep 30;7(21):9179–91. doi: 10.1002/ece3.3464 (PMC5677503; doi:10.1002/ece3.3464)
Supplement: Supplementary file 27 [file ECE3-7-9179-s027.doc]

Table S15. Haplotype identities for *aflM/aflN* heuristic phylogeny in Figure S3

| Haplotype | Isolate identities |
| --- | --- |
| H1 | IC157 |
| H2 | IC477, IC720, IC731, IC733, IC735, IC742, IC744, IC778 |
| H3 | IC743, IC758 |
| H4 | IC780 |
| H5 | IC753, IC760 |
| H6 | IC793 |
| H7 | IC478, IC723, IC725, IC727, IC728, IC729, IC732, IC736, IC737, IC741, IC748, IC749, IC751, IC755, IC762, IC768, IC777, IC779, IC790, IC796, IC797 |
| H8 | IC770 |
| H9 | IC100, IC101, IC102, IC105, IC106, IC107, IC108, IC109, IC10, IC111, IC112, IC115, IC118, IC119, IC11, IC123, IC125, IC126, IC128, IC129, IC12, IC130, IC131, IC133, IC134, IC135, IC136, IC137, IC138, IC139, IC13, IC140, IC141, IC142, IC143, IC144, IC14, IC15, IC17, IC18, IC1, IC21, IC22, IC23, IC24, IC25, IC26, IC27, IC29, IC2, IC327, IC32, IC33, IC34, IC35, IC36, IC37, IC38, IC39, IC40, IC42, IC44, IC46, IC480, IC484, IC485, IC486, IC487, IC489, IC48, IC491, IC495, IC496, IC497, IC499, IC49, IC500, IC504, IC505, IC506, IC507, IC508, IC509, IC50, IC510, IC511, IC512, IC514, IC516, IC519, IC51, IC520, IC521, IC522, IC523, IC524, IC525, IC528, IC529, IC52, IC530, IC531, IC532, IC533, IC534, IC535, IC536, IC537, IC538, IC539, IC53, IC540, IC541, IC542, IC543, IC544, IC545, IC546, IC547, IC548, IC549, IC551, IC552, IC553, IC554, IC555, IC556, IC55, IC56, IC58, IC59, IC5, IC60, IC61, IC62, IC63, IC64, IC67, IC68, IC69, IC72, IC74, IC75, IC77, IC7, IC800, IC801, IC804, IC805, IC807, IC814, IC816, IC81, IC822, IC824, IC828, IC835, IC83, IC840, IC844, IC84, IC853, IC854, IC868, IC86, IC875, IC88, IC8, IC905, IC921, IC922, IC925, IC95, IC97, IC98, IC99 |
| H10 | IC1027, IC1029, IC1030, IC1031, IC1032, IC1034, IC1040, IC1046, IC1047, IC1054, IC1057, IC1058, IC1062, IC1069, IC1077, IC1086, IC1088, IC1092, IC1095, IC1100, IC1102, IC1105, IC1229, IC1237, IC1245, IC1252, IC1253, IC1255, IC1268, IC1269, IC1270, IC1276, IC1295, IC1297, IC1307, IC203, IC204, IC217, IC218, IC219, IC220, IC221, IC222, IC223, IC225, IC226, IC234, IC237, IC238, IC239, IC240, IC241, IC242, IC243, IC244, IC258, IC259, IC260, IC261, IC262, IC270, IC271, IC272, IC273, IC274, IC275, IC277, IC278, IC280, IC281, IC282, IC284, IC287, IC290, IC292, IC293, IC294, IC295, IC297, IC298, IC299, IC300, IC301, IC302, IC303, IC304, IC307, IC308, IC313, IC409, IC410, IC411, IC413, IC415, IC416, IC417, IC420, IC421, IC424, IC427, IC436, IC440, IC443, IC457, IC459, IC468, IC470, IC471, IC472, IC476, IC479, IC646, IC652, IC655, IC656, IC657, IC658, IC659, IC674, IC678, IC680, IC682, IC685, IC688, IC695, IC701, IC702, IC704, IC708, IC899 |
| H11 | IC1154, IC1155, IC1156, IC1157, IC1160, IC1161, IC1163, IC1164, IC1165, IC1167, IC1168, IC1169, IC1171, IC1174, IC1175, IC1176, IC1177, IC1178, IC1228 |
| H12 | IC1162 |
| H13 | IC1180, IC1184, IC1187, IC1194, IC1202, IC1207, IC1210, IC1217, IC1224, IC1226, IC900, IC902 |
| H14 | IC1250 |
| H15 | IC1153 |
| H16 | IC1145, IC1147, IC1151 |
| H17 | IC1078 |
| H18 | IC1080 |
| H19 | IC1112 |
| H20 | IC422 |
| H21 | IC446 |
| H22 | IC1076, IC426, IC475 |
| H23 | IC1051 |
| H24 | IC1075, IC450, IC675 |
| H25 | IC396, IC403, IC430, IC438, IC441, IC451, IC454, IC455, IC474, IC640, IC642, IC651, IC660, IC672, IC676, IC683, IC684, IC697, IC703 |
| H26 | IC110, IC513 |
| H27 | IC1140 |
| H28 | IC502 |
| H29 | IC54 |
| H30 | IC47 |
| H31 | IC329, IC330, IC911 |
| H32 | IC806 |
| H33 | IC517, IC518, IC811, IC813, IC836, IC860, IC876 |
| H34 | IC73, IC906, IC907, IC920 |
| H35 | IC832, IC848 |
| H36 | IC331 |
| H37 | IC808, IC864, IC872 |
| H38 | IC1144, IC1146, IC1148, IC1149, IC1150 |
| H39 | IC1028, IC1035, IC1036, IC1038, IC1042, IC1044, IC1048, IC1049, IC1056, IC1063, IC1065, IC1066, IC1072, IC1073, IC1079, IC1087, IC1089, IC1090, IC1091, IC1093, IC1094, IC1096, IC1099, IC1101, IC1103, IC1152, IC1227, IC1230, IC1233, IC1251, IC1254, IC1258, IC1260, IC1262, IC1264, IC1265, IC1266, IC1275, IC1277, IC1281, IC1305, IC227, IC228, IC229, IC232, IC233, IC253, IC263, IC264, IC265, IC267, IC268, IC269, IC279, IC283, IC285, IC286, IC288, IC305, IC306, IC312, IC405, IC406, IC462, IC490, IC643, IC650, IC661, IC662, IC663, IC677, IC679, IC686, IC696, IC698, IC711, IC719 |
| H40 | IC1074 |
| H41 | IC863 |
| H42 | IC399 |
| H43 | IC1039, IC1043, IC1045, IC1053, IC1055, IC1067, IC1068, IC1070, IC1071, IC1083, IC1272, IC1306, IC671 |
| H44 | IC1106 |
| H45 | IC1084, IC1085 |
|  |  |
| H46 | IC1033, IC1037, IC1041, IC1059, IC1081, IC1097, IC1098, IC1241, IC1271, IC1280, IC276, IC289, IC296, IC400, IC407, IC412, IC429, IC444 |
| H47 | IC1179, IC1239, IC1304, IC309 |
| H48 | IC1052, IC1060, IC1257, IC1279, IC1282, IC1290, IC1296, IC291, IC648, IC664, IC666, IC667, IC670, IC709, IC712 |
| H49 | IC1050, IC1064, IC1082 |
| H50 | IC1249 |
| H51 | IC245, IC248, IC249, IC250, IC251 |
| H52 | IC1104 |
| H53 | IC458 |
| H54 | IC1221 |
| H55 | IC1113, IC1118, IC1119, IC1120, IC1121, IC1133, IC1141 |
| H56 | IC1183 |
| H57 | IC1222 |
| H58 | IC1142 |
| H59 | IC1117 |
| H60 | IC1134 |
| H61 | IC1135 |
| H62 | IC448 |
| H63 | IC1061 |
| H64 | IC867 |
| H65 | IC1274, IC1291, IC1293, IC1303 |
| H66 | IC1181, IC1186, IC1188, IC1189, IC1190, IC1193, IC1195, IC1196, IC1200, IC1205, IC1206, IC1209, IC1218, IC1223, IC901, IC903, IC904 |
| H67 | IC431 |
| H68 | IC439 |
| H69 | IC469 |
| H70 | IC310, IC397, IC398, IC401, IC402, IC404, IC408, IC414, IC418, IC419, IC423, IC425, IC428, IC432, IC433, IC434, IC435, IC437, IC442, IC445, IC447, IC449, IC452, IC453, IC456, IC460, IC461, IC463, IC464, IC465, IC466, IC467, IC673 |
| H71 | IC837 |
| H72 | IC839 |
| H73 | IC785 |
| H74 | IC786 |
| H75 | IC788 |
| H76 | IC791 |
| H77 | IC787 |
| H78 | IC792, IC799 |
| H79 | IC798 |
| H80 | IC851 |
| H81 | IC886, IC889, IC891, IC894 |
| H82 | IC96 |
| H83 | IC1215, IC494, IC526 |
| H84 | IC19 |
| H85 | IC1107, IC328 |
| H86 | IC825 |
| H87 | IC317, IC318, IC319, IC320, IC321, IC322, IC323, IC324, IC325, IC326, IC65, IC66, IC71, IC76, IC809, IC908, IC909, IC910, IC912, IC913, IC915, IC916, IC917, IC918, IC919, IC923, IC924, IC926, IC927 |
| H88 | IC70 |

*A. alliaceus* (886-894)

*A. caelatus* (162; 560-639; 1559-1589)

*A. flavus* L (203-316; 396-475; 640-719; 899; 1179; 1027-1106; 1227; 1229-1308)

*A. flavus* S (476-479; 720-799; 1110-1178; 1228)

*A. nomius* (157; 1493-1524)

*A. oryzae* (900-904; 1180-1214; 1216-1226)

*A. parasiticus* (1-144; 317-331; 480-559; 800-876; 905-927; 1107)

*A. sojae* (1215)

*A. tamarii* (164; 947-1026; 1309-1364; 1525-1558)

* Underlined numbers indicate evidence of trans-speciation among the majority of isolates sharing a haplotype.
