# Supplementary material for: Global population structure and adaptive evolution of aflatoxin‐producing fungi
Source: Ecol Evol. 2017 Sep 30;7(21):9179–91. doi: 10.1002/ece3.3464 (PMC5677503; doi:10.1002/ece3.3464)
Supplement: Supplementary file 28 [file ECE3-7-9179-s028.doc]

Table S16. Haplotype identities for *aflW/aflX* heuristic phylogeny in Figure S4

| Haplotype | Isolate identities |
| --- | --- |
| H1 | IC157, IC891, IC892 |
| H2 | IC1117 |
| H3 | IC1144, IC1146, IC1148, IC1149, IC1150 |
| H4 | IC1140 |
| H5 | IC100, IC101, IC102, IC105, IC106, IC107, IC108, IC109, IC10, IC110, IC111, IC112, IC115, IC118, IC119, IC11, IC123, IC125, IC126, IC128, IC129, IC12, IC130, IC131, IC133, IC134, IC135, IC136, IC137, IC138, IC139, IC13, IC140, IC141, IC142, IC143, IC144, IC14, IC15, IC17, IC18, IC19, IC1, IC21, IC22, IC23, IC24, IC25, IC26, IC27, IC29, IC2, IC327, IC32, IC33, IC34, IC35, IC36, IC37, IC38, IC39, IC40, IC42, IC52, IC53, IC54, IC55, IC56, IC5, IC67, IC68, IC69, IC75, IC77, IC7, IC81, IC83, IC84, IC86, IC88, IC8, IC921, IC95, IC96, IC97, IC98, IC99, IC484, IC486, IC487, IC489, IC490, IC491, IC496, IC497, IC502, IC504, IC505, IC506, IC507, IC508, IC509, IC510, IC512, IC514, IC516, IC520, IC521, IC522, IC529, IC530, IC532, IC533, IC534, IC535, IC540, IC541, IC542, IC543, IC544, IC545, IC548, IC549, IC553, IC554, IC555, IC565, IC566, IC567, IC568, IC570, IC571, IC807, IC814, IC844, IC853, IC867 |
| H6 | IC317, IC318, IC319, IC320, IC321, IC322, IC323, IC324, IC325, IC326, IC328, IC58, IC59, IC60, IC61, IC62, IC63, IC64, IC65, IC66, IC70, IC71, IC72, IC74, IC76, IC908, IC909, IC910, IC911, IC912, IC913, IC915, IC916, IC917, IC918, IC919, IC923, IC924, IC926, IC927, IC1107, IC800, IC801, IC804, IC805, IC808, IC809, IC816, IC822, IC824, IC825, IC828, IC835, IC837, IC839, IC840, IC851, IC854, IC863, IC864, IC868, IC872, IC875 |
| H7 | IC591 |
| H8 | IC44, IC46, IC47, IC48, IC49, IC50, IC51, IC905, IC922, IC925, IC480, IC485, IC495, IC499, IC500, IC511, IC513, IC519, IC523, IC524, IC525, IC528, IC531, IC536, IC537, IC538, IC539, IC546, IC547, IC551, IC552, IC556, IC561 |
| H9 | IC329, IC330, IC331, IC906, IC907, IC920, IC806 |
| H10 | IC832, IC848 |
| H11 | IC73 |
| H12 | IC1198, IC1215, IC1216, IC1225, IC164, IC526 |
| H13 | IC296 |
| H14 | IC1112 |
| H15 | IC162 |
| H16 | IC1185, IC1188, IC1190, IC1195, IC1200, IC1204, IC1205, IC1206, IC1209, IC1221, IC1222, IC1223, IC903 |
| H17 | IC517, IC518 |
| H18 | IC1145, IC1147, IC1151 |
| H19 | IC477, IC478, IC720, IC742, IC743, IC744, IC785, IC786, IC787, IC790, IC791, IC799 |
| H20 | IC611, IC723, IC725, IC727, IC728, IC729, IC731, IC732, IC733, IC735, IC736, IC737, IC741, IC748, IC749, IC758, IC762, IC770, IC778, IC779, |
| H20 | IC780, IC788, IC792, IC793, IC796, IC797 |
| H21 | IC751, IC755, IC768, IC777, IC798 |
| H22 | IC753, IC760 |
| H23 | IC494 |
| H24 | IC811, IC813, IC836, IC860, IC876 |
| H25 | IC1203 |
| H26 | IC422 |
| H27 | IC1355 |
| H28 | IC1214 |
| H29 | IC1054, IC1154, IC1156, IC1157, IC1160, IC1161, IC1162, IC1163, IC1164, IC1165, IC1167, IC1168, IC1169, IC1171, IC1174, IC1175, IC1176, IC1178, IC1228, IC313, IC316, IC598, IC618, IC888, IC890 |
| H30 | IC1199 |
| H31 | IC1212 |
| H32 | IC626 |
| H33 | IC308, IC476, IC479 |
| H34 | IC1061, IC1274, IC1291, IC1293, IC1303, IC217, IC218, IC219, IC220, IC221, IC222, IC223, IC225, IC277, IC310, IC311, IC314, IC315, IC397, IC398, IC401, IC402, IC404, IC408, IC414, IC418, IC419, IC423, IC425, IC428, IC431, IC432, IC433, IC434, IC435, IC437, IC439, IC442, IC445, IC447, IC448, IC449, IC452, IC453, IC454, IC456, IC457, IC458, IC460, IC461, IC463, IC464, IC465, IC466, IC469, IC617, IC673 |
| H35 | IC639 |
| H36 | IC624, IC640, IC642, IC651, IC660, IC672, IC676, IC683, IC684, IC697, IC703 |
| H37 | IC1211 |
| H38 | IC1155 |
| H39 | IC1180, IC1184, IC1187, IC1194, IC1197, IC1202, IC1207, IC1208, IC1210, IC1217, IC1224, IC1226, IC902 |
| H40 | IC1027, IC1030, IC1031, IC1032, IC1037, IC1038, IC1040, IC1041, IC1046, IC1047, IC1051, IC1057, IC1059, IC1062, IC1066, IC1080, IC1081, IC1087, IC1088, IC1092, IC1094, IC1229, IC1237, IC1250, IC1252, IC1254, IC1255, IC1268, IC1269, IC1276, IC1295, IC1307, IC1310, IC1311, IC1325, IC1345, IC1353, IC203, IC204, IC234, IC237, IC238, IC239, IC240, IC241, IC242, IC243, IC253, IC267, IC268, IC269, IC270, IC271, IC272, IC273, IC280, IC281, IC284, IC288, IC289, IC290, IC293, IC294, IC297, IC298, IC299, IC300, IC301, IC302, IC409, IC410, IC413, IC415, IC417, IC421, IC424, IC427, IC436, IC440, IC451, IC459, IC468, IC470, IC471, IC646, IC652, IC655, IC656, IC657, IC658, IC659, IC674, IC675, IC695, IC701, IC708, IC900 |
| H41 | IC1113, IC1118, IC1119, IC1120, IC1121, IC1133, IC1134, IC1135, IC1141, IC1142, IC1191 |
| H42 | IC1152, IC1179, IC1239, IC1270, IC1304, IC244, IC258, IC259, IC260, IC261, IC262, IC278, IC287, IC292, IC303, IC304, IC309, IC411, IC416, IC420, IC426, IC443, IC446, IC450, IC472, IC475, IC643, IC679, IC899 |
| H43 | IC1309 |
| H44 | IC1280 |
| H45 | IC1039, IC1053, IC1068, IC1070 |
| H46 | IC1067 |
| H47 | IC1084, IC1085, IC1086 |
| H48 | IC1044, IC1048, IC1049, IC1050, IC1082, IC1090, IC1105, IC1106, IC1153, IC1177, IC1305, IC226, IC227, IC228, IC229, IC232, IC233, IC279, IC286, IC306, IC312, IC396, IC399, IC403, IC405, IC430, IC438, IC441, IC455, IC474, IC580, IC662, IC677, IC698, IC711 |
| H49 | IC1043, IC1045, IC1055, IC1071, IC1083, IC1272, IC1306, IC245, IC248, IC249, IC250, IC251, IC254, IC307, IC467, IC671 |
| H50 | IC1028, IC1033, IC1035, IC1036, IC1042, IC1052, IC1056, IC1058, IC1060, IC1063, IC1064, IC1065, IC1072, IC1073, IC1074, IC1079, IC1089, IC1091, IC1093, IC1095, IC1096, IC1098, IC1099, IC1100, IC1101, IC1102, IC1103, IC1104, IC1219, IC1227, IC1230, IC1233, IC1241, IC1249, IC1251, IC1257, IC1258, IC1260, IC1262, IC1264, IC1265, IC1266, IC1271, IC1275, IC1277, IC1279, IC1281, IC1282, IC1290, IC1296, IC1496, IC1497, IC1498, IC1502, IC1504, IC1506, IC1507, IC1508, IC1510, IC1511, IC1512, IC1517, IC1518, IC1520, IC1521, IC1522, IC1523, IC1524, IC1525, IC1526, IC1529, IC1530, IC1531, IC1532, IC1534, IC1535, IC1537, IC1538, IC1539, IC1540, IC1541, IC1542, IC1543, IC1545, IC1548, IC1549, IC1550, IC1552, IC1553, IC1554, IC1556, IC1557, IC1559, IC1560, IC1561, IC1562, IC1563, IC1564, IC1566, IC1567, IC1569, IC1571, IC1572, IC1573, IC1574, IC1575, IC1577, IC1578, IC1580, IC1581, IC1582, IC1584, IC1585, IC1586, IC1587, IC263, IC264, IC265, IC276, IC283, IC285, IC291, IC305, IC406, IC462, IC648, IC650, IC661, IC663, IC664, IC666, IC667, IC670, IC686, IC696, IC709, IC712, IC719, IC901 |
| H51 | IC1533 |
| H52 | IC400, IC407, IC412, IC429, IC444 |
| H53 | IC1029, IC1034, IC1069, IC1075, IC1076, IC1077, IC1078, IC1097, IC1245, IC1253, IC1297, IC274, IC275, IC282, IC295, IC678, IC680, IC682, IC685, IC688, IC702, IC704 |
| H54 | IC889 |
| H55 | IC1181, IC1182, IC1183, IC1186, IC1189, IC1193, IC1196, IC1218, IC904 |

*A. alliaceus* (886-894)

*A. caelatus* (162; 560-639; 1559-1589)

*A. flavus* L (203-316; 396-475; 640-719; 899; 1179; 1027-1106; 1227; 1229-1308)

*A. flavus* S (476-479; 720-799; 1110-1178; 1228)

*A. nomius* (157; 1493-1524)

*A. oryzae* (900-904; 1180-1214; 1216-1226)

*A. parasiticus* (1-144; 317-331; 480-559; 800-876; 905-927; 1107)

*A. sojae* (1215)

*A. tamarii* (164; 947-1026; 1309-1364; 1525-1558)

* Underlined numbers indicate evidence of trans-speciation among the majority of isolates sharing a haplotype.
